# Supplementary material for: Molecular condensation of the CO/NF-YB/NF-YC/FT complex gates floral transition in Arabidopsis
Source: EMBO J. 2024 Nov 20;44(1):225–50. doi: 10.1038/s44318-024-00293-0 (PMC11696179; doi:10.1038/s44318-024-00293-0)
Supplement: Supplementary file 1 — Appendix [file 44318_2024_293_MOESM1_ESM.pdf]

Appendix

Molecular condensation of the CO/NF-YB/NF-YC/FT complex gates floral transition in Arabidopsis

Xiang Huang, Zhiming Ma, Danxia He, Xiao Han, Xu Liu, Qiong Dong, Cuirong Tan, Bin Yu, Tiedong Sun, Lars Nordenskiöld, Lanyuan Lu, Yansong Miao, Xingliang Hou

Table of contents

Appendix Figure S1……………………………………………………………………page 2
Appendix Figure S2……………………………………………………………………page 3
Appendix Figure S3……………………………………………………………………page 4
Appendix Figure S4……………………………………………………………………page 5
Appendix Table S1……………………………………………………………………..page 6
Appendix Table S2…………………………………………………………………… page 7&8

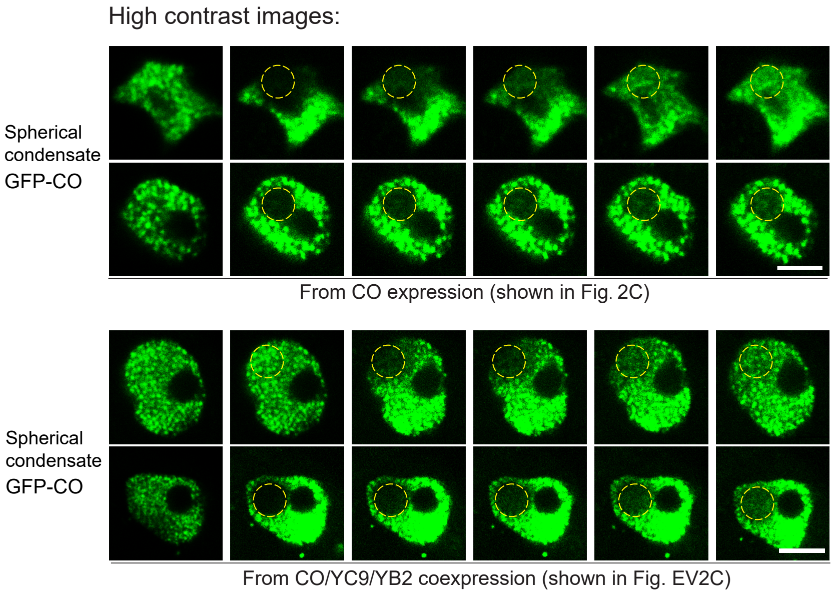

Appendix Figure S1. **High contrast images of those shown in Fig. 2C and Fig. EV2C.**

The image contrast was adjusted to highlight the CO signal, either cluster or diffused signal, in the bleached area. Scale bars, 5 µm. Dash circles indicate the bleached areas.

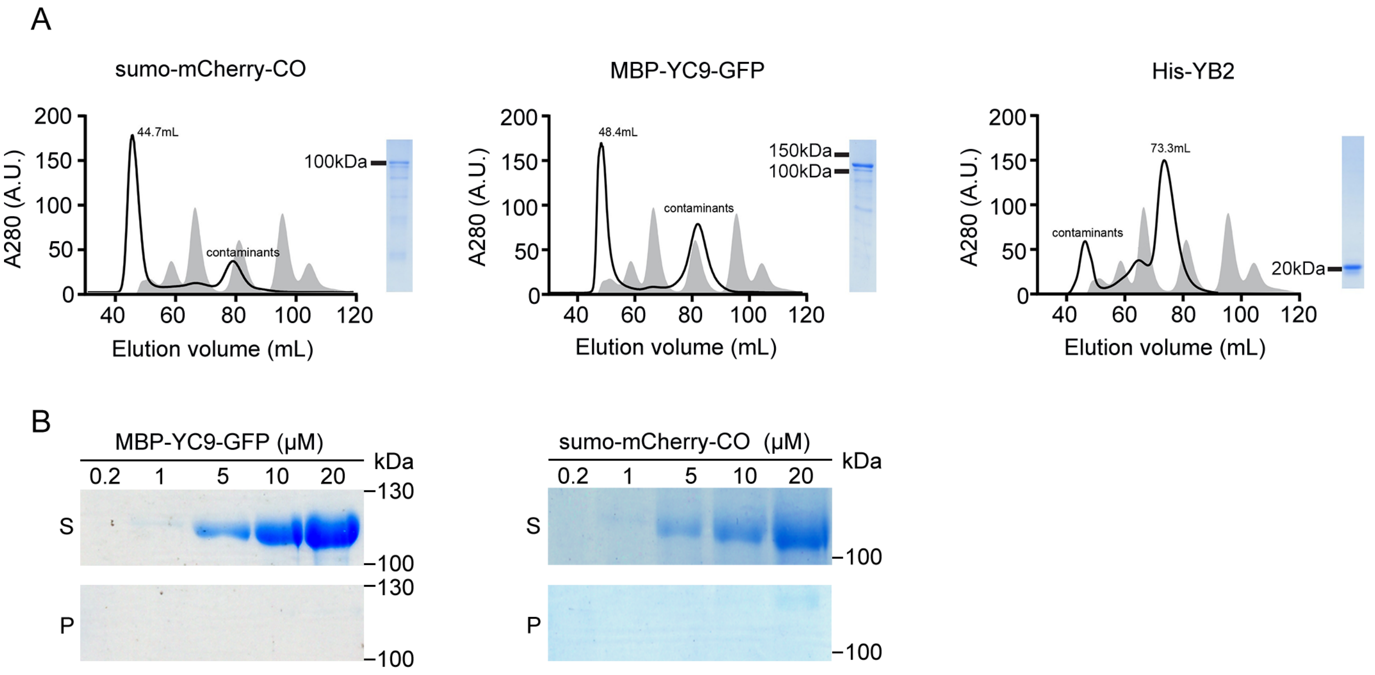

Appendix Figure S2. Characterizations of recombinant mCherry-CO, NF-YC9-GFP, and NF-YB2 proteins.

A. Size exclusion chromatography and SDS-PAGE gels of recombinant mCherry-CO, NF-YC9-GFP, and NF-YB2. Size exclusion was performed by Superdex 200pg, 16/600 column. Grey curves indicated the elution volumes of the standard proteins: Ferritin (443 kDa, the first two peaks), beta-Amylase (200 kDa), Conalbumin (75 kDa), Carbonic Anhydrase (29 kDa), RibonucleaseA (13.7 kDa)). Target proteins were showed with black curves. The peaks with volume number represented targets proteins eluted at the indicated volume, while other lower peaks indicated contaminants.

B. Recombinant mCherry-CO and NF-YC9-GFP are both soluble in the experimental buffer (20 mM HEPES, 150 mM NaCl, pH7.4). The protein stocks were diluted to the indicated concentrations by experimental buffer, and then subjected to ultracentrifugation (100,000 x *g*) for 30 min, followed by the separation of supernatant (S) from pellet (P) and the latter was further resuspended by the same volume of buffer. 5 µL of supernatant or pellet were then loaded and separated in SDS-PAGE gel.

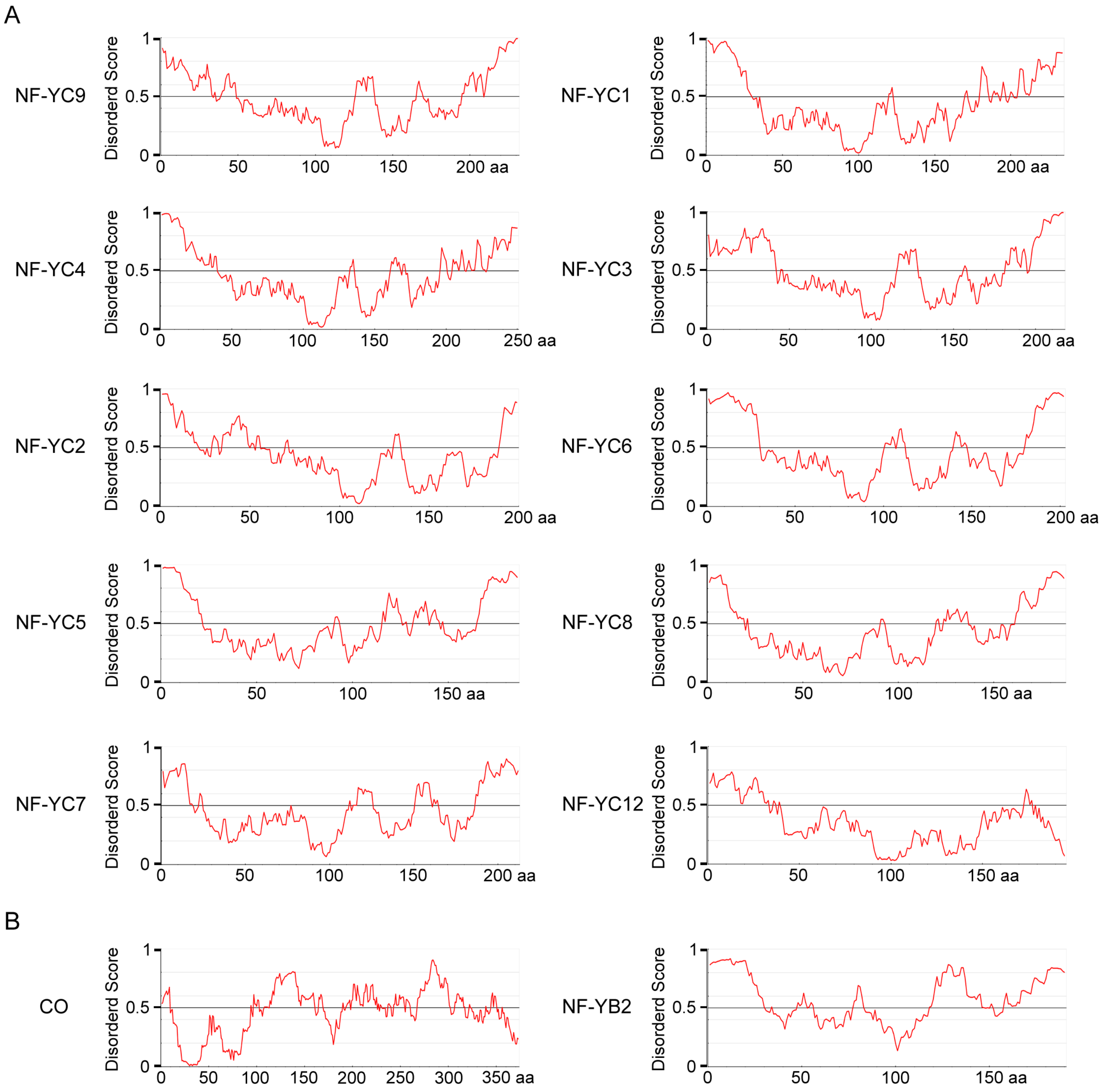

Appendix Figure S3. Sequence analysis of NF-YC family members, CO, and NF-YB2 in *Arabidopsis thaliana*

A. Protein sequence intrinsically disordered prediction of NF-YC family members in *Arabidopsis thaliana* was performed using the IUPRED2A algorithm. IDR score larger than 0.5 is regarded as an intrinsically disordered region.
B. Protein sequence intrinsically disordered prediction of CO and NF-YB2 was performed using the IUPRED2A algorithm. IDR score larger than 0.5 is regarded as an intrinsically disordered region.

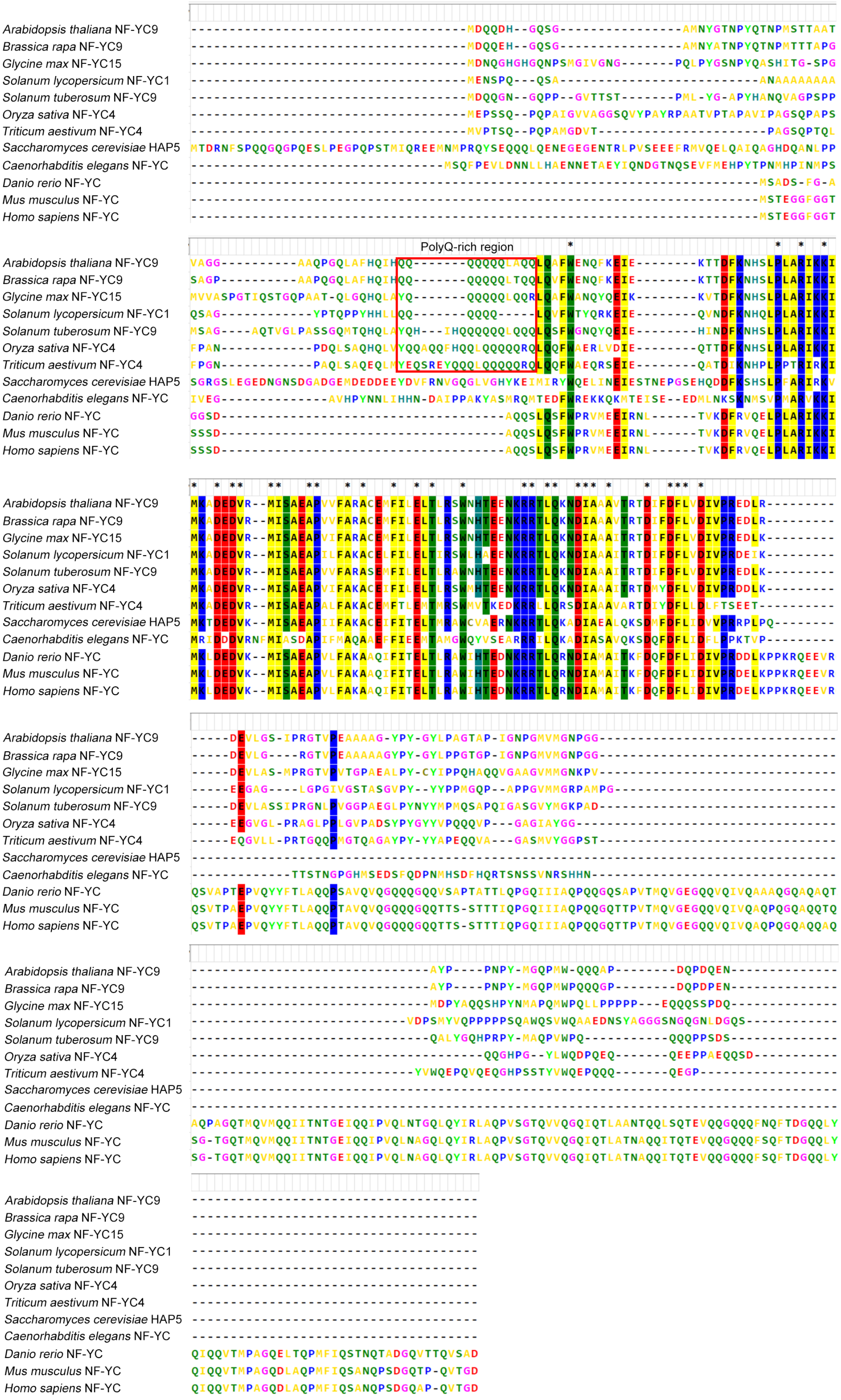

Appendix Figure S4. Protein sequence alignment of NF-YC9 and its closest homologues in other species

Amino acid sequence alignment of NF-YC9 in *Arabidopsis thaliana* and its closest homologues in other plant species, yeast, animals and human. These sequences were retrieved from TAIR or NCBI. The polyQ-rich region is indicated in the red box.

Appendix Table S1. Arabidopsis transgenic lines used in this study

Name	Source
Arabidopsis: Col-0	N/A
Arabidopsis: *nf-yc3 nf-yc4 nf-yc9* (*ycT*)	(Tang et al., 2017)*
Arabidopsis: *35S:mCherry-CO*	This paper
Arabidopsis: *35S:mCherry-CO ycT*	This paper
Arabidopsis: *35S:NF-YC9-GFP ycT*	This paper
Arabidopsis: *35S:mCherry-CO 35S:NF-YC9-GFP ycT*	This paper
Arabidopsis: *35S:NF-YC9-∆IDR1-GFP ycT*	This paper
Arabidopsis: *35S:NF-YC9-∆IDR2-GFP ycT*	This paper
Arabidopsis: *35S:NF-YC9-∆IDR1&2-GFP ycT*	This paper
Arabidopsis: *35S:NF-YC9-0Q-GFP ycT*	This paper
Arabidopsis: *35S:NF-YC9-37Q-GFP ycT*	This paper

* Tang, Y. et al. Arabidopsis NF-YCs mediate the light-controlled hypocotyl elongation via modulating histone acetylation. *Mol. Plant* **10**, 260-273 (2017).

Appendix Table S2. List of primers sequences used in this study

Primers for constructs in plant transformation

Construct name	Primers (5'-3')
35S:mCherry-CO	F: AAGCTTGATATCGAATTCATGTTGAAACAAGAGAGT
	R: TCTAGAACTAGTGGATCCGAATGAAGGAACAATCCC
35S:NF-YC9-GFP	F: CTGCAGCCCGGGGGATCCATGGATCAACAAGACCAT
	R: TCCTTTACTCATACTAGTATTTTCCTGGTCAGGTTG
35S:NF-YC9-ΔIDR1-GFP	F: CTGCAGCCCGGGGGATCCATGCTGGCACAGCAGCTT
	R: TTCTTCTCCTTTACTCATACTAGTATTTTCCTGGTC
35S:NF-YC9-ΔIDR2-GFP	F: CTGCAGCCCGGGGGATCCATGGATCAACAAGACCAT
	R: TTCTTCTCCTTTACTCATACTAGTCAAGACTTCATC
35S:NF-YC9-ΔIDR1&2-GFP	F: CTGCAGCCCGGGGGATCCATGCTGGCACAGCAGCTT
	R: TTCTTCTCCTTTACTCATACTAGTCAAGACTTCATC
35S:NF-YC9-0Q-GFP	F1: CTGCAGCCCGGGGGATCCATGGATCAACAAGACCAT
	R1: TTGAAGCTGCTGTGCCAGATGGATCTGGTGGAACGC
	F2: GCGTTCCACCAGATCCATCTGGCACAGCAGCTTCAA
	R2: TCCTTTACTCATACTAGTATTTTCCTGGTCAGGTTG
35S:NF-YC9-37Q-GFP	(The former part containing extended polyQ repeats sequence is synthesized by company) AATTCCTGCAGCCCGGGGGATCCATGGATCAACAAGACCATGGACAGTCTGG AGCTATGAACTATGGCACAAACCCATACCAAACCAACCCGATGAGCACCACTG CTGCTACTGTAGCAGGAGGTGCGGCACAACCAGGCCAGCTGGCGTTCCACCA GATCCATCAGCAGCAGCAGCAGCAACAGCAGCAGCAGCAGCAGCAACAGCAG CAGCAGCAGCAGCAACAGCAGCAGCAGCAGCAGCAACAGCAGCAGCAGCAGC AGCAGCAGCAACAGCTGGCACAGCAGCTTCAAGCATTTTGGGAGAACCAA
	F2: GCATTTTGGGAGAACCAATTCAAAGAGATTGAGAAG
	R2: TCCTTTACTCATACTAGTATTTTCCTGGTCAGGTTG

Primers for constructs *in vivo* protein distribution

Construct name	Primers (5'-3')
35S:GFP-CO	F: AAGCTTGATATCGAATTCATGTTGAAACAAGAGAGT
	R: TCTAGAACTAGTGGATCCGAATGAAGGAACAATCCC
35S:GFP-CO-ΔB-box	F1: CAAGCTACGCGTCTCGAGATGAGTAAAGGA
	R1: TTCCAGAAATTGGTAGGTTCTCTCCACTACC
	F2: GGTAGTGGAGAGAACCTACCAATTTCTGGAA
	R2: CTAGAACTAGTGGATCCGAATGAAGGAACAA
35S:NF-YC9-mCherry	F: CAAGCTACGCGTCTCGAGATGGATCAACAAGACCAT
	R: GAATTCGATATCAAGCTTATTTTCCTGGTCAGGTTG
35S:NF-YC9-ΔIDR1-mCherry	F: CTGCAGCCCGGGGGATCCATGCTGGCACAGCAGCTT
	R: CTTGCTCACCATACTAGTATTTTCCTGGTCAGGTTG
35S:NF-YC9-ΔIDR2-mCherry	F: CTGCAGCCCGGGGGATCCATGGATCAACAAGACCAT
	R: CTTGCTCACCATACTAGTCAAGACTTCATCTCGGAG
35S:NF-YC9-ΔIDR1&2-mCherry	F: CTGCAGCCCGGGGGATCCATGCTGGCACAGCAGCTT
	R: CTTGCTCACCATACTAGTCAAGACTTCATCTCGGAG
35S:NF-YC9-0Q-mCherry	F: CTGCAGCCCGGGGGATCCATGGATCAACAAGACCAT
	R: CTTGCTCACCATACTAGTATTTTCCTGGTCAGGTTG
35S:NF-YC9-37Q-mCherry	F: CTGCAGCCCGGGGGATCCATGGATCAACAAGACCAT
	R: CTTGCTCACCATACTAGTATTTTCCTGGTCAGGTTG
35S:NF-YB2-BFP	F: CAAGCTACGCGTCTCGAGATGGGGGATTCCGACAGG
	R: GAATTCGATATCAAGCTTAGTCCTTGTCCTACCGGA

Primers for constructs *in vitro* protein expression and purification

Construct name	Primers (5'-3')
SUMO-mCherry-CO	F: CAGGAACAAACGGGGGGTATGGTGAGCAAGGGCGAG
	R: GTTCAGACCGCCACCGCTTCAGAATGAAGGAACAAT
MBP-NF-YC9-GFP	F: AACGGCAGCAGCGGATCCATGGATCAACAAGACCAT
	R: GTGCTCGAGTGCGGCCGCTTATTTGTATAGTTCTAC
His-NF-YB2	F: CATCACCATCACGGATCCATGGGGGATTCCGACAGG
	R: GCTTGGCTGCAGGTCGACTTAAGTCCTTGTCCTACC

**Primers for quantitative real-time PCR**	
Construct name	Primers (5'-3')
FT	F: CTGGAACAACCTTTGGCAAT
	R: TACACTGTTTGCCTGCCAAG
UBQ10	F: AGGATGGCAGAACTCTTGCT
	R: TCCCAGTCAACGTCTTAACG

**Primers for constructs in transient expression assay**	
Construct name	Primers (5'-3')
35S:GFP-CO	F: AAGCTTGATATCGAATTCATGTTGAAACAAGAGAGT
	R: TCTAGAACTAGTGGATCCGAATGAAGGAACAATCCC
35S:NF-YC9- mCherry	F: CAAGCTACGCGTCTCGAGATGGATCAACAAGACCAT
	R: GAATTCGATATCAAGCTTATTTTCCTGGTCAGGTTG
35S:NF-YC9-0Q-mCherry	F: CTGCAGCCCGGGGGATCCATGGATCAACAAGACCAT
	R: CTTGCTCACCATACTAGTATTTTCCTGGTCAGGTTG
35S:NF-YC9-37Q-mCherry	F: CTGCAGCCCGGGGGATCCATGGATCAACAAGACCAT
	R: CTTGCTCACCATACTAGTATTTTCCTGGTCAGGTTG
35S: NF-YB2-BFP	F: CAAGCTACGCGTCTCGAGATGGGGGATTCCGACAGG
	R: GAATTCGATATCAAGCTTAGTCCTTGTCCTACCGGA
pFT:GUS	F: GACGGTATCGATAAGCTTCATTTGCTGAACAAAAAT
	R: CCCGGGCTGCAGGAATTCATCATAGGCATGAACCCT

**Primers for constructs in yeast two-hybrid assay**	
Construct name	Primers (5'-3')
AD-CO	F: ATGGAGGCCAGTGAATTCATGTTGAAACAAGAGAGT
	R: CTCGAGCTCGATGGATCCTCAGAATGAAGGAACAAT
BD-NF-YC9	F: GAGGAGGACCTGCATATGATGGATCAACAAGACCAT
	R: ACGGATCCCCGGGAATTCTTAATTTTCCTGGTCAGG
BD-NF-YC9-ΔIDR1	F: GAGGAGGACCTGCATATGATGCTGGCACAGCAGCTT
	R: ACGGATCCCCGGGAATTCTTAATTTTCCTGGTCAGG
BD-NF-YC9-ΔIDR2	F: GAGGAGGACCTGCATATGATGGATCAACAAGACCAT
	R: ACGGATCCCCGGGAATTCTTACAAGACTTCATCTCG
BD-NF-YC9-ΔIDR1&2	F: GAGGAGGACCTGCATATGATGCTGGCACAGCAGCTT
	R: ACGGATCCCCGGGAATTCTTACAAGACTTCATCTCG
BD-NF-YC9-0Q	F: GAGGAGGACCTGCATATGATGGATCAACAAGACCAT
	R: ACGGATCCCCGGGAATTCTTAATTTTCCTGGTCAGG
BD-NF-YC9-37Q	F: GAGGAGGACCTGCATATGATGGATCAACAAGACCAT
	R: ACGGATCCCCGGGAATTCTTAATTTTCCTGGTCAGG
