# Supplementary figures and images for: Molecular condensation of the CO/NF-YB/NF-YC/FT complex gates floral transition in Arabidopsis (part 1 of 3)
Source: EMBO J. 2024 Nov 20;44(1):225–50. doi: 10.1038/s44318-024-00293-0 (PMC11696179; doi:10.1038/s44318-024-00293-0)

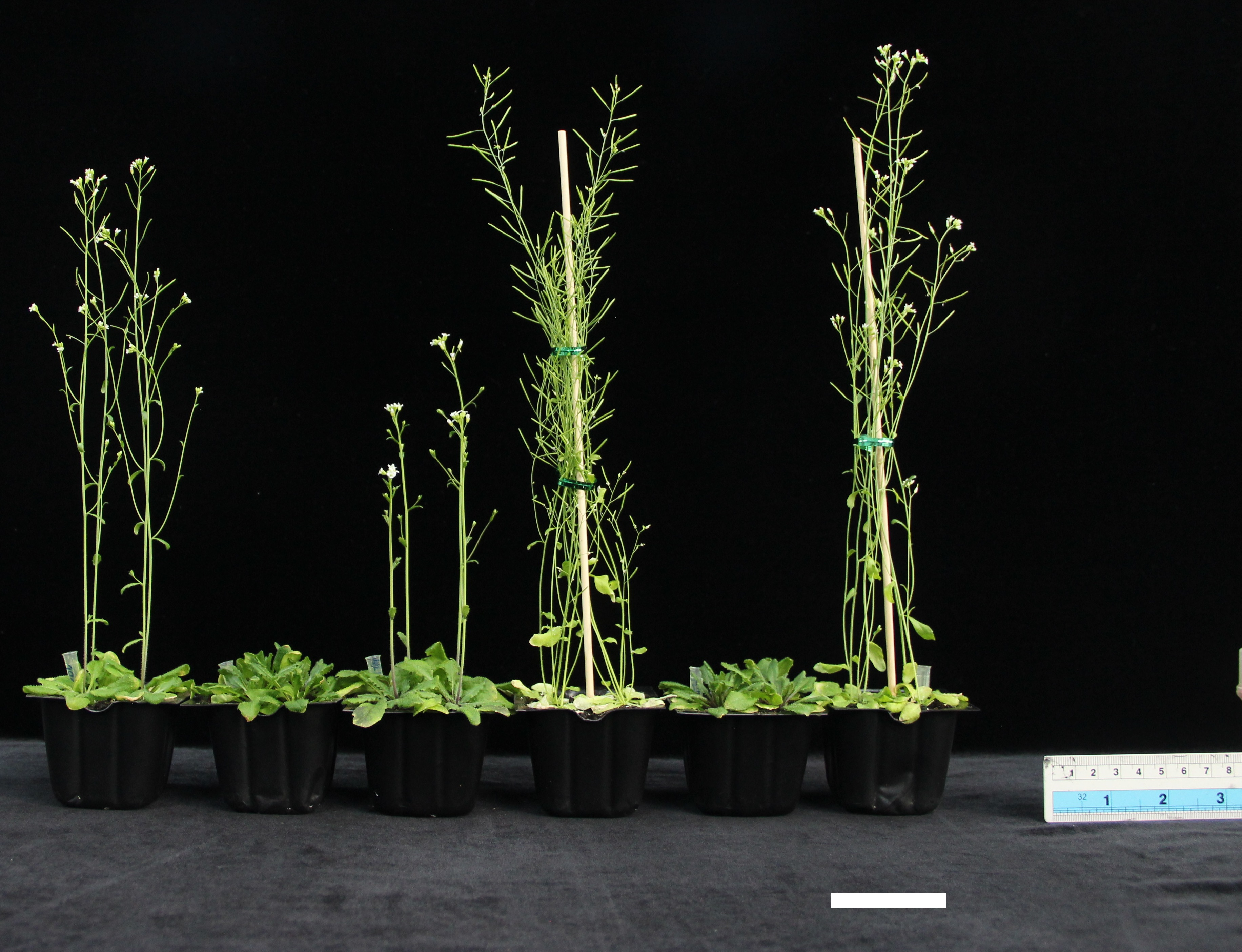

Supplement: Supplementary file 3 — Source data Fig. 1 [file 44318_2024_293_MOESM3_ESM.zip › SD Figure 1/1A/Flowering phenotype of representative plant lines.tif]

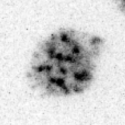

Supplement: Supplementary file 3 — Source data Fig. 1 [file 44318_2024_293_MOESM3_ESM.zip › SD Figure 1/1C/mcherry-CO signal in mcherry-CO ycT root cell nucleus.tif]

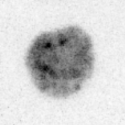

Supplement: Supplementary file 3 — Source data Fig. 1 [file 44318_2024_293_MOESM3_ESM.zip › SD Figure 1/1D/mcherry-CO ycT YC9-GFP light 2h_CO_RGB.tif]

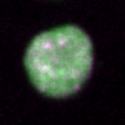

Supplement: Supplementary file 3 — Source data Fig. 1 [file 44318_2024_293_MOESM3_ESM.zip › SD Figure 1/1D/mcherry-CO ycT YC9-GFP light 2h_merge_RGB.tif]

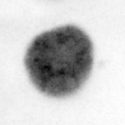

Supplement: Supplementary file 3 — Source data Fig. 1 [file 44318_2024_293_MOESM3_ESM.zip › SD Figure 1/1D/mcherry-CO ycT YC9-GFP light 2h_YC9_RGB.tif]

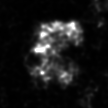

Supplement: Supplementary file 3 — Source data Fig. 1 [file 44318_2024_293_MOESM3_ESM.zip › SD Figure 1/1F/mCherry-CO YC9-GFP ycT_FRAP_Postbleach 0s_decon.tif]

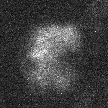

Supplement: Supplementary file 3 — Source data Fig. 1 [file 44318_2024_293_MOESM3_ESM.zip › SD Figure 1/1F/mCherry-CO YC9-GFP ycT_FRAP_Postbleach 0s_decon_original.tif]

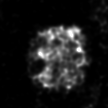

Supplement: Supplementary file 3 — Source data Fig. 1 [file 44318_2024_293_MOESM3_ESM.zip › SD Figure 1/1F/mCherry-CO YC9-GFP ycT_FRAP_Postbleach 12s_decon.tif]

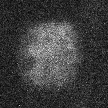

Supplement: Supplementary file 3 — Source data Fig. 1 [file 44318_2024_293_MOESM3_ESM.zip › SD Figure 1/1F/mCherry-CO YC9-GFP ycT_FRAP_Postbleach 12s_decon_original.tif]

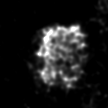

Supplement: Supplementary file 3 — Source data Fig. 1 [file 44318_2024_293_MOESM3_ESM.zip › SD Figure 1/1F/mCherry-CO YC9-GFP ycT_FRAP_Postbleach 2s_decon.tif]

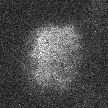

Supplement: Supplementary file 3 — Source data Fig. 1 [file 44318_2024_293_MOESM3_ESM.zip › SD Figure 1/1F/mCherry-CO YC9-GFP ycT_FRAP_Postbleach 2s_decon_original.tif]

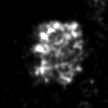

Supplement: Supplementary file 3 — Source data Fig. 1 [file 44318_2024_293_MOESM3_ESM.zip › SD Figure 1/1F/mCherry-CO YC9-GFP ycT_FRAP_Postbleach 4s_decon.tif]

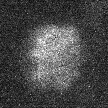

Supplement: Supplementary file 3 — Source data Fig. 1 [file 44318_2024_293_MOESM3_ESM.zip › SD Figure 1/1F/mCherry-CO YC9-GFP ycT_FRAP_Postbleach 4s_decon_original.tif]

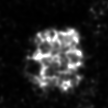

Supplement: Supplementary file 3 — Source data Fig. 1 [file 44318_2024_293_MOESM3_ESM.zip › SD Figure 1/1F/mCherry-CO YC9-GFP ycT_FRAP_Postbleach 60s_decon.tif]

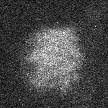

Supplement: Supplementary file 3 — Source data Fig. 1 [file 44318_2024_293_MOESM3_ESM.zip › SD Figure 1/1F/mCherry-CO YC9-GFP ycT_FRAP_Postbleach 60s_decon_original.tif]

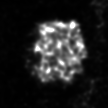

Supplement: Supplementary file 3 — Source data Fig. 1 [file 44318_2024_293_MOESM3_ESM.zip › SD Figure 1/1F/mCherry-CO YC9-GFP ycT_FRAP_Prebleach _decon.tif]

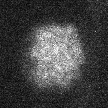

Supplement: Supplementary file 3 — Source data Fig. 1 [file 44318_2024_293_MOESM3_ESM.zip › SD Figure 1/1F/mCherry-CO YC9-GFP ycT_FRAP_Prebleach _original.tif]

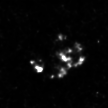

Supplement: Supplementary file 3 — Source data Fig. 1 [file 44318_2024_293_MOESM3_ESM.zip › SD Figure 1/1F/mCherry-CO ycT_FRAP_Postbleach 0s_decon.tif]

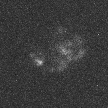

Supplement: Supplementary file 3 — Source data Fig. 1 [file 44318_2024_293_MOESM3_ESM.zip › SD Figure 1/1F/mCherry-CO ycT_FRAP_Postbleach 0s_original.tif]

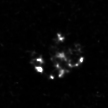

Supplement: Supplementary file 3 — Source data Fig. 1 [file 44318_2024_293_MOESM3_ESM.zip › SD Figure 1/1F/mCherry-CO ycT_FRAP_Postbleach 12s_decon.tif]

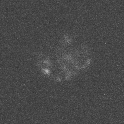

Supplement: Supplementary file 3 — Source data Fig. 1 [file 44318_2024_293_MOESM3_ESM.zip › SD Figure 1/1F/mCherry-CO ycT_FRAP_Postbleach 12s_original.tif]

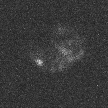

Supplement: Supplementary file 3 — Source data Fig. 1 [file 44318_2024_293_MOESM3_ESM.zip › SD Figure 1/1F/mCherry-CO ycT_FRAP_Postbleach 2s_original.tif]

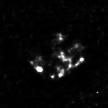

Supplement: Supplementary file 3 — Source data Fig. 1 [file 44318_2024_293_MOESM3_ESM.zip › SD Figure 1/1F/mCherry-CO ycT_FRAP_Postbleach 4s_decon.tif]

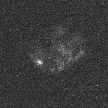

Supplement: Supplementary file 3 — Source data Fig. 1 [file 44318_2024_293_MOESM3_ESM.zip › SD Figure 1/1F/mCherry-CO ycT_FRAP_Postbleach 4s_original.tif]

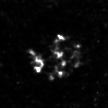

Supplement: Supplementary file 3 — Source data Fig. 1 [file 44318_2024_293_MOESM3_ESM.zip › SD Figure 1/1F/mCherry-CO ycT_FRAP_Postbleach 60s_decon.tif]

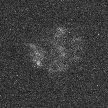

Supplement: Supplementary file 3 — Source data Fig. 1 [file 44318_2024_293_MOESM3_ESM.zip › SD Figure 1/1F/mCherry-CO ycT_FRAP_Postbleach 60s_original.tif]

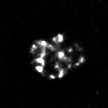

Supplement: Supplementary file 3 — Source data Fig. 1 [file 44318_2024_293_MOESM3_ESM.zip › SD Figure 1/1F/mCherry-CO ycT_FRAP_Prebleach _decon.tif]

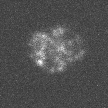

Supplement: Supplementary file 3 — Source data Fig. 1 [file 44318_2024_293_MOESM3_ESM.zip › SD Figure 1/1F/mCherry-CO ycT_FRAP_Prebleach _original.tif]

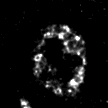

Supplement: Supplementary file 3 — Source data Fig. 1 [file 44318_2024_293_MOESM3_ESM.zip › SD Figure 1/1F/mcherry-CO_WT_FRAP_Postbleach 0s_decon.tif]

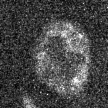

Supplement: Supplementary file 3 — Source data Fig. 1 [file 44318_2024_293_MOESM3_ESM.zip › SD Figure 1/1F/mcherry-CO_WT_FRAP_Postbleach 0s_original.tif]

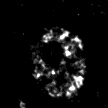

Supplement: Supplementary file 3 — Source data Fig. 1 [file 44318_2024_293_MOESM3_ESM.zip › SD Figure 1/1F/mcherry-CO_WT_FRAP_Postbleach 12s_decon.tif]

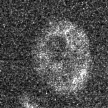

Supplement: Supplementary file 3 — Source data Fig. 1 [file 44318_2024_293_MOESM3_ESM.zip › SD Figure 1/1F/mcherry-CO_WT_FRAP_Postbleach 12s_original.tif]

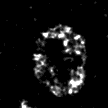

Supplement: Supplementary file 3 — Source data Fig. 1 [file 44318_2024_293_MOESM3_ESM.zip › SD Figure 1/1F/mcherry-CO_WT_FRAP_Postbleach 2s_decon.tif]

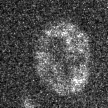

Supplement: Supplementary file 3 — Source data Fig. 1 [file 44318_2024_293_MOESM3_ESM.zip › SD Figure 1/1F/mcherry-CO_WT_FRAP_Postbleach 2s_original.tif]

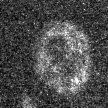

Supplement: Supplementary file 3 — Source data Fig. 1 [file 44318_2024_293_MOESM3_ESM.zip › SD Figure 1/1F/mcherry-CO_WT_FRAP_Postbleach 4s_original.tif]

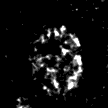

Supplement: Supplementary file 3 — Source data Fig. 1 [file 44318_2024_293_MOESM3_ESM.zip › SD Figure 1/1F/mcherry-CO_WT_FRAP_Postbleach 60s_decon.tif]

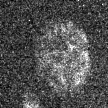

Supplement: Supplementary file 3 — Source data Fig. 1 [file 44318_2024_293_MOESM3_ESM.zip › SD Figure 1/1F/mcherry-CO_WT_FRAP_Postbleach 60s_original.tif]

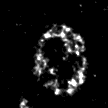

Supplement: Supplementary file 3 — Source data Fig. 1 [file 44318_2024_293_MOESM3_ESM.zip › SD Figure 1/1F/mcherry-CO_WT_FRAP_Postbleach 6s_decon.tif]

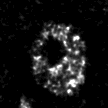

Supplement: Supplementary file 3 — Source data Fig. 1 [file 44318_2024_293_MOESM3_ESM.zip › SD Figure 1/1F/mcherry-CO_WT_FRAP_Prebleach_decon.tif]

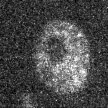

Supplement: Supplementary file 3 — Source data Fig. 1 [file 44318_2024_293_MOESM3_ESM.zip › SD Figure 1/1F/mcherry-CO_WT_FRAP_Prebleach_original.tif]

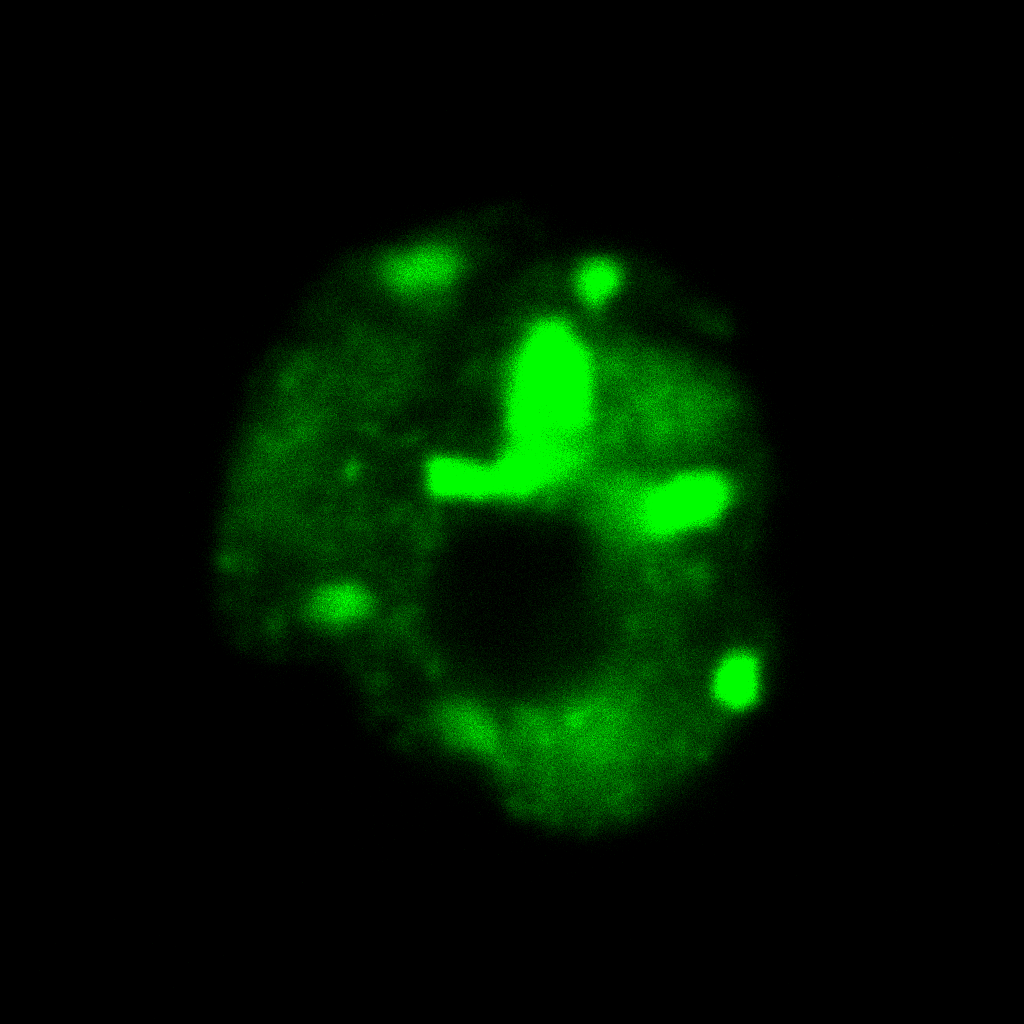

Supplement: Supplementary file 4 — Source data Fig. 2 [file 44318_2024_293_MOESM4_ESM.zip › SD Figure 2/2B/GFP-CO assembly status (amorphous aggregates).tif]

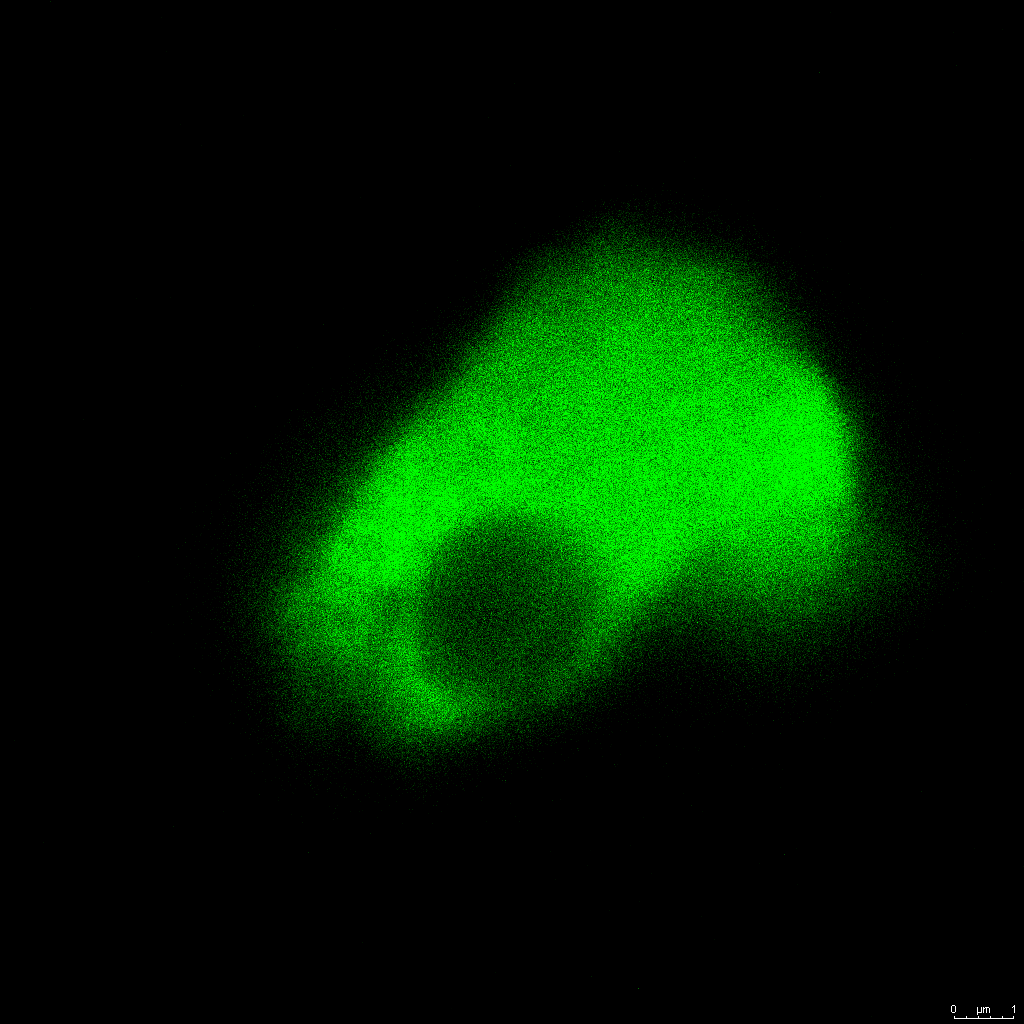

Supplement: Supplementary file 4 — Source data Fig. 2 [file 44318_2024_293_MOESM4_ESM.zip › SD Figure 2/2B/GFP-CO assembly status (diffuse).tif]

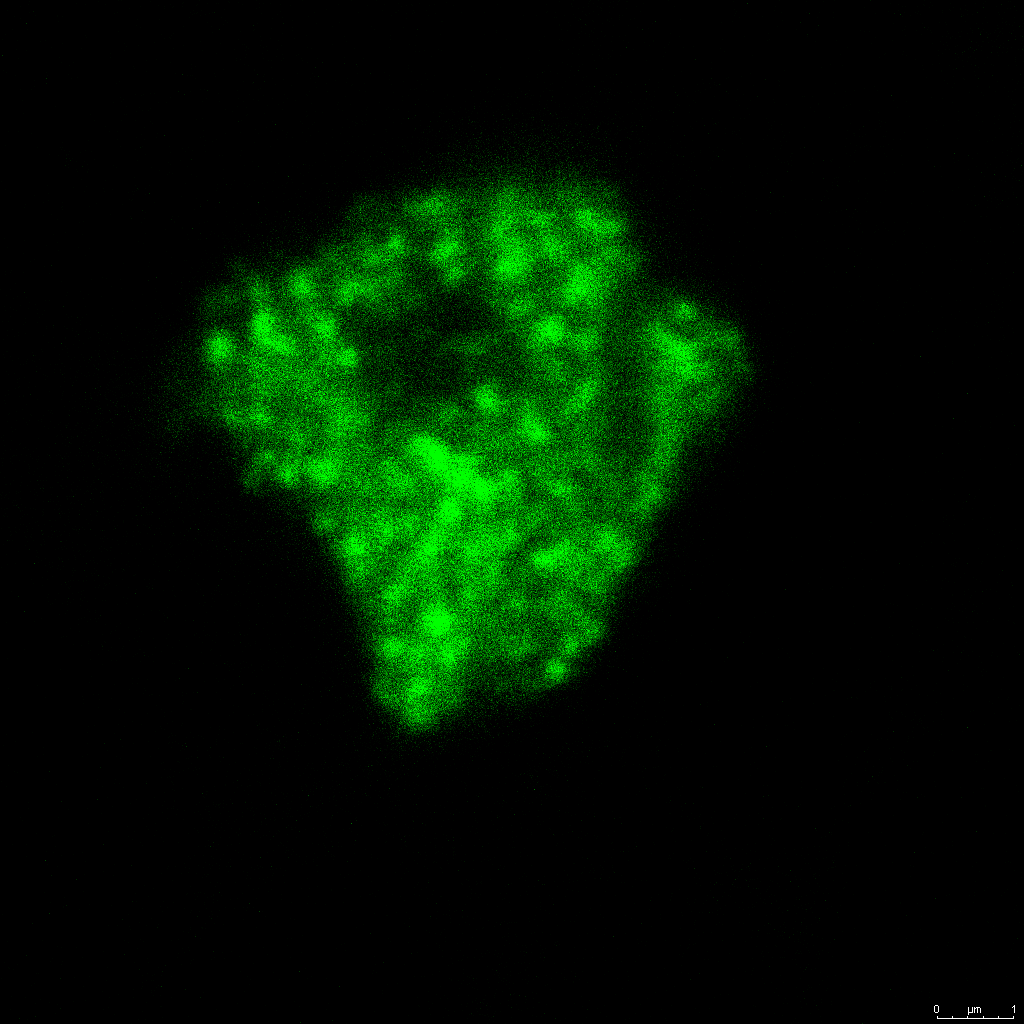

Supplement: Supplementary file 4 — Source data Fig. 2 [file 44318_2024_293_MOESM4_ESM.zip › SD Figure 2/2B/GFP-CO assembly status (spherical condensation).tif]

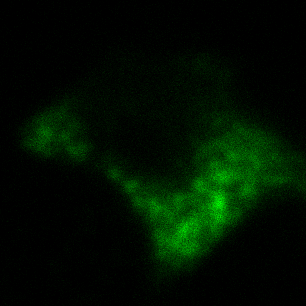

Supplement: Supplementary file 4 — Source data Fig. 2 [file 44318_2024_293_MOESM4_ESM.zip › SD Figure 2/2C/FRAP of GFP-CO spherical condensates (Liquid) in CO expression alone/GFP-CO_FRAP_Postbleach 0s.tif]

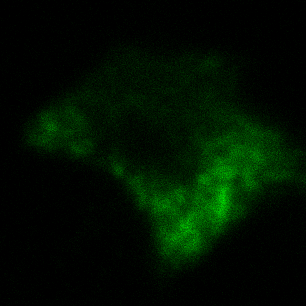

Supplement: Supplementary file 4 — Source data Fig. 2 [file 44318_2024_293_MOESM4_ESM.zip › SD Figure 2/2C/FRAP of GFP-CO spherical condensates (Liquid) in CO expression alone/GFP-CO_FRAP_Postbleach 10s.tif]

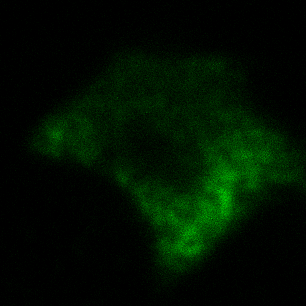

Supplement: Supplementary file 4 — Source data Fig. 2 [file 44318_2024_293_MOESM4_ESM.zip › SD Figure 2/2C/FRAP of GFP-CO spherical condensates (Liquid) in CO expression alone/GFP-CO_FRAP_Postbleach 20s.tif]

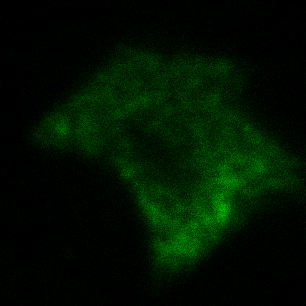

Supplement: Supplementary file 4 — Source data Fig. 2 [file 44318_2024_293_MOESM4_ESM.zip › SD Figure 2/2C/FRAP of GFP-CO spherical condensates (Liquid) in CO expression alone/GFP-CO_FRAP_Postbleach 30s.tif]

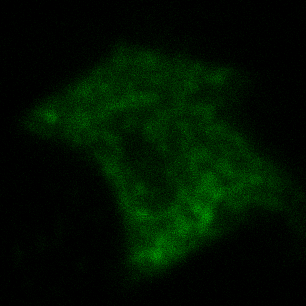

Supplement: Supplementary file 4 — Source data Fig. 2 [file 44318_2024_293_MOESM4_ESM.zip › SD Figure 2/2C/FRAP of GFP-CO spherical condensates (Liquid) in CO expression alone/GFP-CO_FRAP_Postbleach 60s.tif]

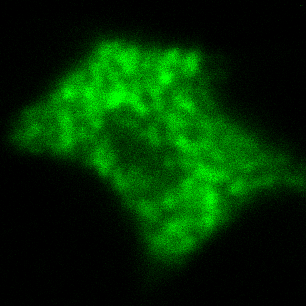

Supplement: Supplementary file 4 — Source data Fig. 2 [file 44318_2024_293_MOESM4_ESM.zip › SD Figure 2/2C/FRAP of GFP-CO spherical condensates (Liquid) in CO expression alone/GFP-CO_FRAP_Prebleach 0s.tif]

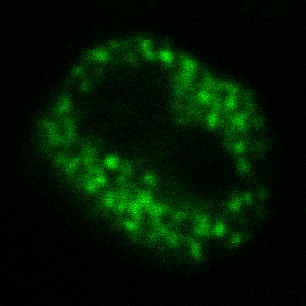

Supplement: Supplementary file 4 — Source data Fig. 2 [file 44318_2024_293_MOESM4_ESM.zip › SD Figure 2/2C/FRAP of GFP-CO spherical condensates (Slow-diffusive) in CO expression alone/GFP-CO_FRAP_Postbleach 0s.tif]

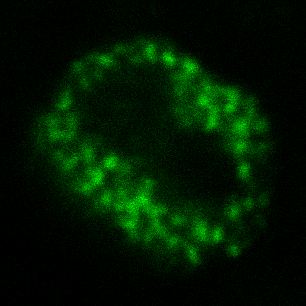

Supplement: Supplementary file 4 — Source data Fig. 2 [file 44318_2024_293_MOESM4_ESM.zip › SD Figure 2/2C/FRAP of GFP-CO spherical condensates (Slow-diffusive) in CO expression alone/GFP-CO_FRAP_Postbleach 10s.tif]

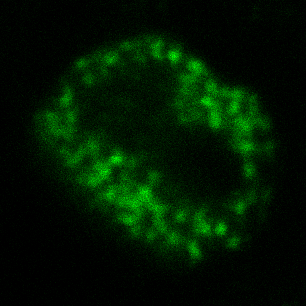

Supplement: Supplementary file 4 — Source data Fig. 2 [file 44318_2024_293_MOESM4_ESM.zip › SD Figure 2/2C/FRAP of GFP-CO spherical condensates (Slow-diffusive) in CO expression alone/GFP-CO_FRAP_Postbleach 20s.tif]

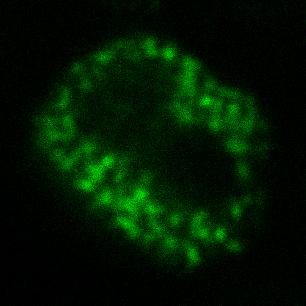

Supplement: Supplementary file 4 — Source data Fig. 2 [file 44318_2024_293_MOESM4_ESM.zip › SD Figure 2/2C/FRAP of GFP-CO spherical condensates (Slow-diffusive) in CO expression alone/GFP-CO_FRAP_Postbleach 30s.tif]

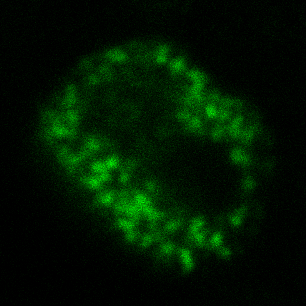

Supplement: Supplementary file 4 — Source data Fig. 2 [file 44318_2024_293_MOESM4_ESM.zip › SD Figure 2/2C/FRAP of GFP-CO spherical condensates (Slow-diffusive) in CO expression alone/GFP-CO_FRAP_Postbleach 60s.tif]

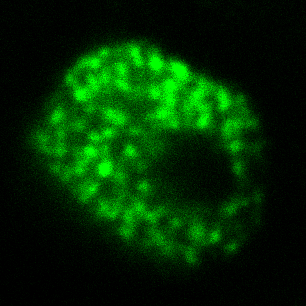

Supplement: Supplementary file 4 — Source data Fig. 2 [file 44318_2024_293_MOESM4_ESM.zip › SD Figure 2/2C/FRAP of GFP-CO spherical condensates (Slow-diffusive) in CO expression alone/GFP-CO_FRAP_Prebleach 0s.tif]

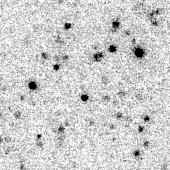

Supplement: Supplementary file 5 — Source data Fig. 3 [file 44318_2024_293_MOESM5_ESM.zip › SD Figure 3/3B/C1-mcherry-CO.tif]

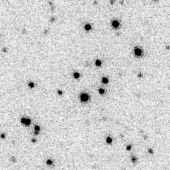

Supplement: Supplementary file 5 — Source data Fig. 3 [file 44318_2024_293_MOESM5_ESM.zip › SD Figure 3/3B/C2-YC9-GFP.tif]

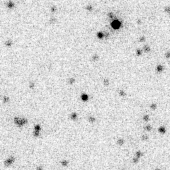

Supplement: Supplementary file 5 — Source data Fig. 3 [file 44318_2024_293_MOESM5_ESM.zip › SD Figure 3/3B/C3-YB2_Alexa 647.tif]

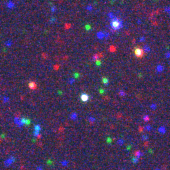

Supplement: Supplementary file 5 — Source data Fig. 3 [file 44318_2024_293_MOESM5_ESM.zip › SD Figure 3/3B/merge.tif.tif]

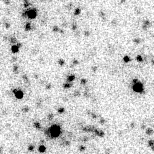

Supplement: Supplementary file 5 — Source data Fig. 3 [file 44318_2024_293_MOESM5_ESM.zip › SD Figure 3/3C/10 nM mCherry-CO alone.tif]

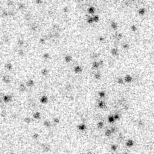

Supplement: Supplementary file 5 — Source data Fig. 3 [file 44318_2024_293_MOESM5_ESM.zip › SD Figure 3/3C/10 nM mCherry-CO+10 nM YC9+100nM YB2.tif]

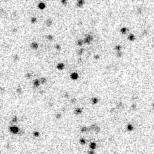

Supplement: Supplementary file 5 — Source data Fig. 3 [file 44318_2024_293_MOESM5_ESM.zip › SD Figure 3/3C/10 nM mCherry-CO+10 nM YC9+10nM YB2.tif]

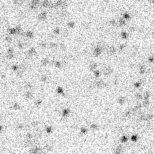

Supplement: Supplementary file 5 — Source data Fig. 3 [file 44318_2024_293_MOESM5_ESM.zip › SD Figure 3/3C/10 nM mCherry-CO+10 nM YC9+600nM YB2.tif]

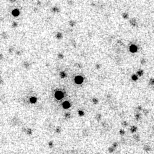

Supplement: Supplementary file 5 — Source data Fig. 3 [file 44318_2024_293_MOESM5_ESM.zip › SD Figure 3/3C/10 nM mCherry-CO+10 nM YC9.tif]

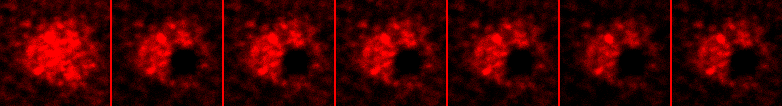

Supplement: Supplementary file 5 — Source data Fig. 3 [file 44318_2024_293_MOESM5_ESM.zip › SD Figure 3/3E/mCherry-CO + YC9-GFP + YB2 + DNA.tif]

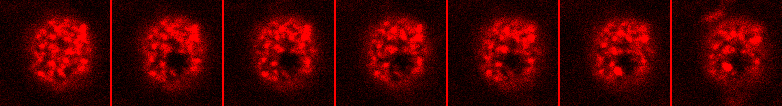

Supplement: Supplementary file 5 — Source data Fig. 3 [file 44318_2024_293_MOESM5_ESM.zip › SD Figure 3/3E/mCherry-CO + YC9-GFP + YB2 + FT.tif]

## Slide 1
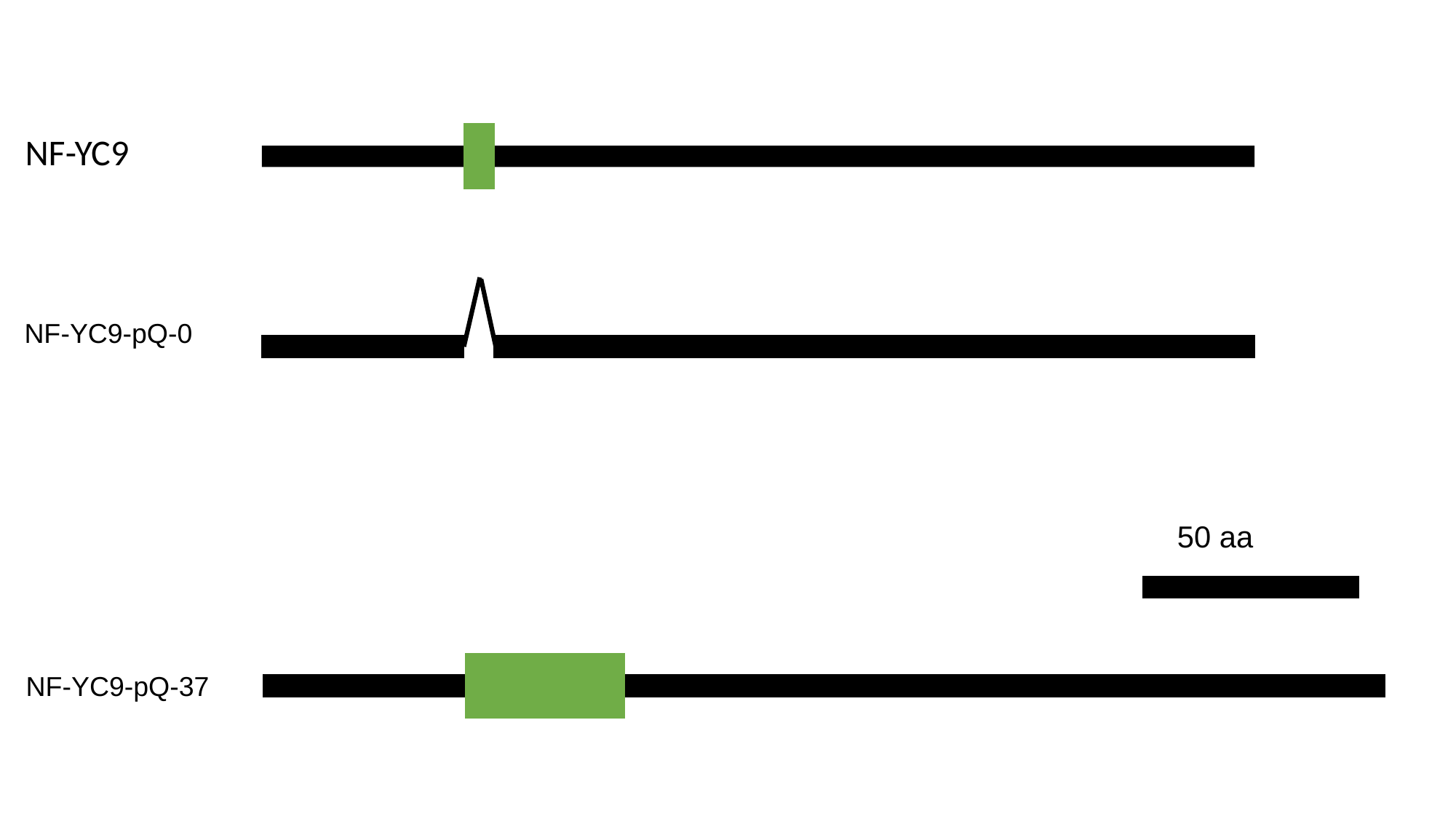

NF-YC9
NF-YC9-pQ-0
50 aa
NF-YC9-pQ-37

Supplement: Supplementary file 6 — Source data Fig. 4 [file 44318_2024_293_MOESM6_ESM.zip › SD Figure 4/4A/Schematic of NF-YC9 and NF-YC9-polyQ mutant constructs.pptx]

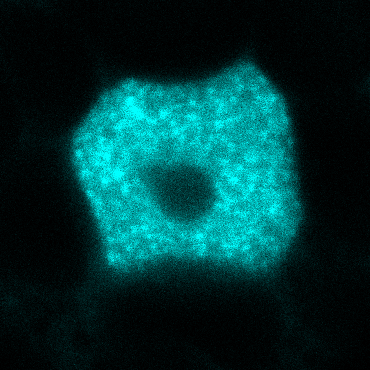

Supplement: Supplementary file 6 — Source data Fig. 4 [file 44318_2024_293_MOESM6_ESM.zip › SD Figure 4/4B/GFP-CO+YC9-0Q-mCherry+YB2-BFP_BFP channel.tif]

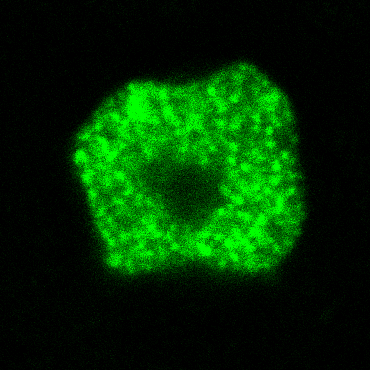

Supplement: Supplementary file 6 — Source data Fig. 4 [file 44318_2024_293_MOESM6_ESM.zip › SD Figure 4/4B/GFP-CO+YC9-0Q-mCherry+YB2-BFP_GFP channel.tif]

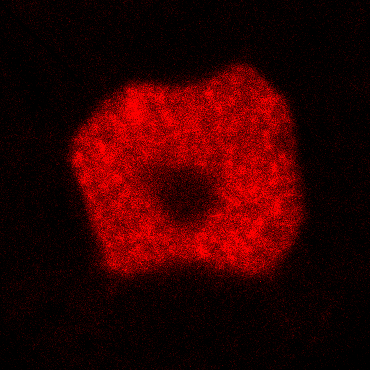

Supplement: Supplementary file 6 — Source data Fig. 4 [file 44318_2024_293_MOESM6_ESM.zip › SD Figure 4/4B/GFP-CO+YC9-0Q-mCherry+YB2-BFP_mCherry channel.tif]

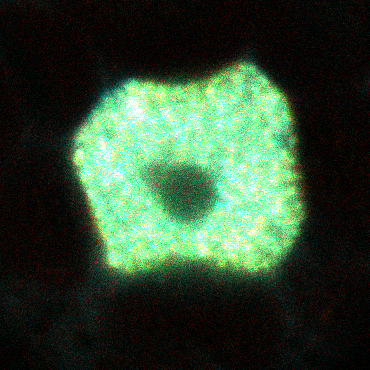

Supplement: Supplementary file 6 — Source data Fig. 4 [file 44318_2024_293_MOESM6_ESM.zip › SD Figure 4/4B/GFP-CO+YC9-0Q-mCherry+YB2-BFP_Merge.tif]

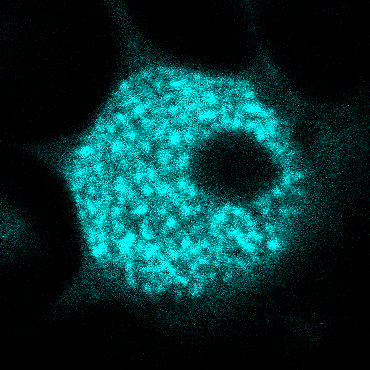

Supplement: Supplementary file 6 — Source data Fig. 4 [file 44318_2024_293_MOESM6_ESM.zip › SD Figure 4/4B/GFP-CO+YC9-37Q-mCherry+YB2-BFP_BFP channel.tif]

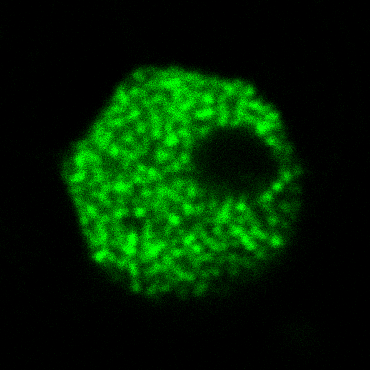

Supplement: Supplementary file 6 — Source data Fig. 4 [file 44318_2024_293_MOESM6_ESM.zip › SD Figure 4/4B/GFP-CO+YC9-37Q-mCherry+YB2-BFP_GFP channel.tif]

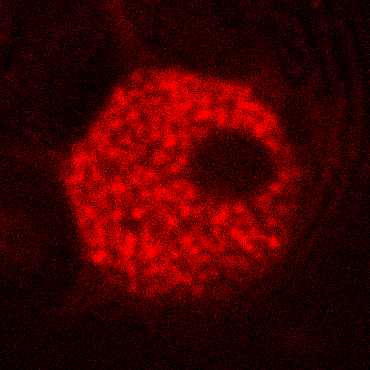

Supplement: Supplementary file 6 — Source data Fig. 4 [file 44318_2024_293_MOESM6_ESM.zip › SD Figure 4/4B/GFP-CO+YC9-37Q-mCherry+YB2-BFP_mCherry channel.tif]

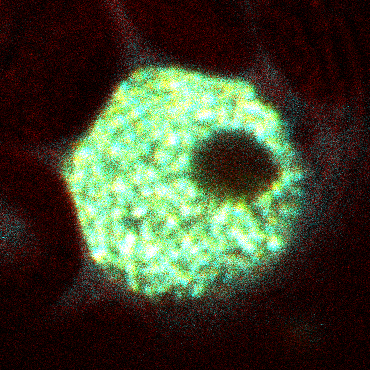

Supplement: Supplementary file 6 — Source data Fig. 4 [file 44318_2024_293_MOESM6_ESM.zip › SD Figure 4/4B/GFP-CO+YC9-37Q-mCherry+YB2-BFP_Merge.tif]

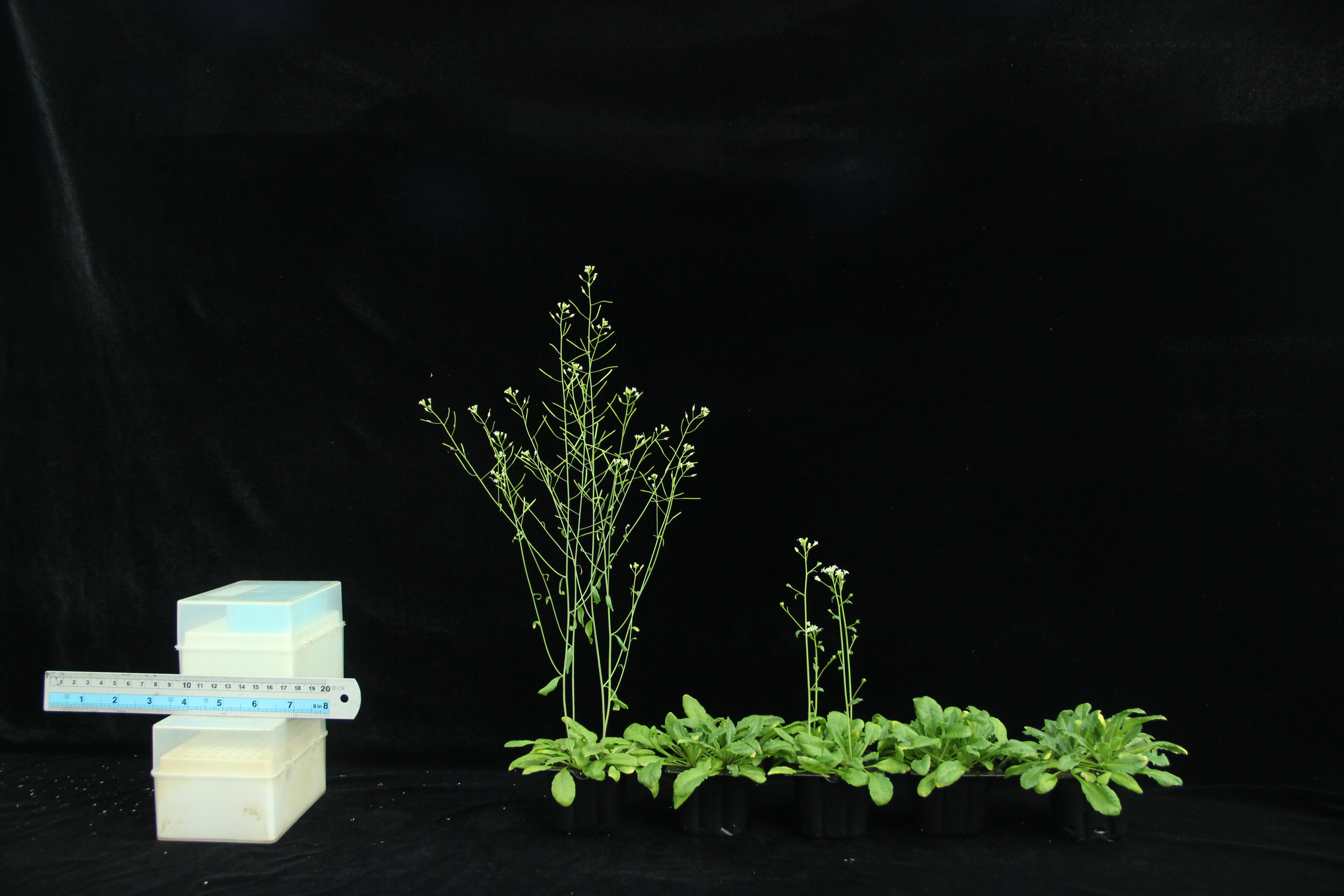

Supplement: Supplementary file 6 — Source data Fig. 4 [file 44318_2024_293_MOESM6_ESM.zip › SD Figure 4/4G/Flowering phenotype of representative plant lines.JPG]

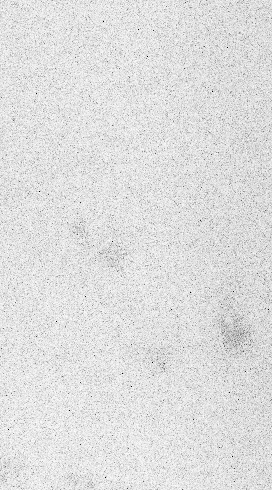

Supplement: Supplementary file 7 — EV Figures Source Data [file 44318_2024_293_MOESM7_ESM.zip › SD Figure EV1/EV1 A/mcherry-CO ycT light 0h_RGB.tif]

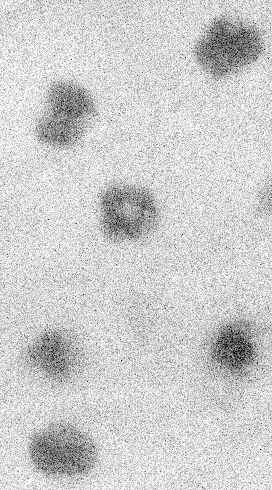

Supplement: Supplementary file 7 — EV Figures Source Data [file 44318_2024_293_MOESM7_ESM.zip › SD Figure EV1/EV1 A/mcherry-CO ycT light 1h_RGB.tif]

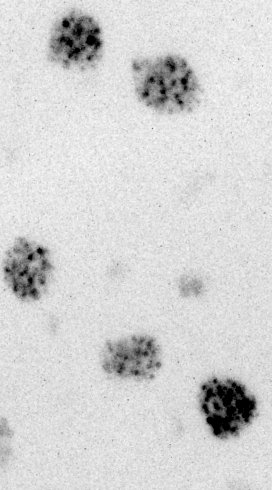

Supplement: Supplementary file 7 — EV Figures Source Data [file 44318_2024_293_MOESM7_ESM.zip › SD Figure EV1/EV1 A/mcherry-CO ycT light 2h_RGB.tif]

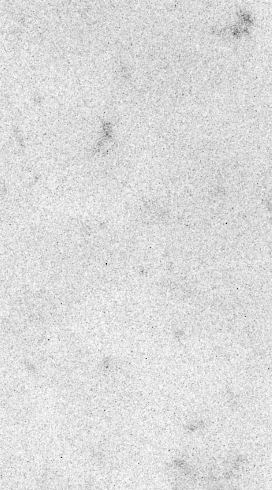

Supplement: Supplementary file 7 — EV Figures Source Data [file 44318_2024_293_MOESM7_ESM.zip › SD Figure EV1/EV1 A/mcherry-CO ycT YC9-GFP light 0h_RGB.tif]

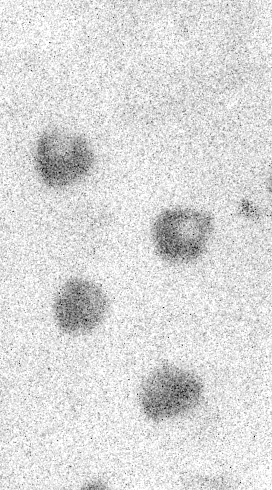

Supplement: Supplementary file 7 — EV Figures Source Data [file 44318_2024_293_MOESM7_ESM.zip › SD Figure EV1/EV1 A/mcherry-CO ycT YC9-GFP light 1h_RGB.tif]

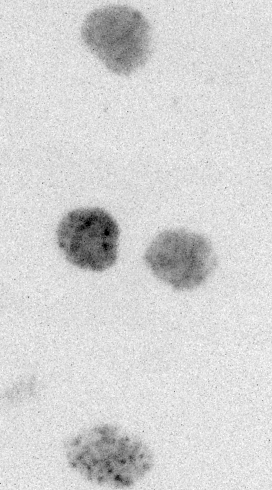

Supplement: Supplementary file 7 — EV Figures Source Data [file 44318_2024_293_MOESM7_ESM.zip › SD Figure EV1/EV1 A/mcherry-CO ycT YC9-GFP light 2h_RGB.tif]

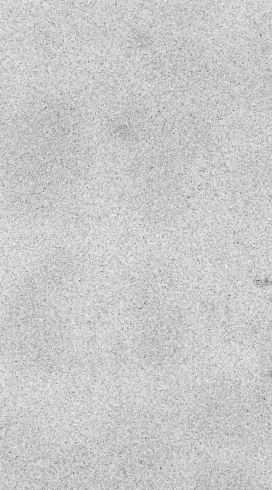

Supplement: Supplementary file 7 — EV Figures Source Data [file 44318_2024_293_MOESM7_ESM.zip › SD Figure EV1/EV1 A/mcherry-CO_light 0h_RGB.tif]

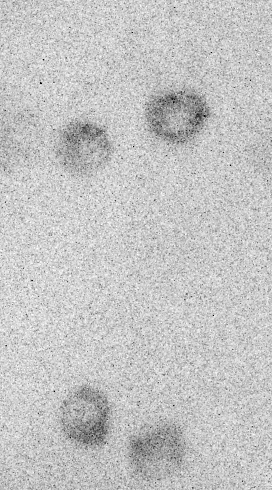

Supplement: Supplementary file 7 — EV Figures Source Data [file 44318_2024_293_MOESM7_ESM.zip › SD Figure EV1/EV1 A/mcherry-CO_light 1h_RGB.tif]

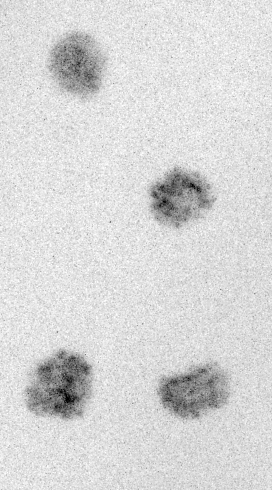

Supplement: Supplementary file 7 — EV Figures Source Data [file 44318_2024_293_MOESM7_ESM.zip › SD Figure EV1/EV1 A/mcherry-CO_light 2h_RGB.tif]

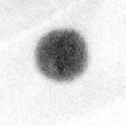

Supplement: Supplementary file 7 — EV Figures Source Data [file 44318_2024_293_MOESM7_ESM.zip › SD Figure EV1/EV1 C/YC9-GFP ycT.tif]

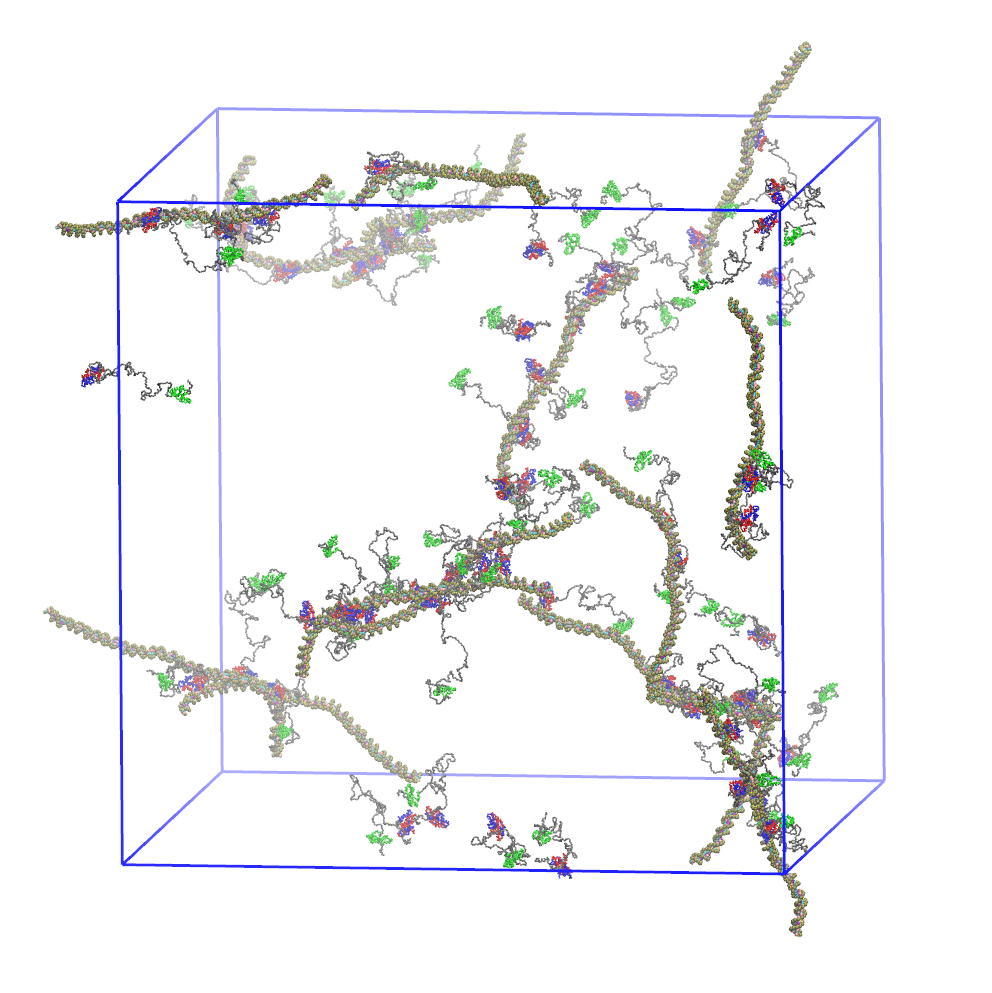

Supplement: Supplementary file 7 — EV Figures Source Data [file 44318_2024_293_MOESM7_ESM.zip › SD Figure EV1/EV1 D/monomer_DNA_equ.png]

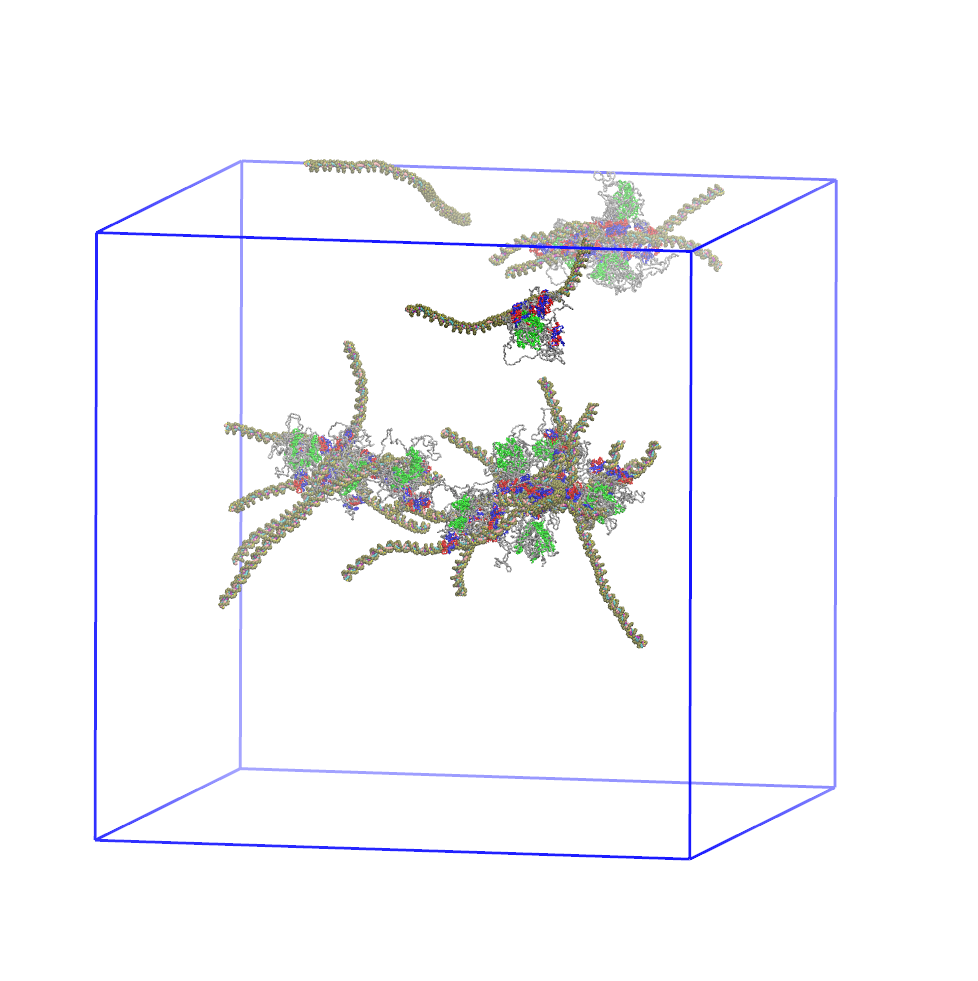

Supplement: Supplementary file 7 — EV Figures Source Data [file 44318_2024_293_MOESM7_ESM.zip › SD Figure EV1/EV1 D/pentamer_DNA_equ.png]

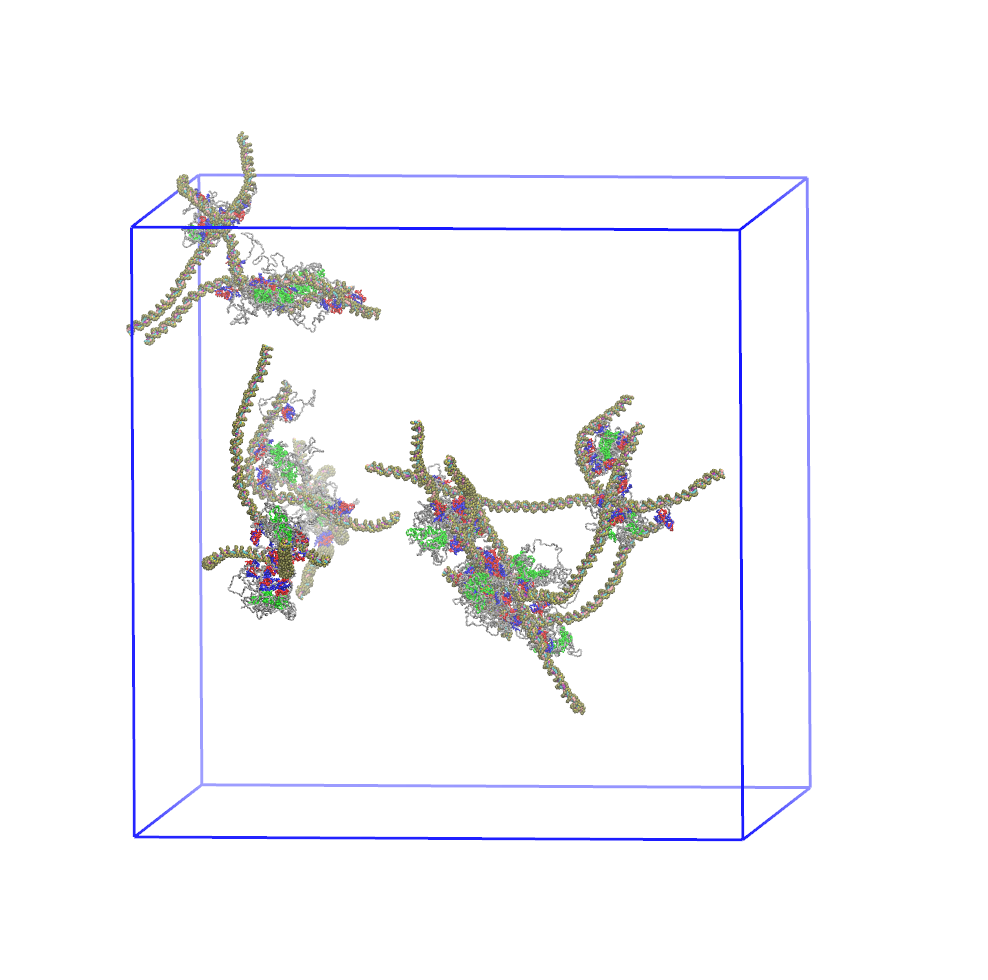

Supplement: Supplementary file 7 — EV Figures Source Data [file 44318_2024_293_MOESM7_ESM.zip › SD Figure EV1/EV1 D/tetramer_DNA_equ.png]

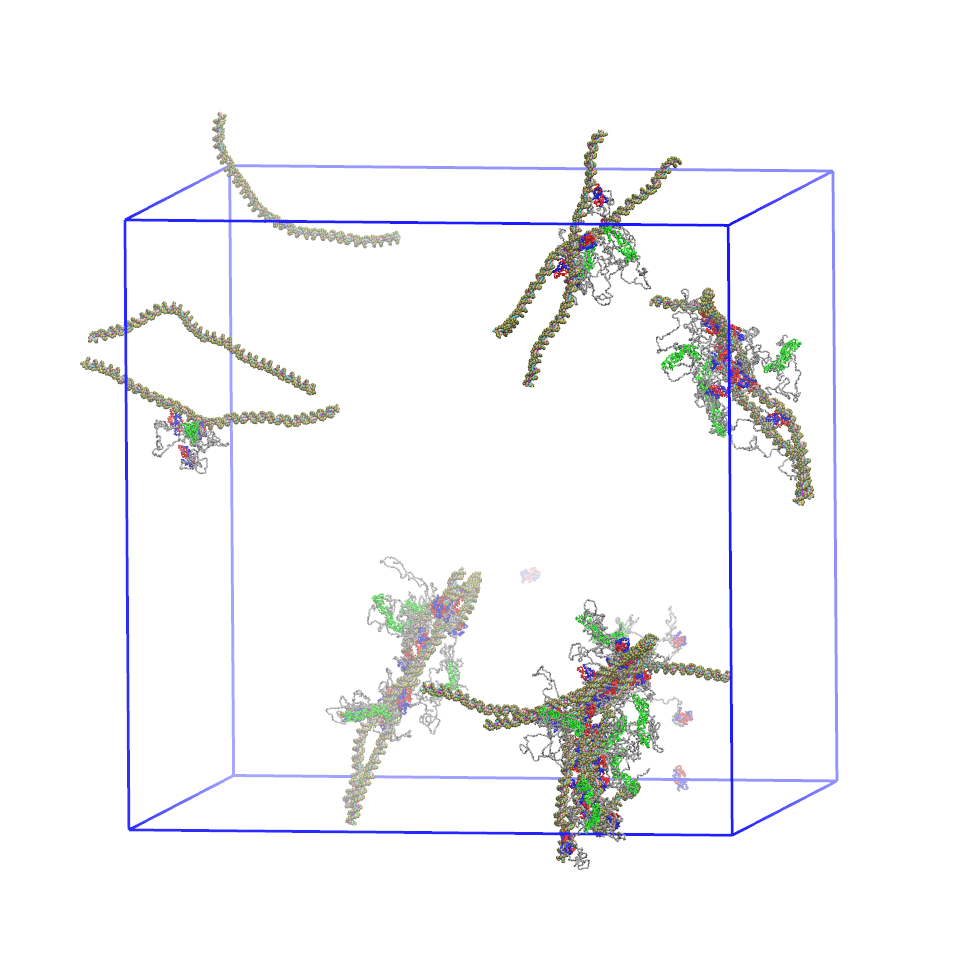

Supplement: Supplementary file 7 — EV Figures Source Data [file 44318_2024_293_MOESM7_ESM.zip › SD Figure EV1/EV1 D/trimer_DNA_equ.png]

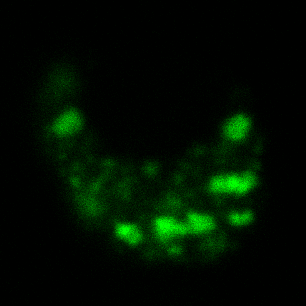

Supplement: Supplementary file 7 — EV Figures Source Data [file 44318_2024_293_MOESM7_ESM.zip › SD Figure EV2/EV2 C/FRAP of GFP-CO irreversible aggregate in CO expression alone/GFP-CO_FRAP_Postbleach 0s.tif]

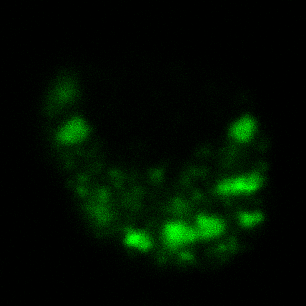

Supplement: Supplementary file 7 — EV Figures Source Data [file 44318_2024_293_MOESM7_ESM.zip › SD Figure EV2/EV2 C/FRAP of GFP-CO irreversible aggregate in CO expression alone/GFP-CO_FRAP_Postbleach 10s.tif]

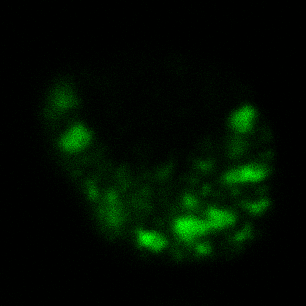

Supplement: Supplementary file 7 — EV Figures Source Data [file 44318_2024_293_MOESM7_ESM.zip › SD Figure EV2/EV2 C/FRAP of GFP-CO irreversible aggregate in CO expression alone/GFP-CO_FRAP_Postbleach 20s.tif]

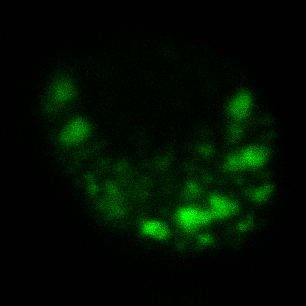

Supplement: Supplementary file 7 — EV Figures Source Data [file 44318_2024_293_MOESM7_ESM.zip › SD Figure EV2/EV2 C/FRAP of GFP-CO irreversible aggregate in CO expression alone/GFP-CO_FRAP_Postbleach 30s.tif]

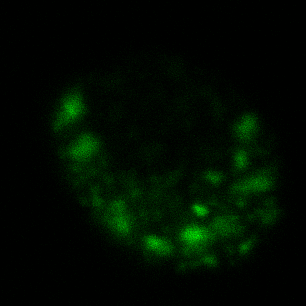

Supplement: Supplementary file 7 — EV Figures Source Data [file 44318_2024_293_MOESM7_ESM.zip › SD Figure EV2/EV2 C/FRAP of GFP-CO irreversible aggregate in CO expression alone/GFP-CO_FRAP_Postbleach 60s.tif]

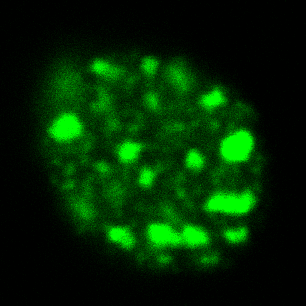

Supplement: Supplementary file 7 — EV Figures Source Data [file 44318_2024_293_MOESM7_ESM.zip › SD Figure EV2/EV2 C/FRAP of GFP-CO irreversible aggregate in CO expression alone/GFP-CO_FRAP_Prebleach 0s.tif]

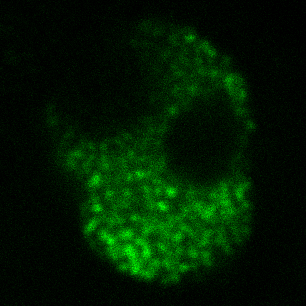

Supplement: Supplementary file 7 — EV Figures Source Data [file 44318_2024_293_MOESM7_ESM.zip › SD Figure EV2/EV2 C/FRAP of GFP-CO spherical condensates (Liquid) in CO+YC9+YB2 co-expression/GFP-CO_FRAP_Postbleach 0s.tif]

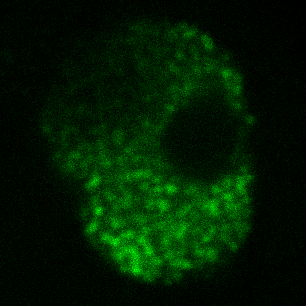

Supplement: Supplementary file 7 — EV Figures Source Data [file 44318_2024_293_MOESM7_ESM.zip › SD Figure EV2/EV2 C/FRAP of GFP-CO spherical condensates (Liquid) in CO+YC9+YB2 co-expression/GFP-CO_FRAP_Postbleach 10s.tif]

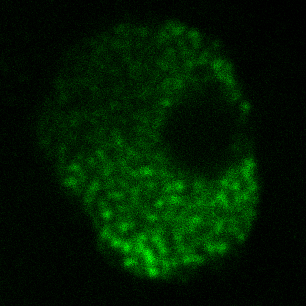

Supplement: Supplementary file 7 — EV Figures Source Data [file 44318_2024_293_MOESM7_ESM.zip › SD Figure EV2/EV2 C/FRAP of GFP-CO spherical condensates (Liquid) in CO+YC9+YB2 co-expression/GFP-CO_FRAP_Postbleach 20s.tif]

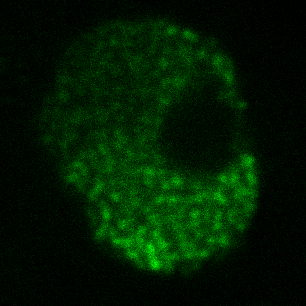

Supplement: Supplementary file 7 — EV Figures Source Data [file 44318_2024_293_MOESM7_ESM.zip › SD Figure EV2/EV2 C/FRAP of GFP-CO spherical condensates (Liquid) in CO+YC9+YB2 co-expression/GFP-CO_FRAP_Postbleach 30s.tif]
