# Supplementary figures and images for: Molecular condensation of the CO/NF-YB/NF-YC/FT complex gates floral transition in Arabidopsis (part 3 of 3)
Source: EMBO J. 2024 Nov 20;44(1):225–50. doi: 10.1038/s44318-024-00293-0 (PMC11696179; doi:10.1038/s44318-024-00293-0)

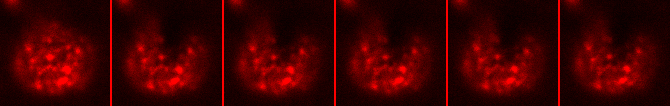

Supplement: Supplementary file 7 — EV Figures Source Data [file 44318_2024_293_MOESM7_ESM.zip › SD Figure EV3/EV3 H/mCherry-CO+YC9-GFP+YB2 (mCherry-CO images).tif]

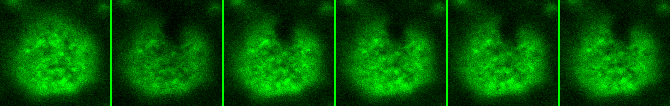

Supplement: Supplementary file 7 — EV Figures Source Data [file 44318_2024_293_MOESM7_ESM.zip › SD Figure EV3/EV3 H/mCherry-CO+YC9-GFP+YB2 (YC9-GFP images).tif]

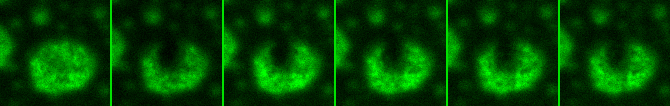

Supplement: Supplementary file 7 — EV Figures Source Data [file 44318_2024_293_MOESM7_ESM.zip › SD Figure EV3/EV3 H/YC9-GFP+YB2 (YC9-GFP images).tif]

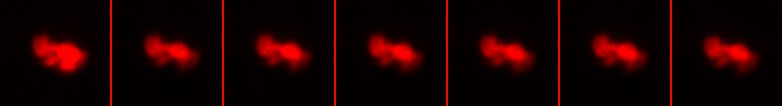

Supplement: Supplementary file 7 — EV Figures Source Data [file 44318_2024_293_MOESM7_ESM.zip › SD Figure EV3/EV3 K/mCherry-CO+FT.tif]

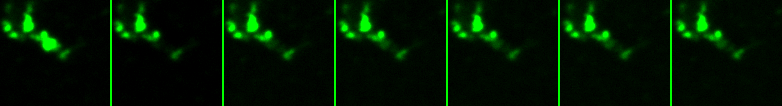

Supplement: Supplementary file 7 — EV Figures Source Data [file 44318_2024_293_MOESM7_ESM.zip › SD Figure EV3/EV3 K/YC9-GFP+FT.tif]

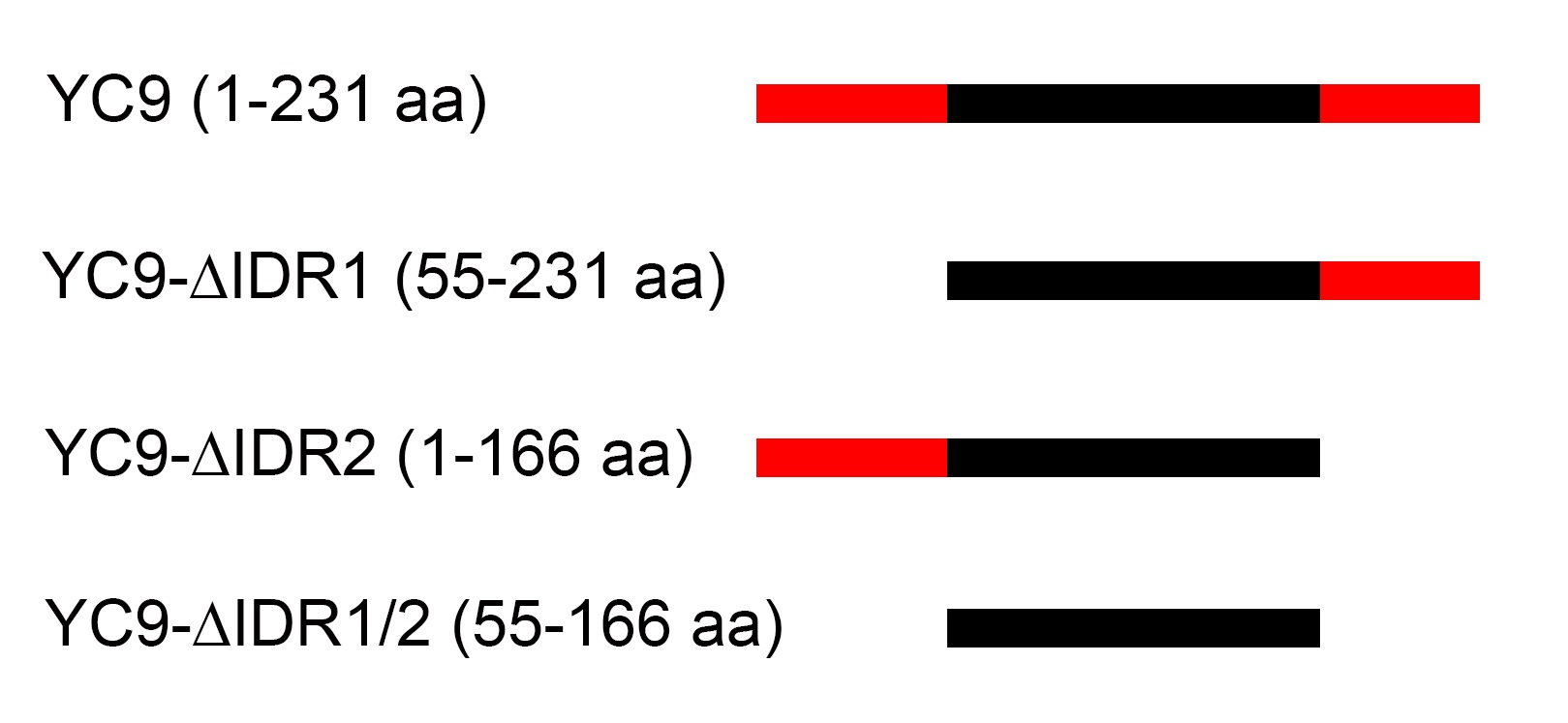

Supplement: Supplementary file 7 — EV Figures Source Data [file 44318_2024_293_MOESM7_ESM.zip › SD Figure EV4/EV4 A/Schematic of NF-YC9 and NF-YC9-∆IDR mutants.tif]

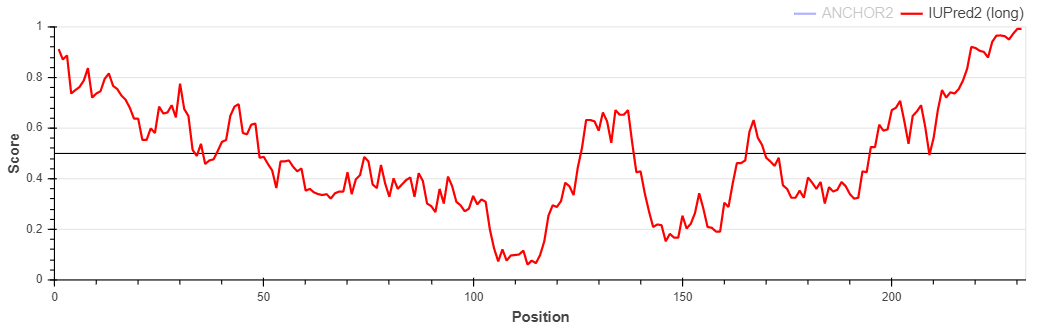

Supplement: Supplementary file 7 — EV Figures Source Data [file 44318_2024_293_MOESM7_ESM.zip › SD Figure EV4/EV4 A/Sequence analysis of NF-YC9 containing two IDR domains.tif]

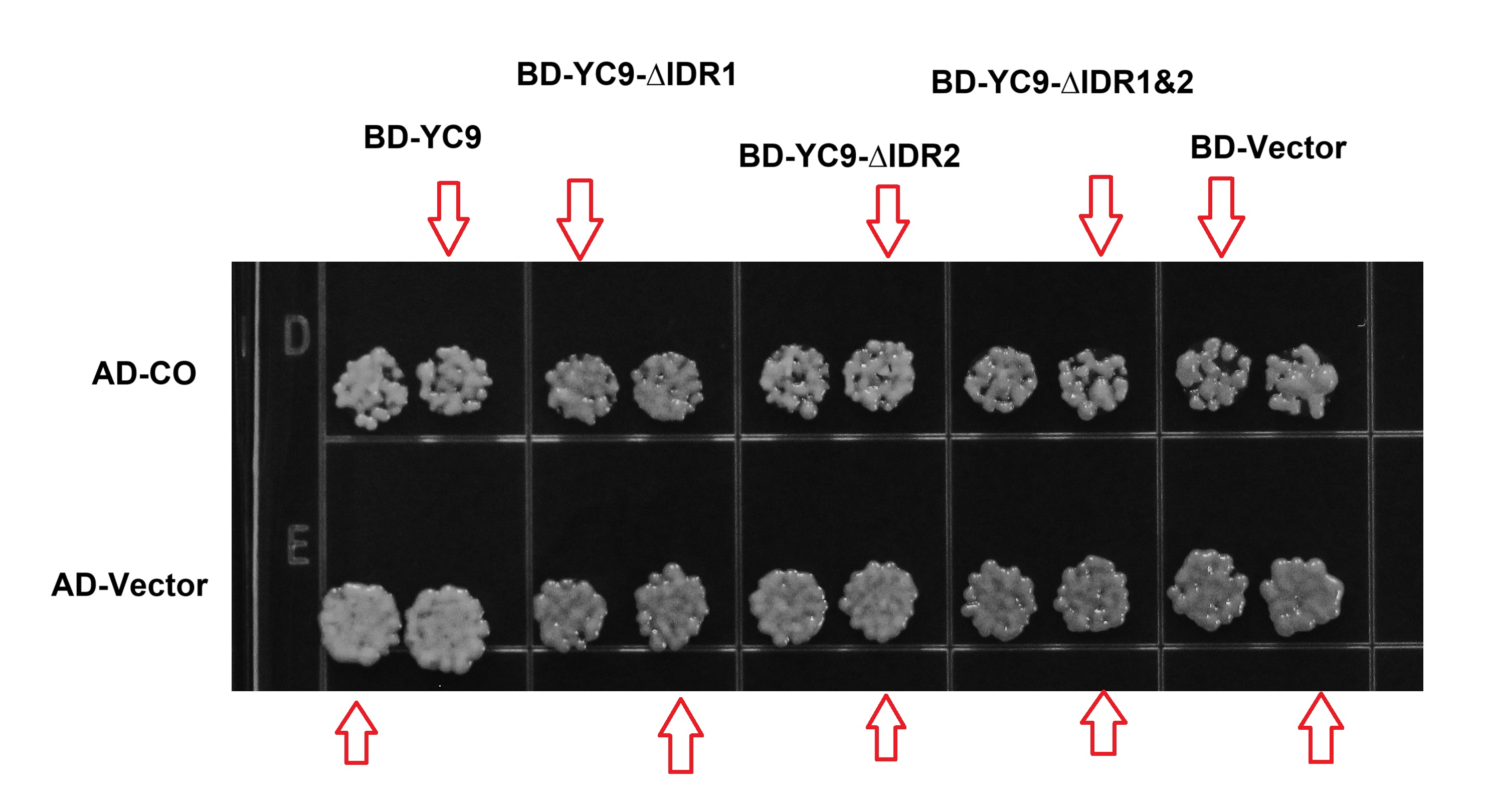

Supplement: Supplementary file 7 — EV Figures Source Data [file 44318_2024_293_MOESM7_ESM.zip › SD Figure EV4/EV4 B/Yeast two-hybrid assays of CO and NF-YC9 (or NF-YC9-∆IDR mutants) on Trp-Leu- medium.tif]

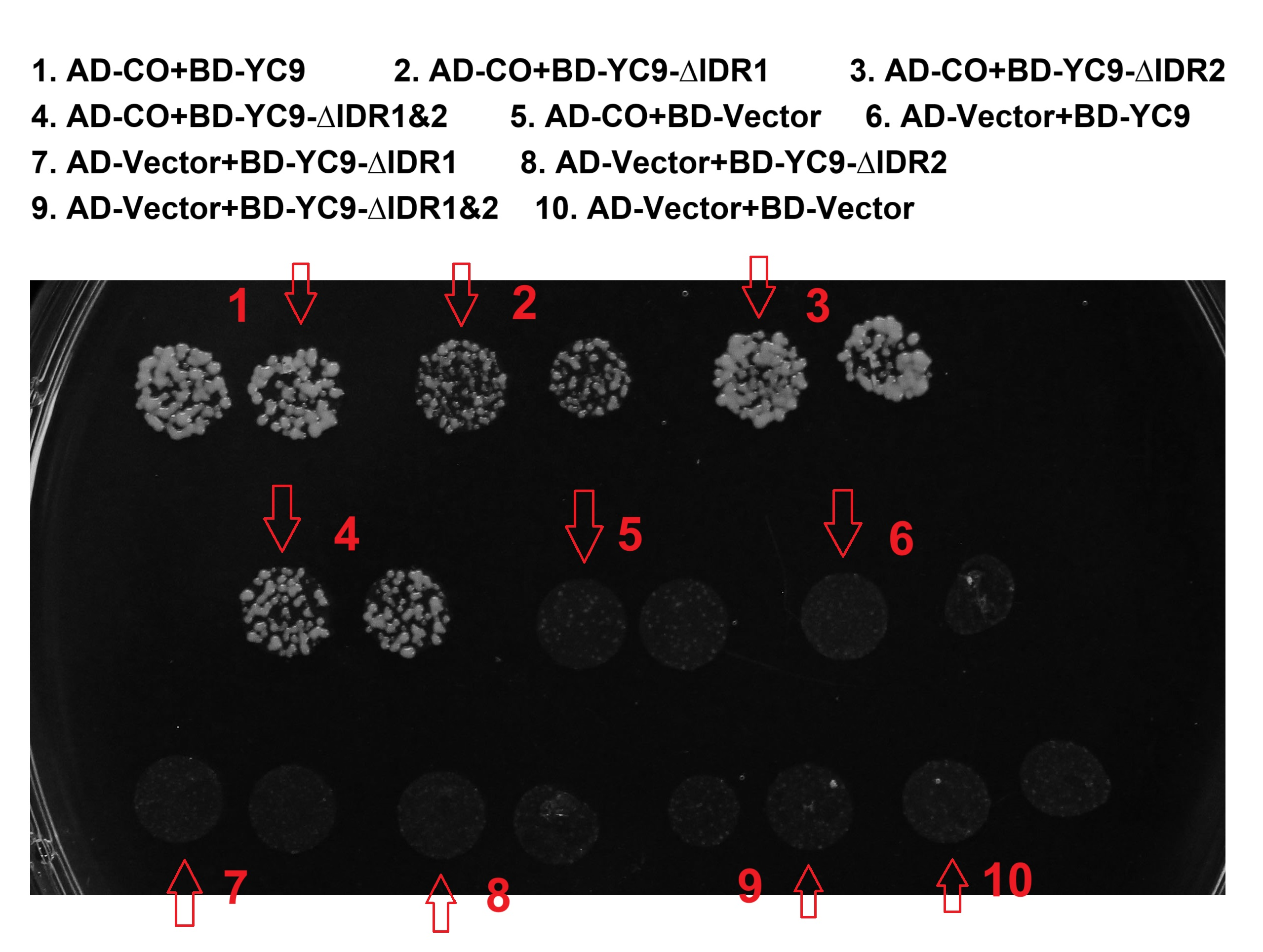

Supplement: Supplementary file 7 — EV Figures Source Data [file 44318_2024_293_MOESM7_ESM.zip › SD Figure EV4/EV4 B/Yeast two-hybrid assays of CO and NF-YC9 (or NF-YC9-∆IDR mutants) on Trp-Leu-His- medium.tif]

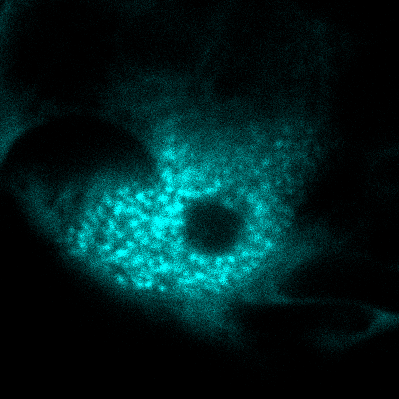

Supplement: Supplementary file 7 — EV Figures Source Data [file 44318_2024_293_MOESM7_ESM.zip › SD Figure EV4/EV4 C/Co-expression of GFP-CO+YC9-∆IDR1&2-mCherry+YB2-BFP in BFP channel.tif]

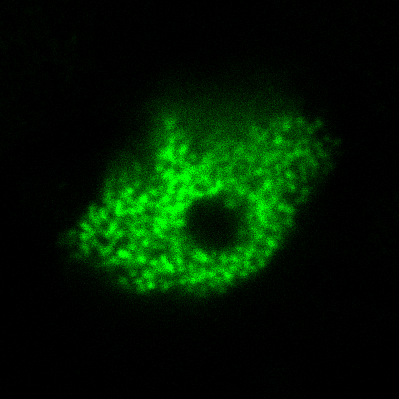

Supplement: Supplementary file 7 — EV Figures Source Data [file 44318_2024_293_MOESM7_ESM.zip › SD Figure EV4/EV4 C/Co-expression of GFP-CO+YC9-∆IDR1&2-mCherry+YB2-BFP in GFP channel.tif]

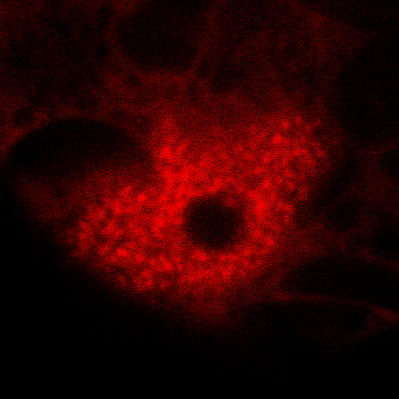

Supplement: Supplementary file 7 — EV Figures Source Data [file 44318_2024_293_MOESM7_ESM.zip › SD Figure EV4/EV4 C/Co-expression of GFP-CO+YC9-∆IDR1&2-mCherry+YB2-BFP in mCherry channel.tif]

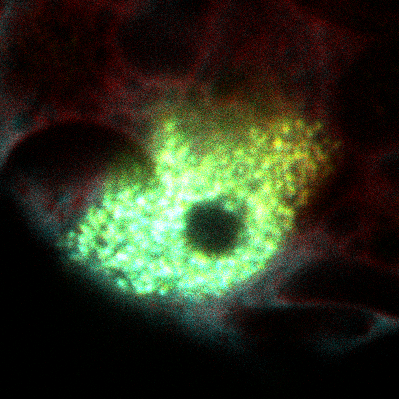

Supplement: Supplementary file 7 — EV Figures Source Data [file 44318_2024_293_MOESM7_ESM.zip › SD Figure EV4/EV4 C/Co-expression of GFP-CO+YC9-∆IDR1&2-mCherry+YB2-BFP in Merge.tif]

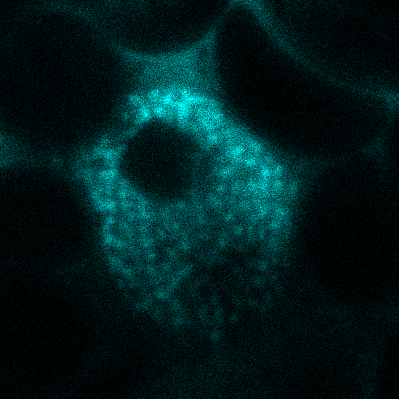

Supplement: Supplementary file 7 — EV Figures Source Data [file 44318_2024_293_MOESM7_ESM.zip › SD Figure EV4/EV4 C/Co-expression of GFP-CO+YC9-∆IDR1-mCherry+YB2-BFP in BFP channel.tif]

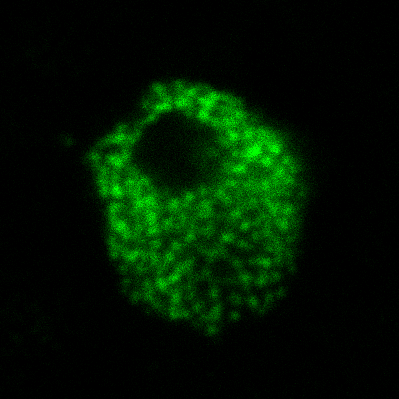

Supplement: Supplementary file 7 — EV Figures Source Data [file 44318_2024_293_MOESM7_ESM.zip › SD Figure EV4/EV4 C/Co-expression of GFP-CO+YC9-∆IDR1-mCherry+YB2-BFP in GFP channel.tif]

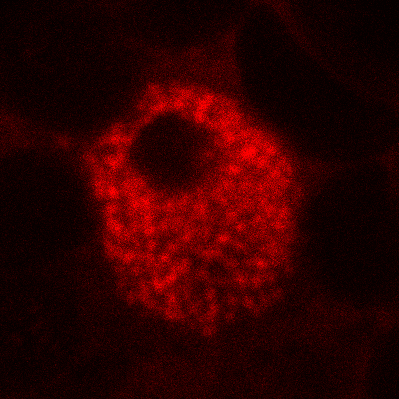

Supplement: Supplementary file 7 — EV Figures Source Data [file 44318_2024_293_MOESM7_ESM.zip › SD Figure EV4/EV4 C/Co-expression of GFP-CO+YC9-∆IDR1-mCherry+YB2-BFP in mCherry channel.tif]

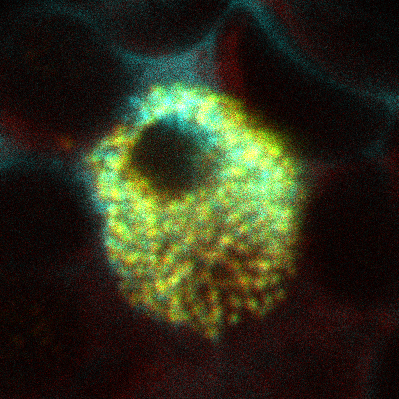

Supplement: Supplementary file 7 — EV Figures Source Data [file 44318_2024_293_MOESM7_ESM.zip › SD Figure EV4/EV4 C/Co-expression of GFP-CO+YC9-∆IDR1-mCherry+YB2-BFP in Merge.tif]

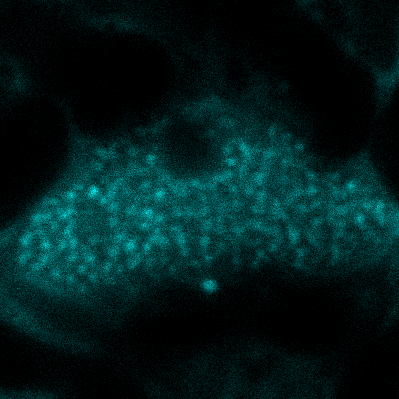

Supplement: Supplementary file 7 — EV Figures Source Data [file 44318_2024_293_MOESM7_ESM.zip › SD Figure EV4/EV4 C/Co-expression of GFP-CO+YC9-∆IDR2-mCherry+YB2-BFP in BFP channel.tif]

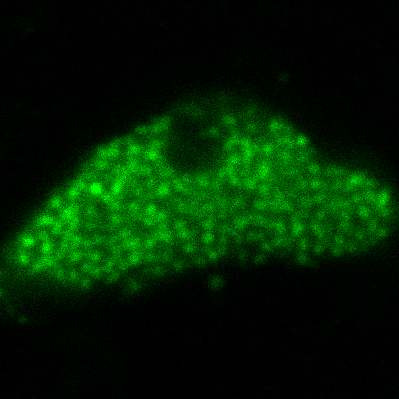

Supplement: Supplementary file 7 — EV Figures Source Data [file 44318_2024_293_MOESM7_ESM.zip › SD Figure EV4/EV4 C/Co-expression of GFP-CO+YC9-∆IDR2-mCherry+YB2-BFP in GFP channel.tif]

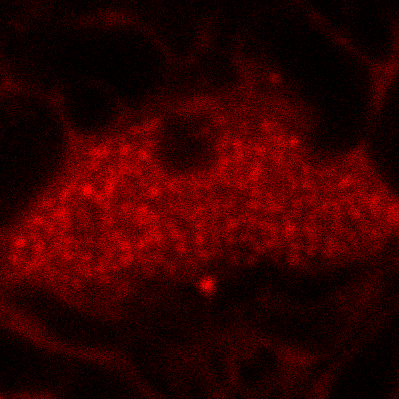

Supplement: Supplementary file 7 — EV Figures Source Data [file 44318_2024_293_MOESM7_ESM.zip › SD Figure EV4/EV4 C/Co-expression of GFP-CO+YC9-∆IDR2-mCherry+YB2-BFP in mCherry channel.tif]

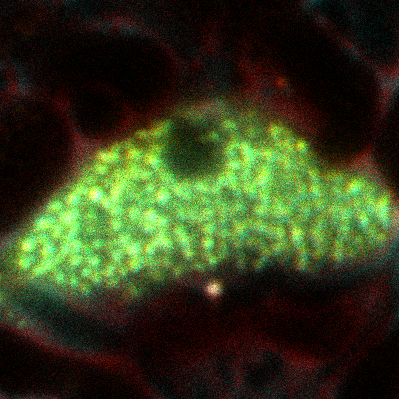

Supplement: Supplementary file 7 — EV Figures Source Data [file 44318_2024_293_MOESM7_ESM.zip › SD Figure EV4/EV4 C/Co-expression of GFP-CO+YC9-∆IDR2-mCherry+YB2-BFP in Merge.tif]

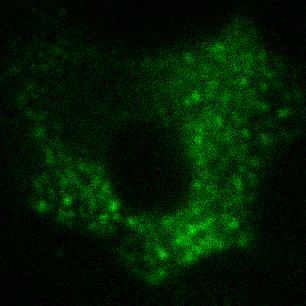

Supplement: Supplementary file 7 — EV Figures Source Data [file 44318_2024_293_MOESM7_ESM.zip › SD Figure EV4/EV4 E/FRAP of GFP-CO spherical condensates (Liquid) in CO+YC9-∆IDR1&2+YB2 co-expression/GFP-CO_FRAP_Postbleach 0s.tif]

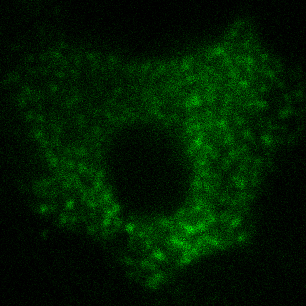

Supplement: Supplementary file 7 — EV Figures Source Data [file 44318_2024_293_MOESM7_ESM.zip › SD Figure EV4/EV4 E/FRAP of GFP-CO spherical condensates (Liquid) in CO+YC9-∆IDR1&2+YB2 co-expression/GFP-CO_FRAP_Postbleach 10s.tif]

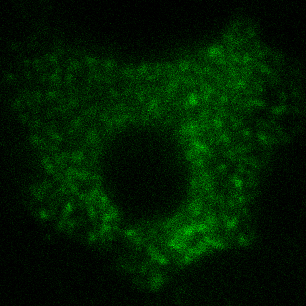

Supplement: Supplementary file 7 — EV Figures Source Data [file 44318_2024_293_MOESM7_ESM.zip › SD Figure EV4/EV4 E/FRAP of GFP-CO spherical condensates (Liquid) in CO+YC9-∆IDR1&2+YB2 co-expression/GFP-CO_FRAP_Postbleach 20s.tif]

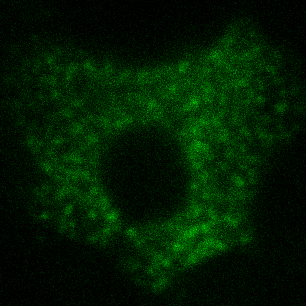

Supplement: Supplementary file 7 — EV Figures Source Data [file 44318_2024_293_MOESM7_ESM.zip › SD Figure EV4/EV4 E/FRAP of GFP-CO spherical condensates (Liquid) in CO+YC9-∆IDR1&2+YB2 co-expression/GFP-CO_FRAP_Postbleach 30s.tif]

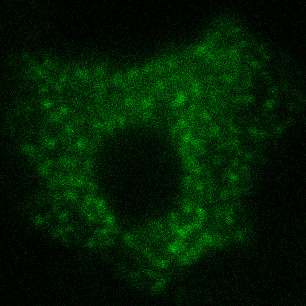

Supplement: Supplementary file 7 — EV Figures Source Data [file 44318_2024_293_MOESM7_ESM.zip › SD Figure EV4/EV4 E/FRAP of GFP-CO spherical condensates (Liquid) in CO+YC9-∆IDR1&2+YB2 co-expression/GFP-CO_FRAP_Postbleach 60s.tif]

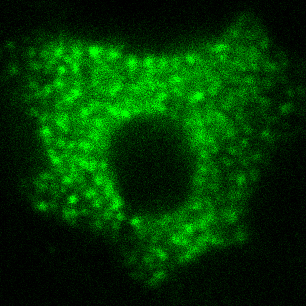

Supplement: Supplementary file 7 — EV Figures Source Data [file 44318_2024_293_MOESM7_ESM.zip › SD Figure EV4/EV4 E/FRAP of GFP-CO spherical condensates (Liquid) in CO+YC9-∆IDR1&2+YB2 co-expression/GFP-CO_FRAP_Prebleach 0s.tif]

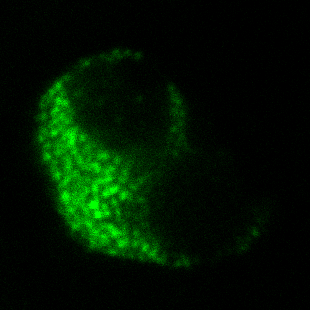

Supplement: Supplementary file 7 — EV Figures Source Data [file 44318_2024_293_MOESM7_ESM.zip › SD Figure EV4/EV4 E/FRAP of GFP-CO spherical condensates (Liquid) in CO+YC9-∆IDR1+YB2 co-expression/GFP-CO_FRAP_Postbleach 0s.tif]

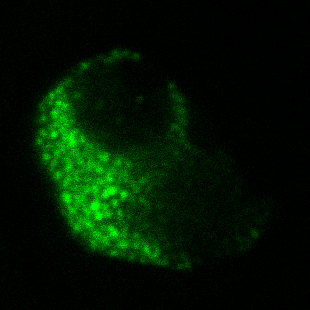

Supplement: Supplementary file 7 — EV Figures Source Data [file 44318_2024_293_MOESM7_ESM.zip › SD Figure EV4/EV4 E/FRAP of GFP-CO spherical condensates (Liquid) in CO+YC9-∆IDR1+YB2 co-expression/GFP-CO_FRAP_Postbleach 10s.tif]

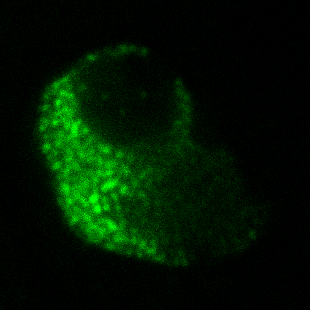

Supplement: Supplementary file 7 — EV Figures Source Data [file 44318_2024_293_MOESM7_ESM.zip › SD Figure EV4/EV4 E/FRAP of GFP-CO spherical condensates (Liquid) in CO+YC9-∆IDR1+YB2 co-expression/GFP-CO_FRAP_Postbleach 20s.tif]

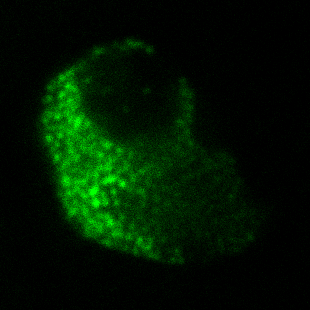

Supplement: Supplementary file 7 — EV Figures Source Data [file 44318_2024_293_MOESM7_ESM.zip › SD Figure EV4/EV4 E/FRAP of GFP-CO spherical condensates (Liquid) in CO+YC9-∆IDR1+YB2 co-expression/GFP-CO_FRAP_Postbleach 30s.tif]

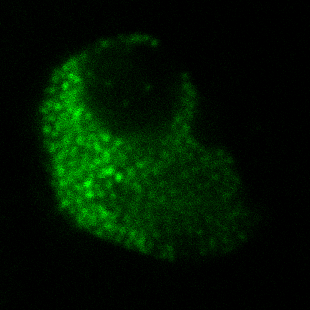

Supplement: Supplementary file 7 — EV Figures Source Data [file 44318_2024_293_MOESM7_ESM.zip › SD Figure EV4/EV4 E/FRAP of GFP-CO spherical condensates (Liquid) in CO+YC9-∆IDR1+YB2 co-expression/GFP-CO_FRAP_Postbleach 60s.tif]

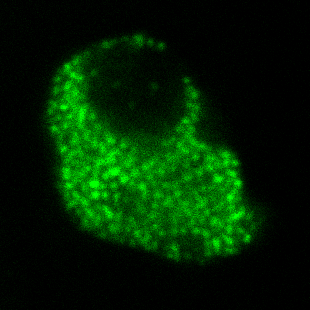

Supplement: Supplementary file 7 — EV Figures Source Data [file 44318_2024_293_MOESM7_ESM.zip › SD Figure EV4/EV4 E/FRAP of GFP-CO spherical condensates (Liquid) in CO+YC9-∆IDR1+YB2 co-expression/GFP-CO_FRAP_Prebleach 0s.tif]

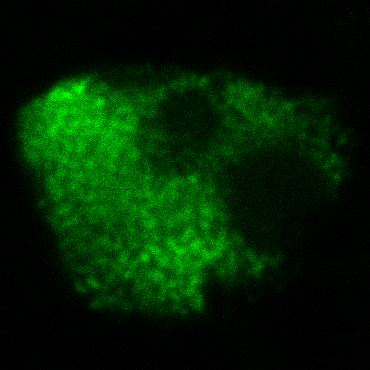

Supplement: Supplementary file 7 — EV Figures Source Data [file 44318_2024_293_MOESM7_ESM.zip › SD Figure EV4/EV4 E/FRAP of GFP-CO spherical condensates (Liquid) in CO+YC9-∆IDR2+YB2 co-expression/GFP-CO_FRAP_Postbleach 0s.tif]

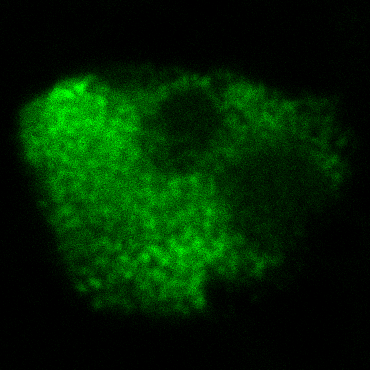

Supplement: Supplementary file 7 — EV Figures Source Data [file 44318_2024_293_MOESM7_ESM.zip › SD Figure EV4/EV4 E/FRAP of GFP-CO spherical condensates (Liquid) in CO+YC9-∆IDR2+YB2 co-expression/GFP-CO_FRAP_Postbleach 10s.tif]

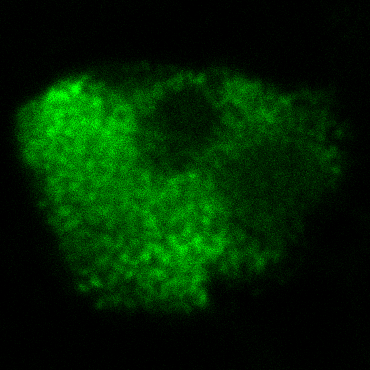

Supplement: Supplementary file 7 — EV Figures Source Data [file 44318_2024_293_MOESM7_ESM.zip › SD Figure EV4/EV4 E/FRAP of GFP-CO spherical condensates (Liquid) in CO+YC9-∆IDR2+YB2 co-expression/GFP-CO_FRAP_Postbleach 20s.tif]

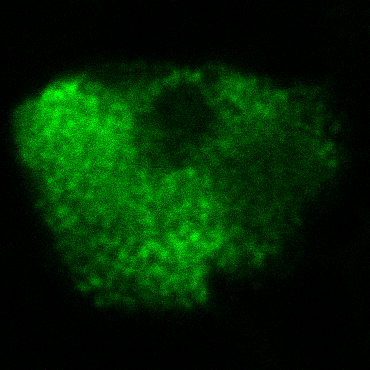

Supplement: Supplementary file 7 — EV Figures Source Data [file 44318_2024_293_MOESM7_ESM.zip › SD Figure EV4/EV4 E/FRAP of GFP-CO spherical condensates (Liquid) in CO+YC9-∆IDR2+YB2 co-expression/GFP-CO_FRAP_Postbleach 30s.tif]

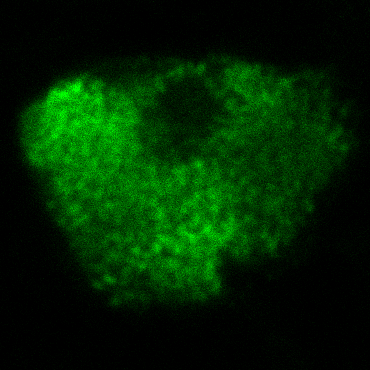

Supplement: Supplementary file 7 — EV Figures Source Data [file 44318_2024_293_MOESM7_ESM.zip › SD Figure EV4/EV4 E/FRAP of GFP-CO spherical condensates (Liquid) in CO+YC9-∆IDR2+YB2 co-expression/GFP-CO_FRAP_Postbleach 60s.tif]

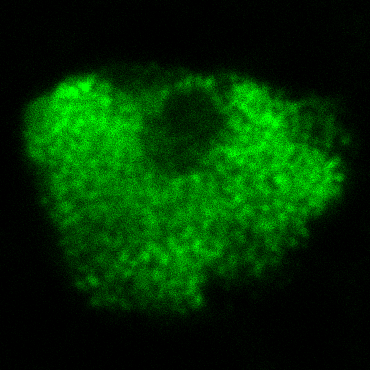

Supplement: Supplementary file 7 — EV Figures Source Data [file 44318_2024_293_MOESM7_ESM.zip › SD Figure EV4/EV4 E/FRAP of GFP-CO spherical condensates (Liquid) in CO+YC9-∆IDR2+YB2 co-expression/GFP-CO_FRAP_Prebleach 0s.tif]

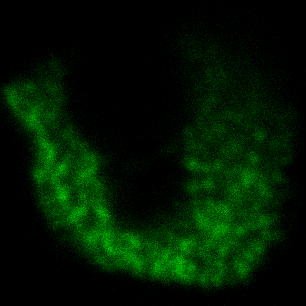

Supplement: Supplementary file 7 — EV Figures Source Data [file 44318_2024_293_MOESM7_ESM.zip › SD Figure EV4/EV4 E/FRAP of GFP-CO spherical condensates (Slow-diffusive) in CO+YC9-∆IDR1&2+YB2 co-expression/GFP-CO_FRAP_Postbleach 0s.tif]

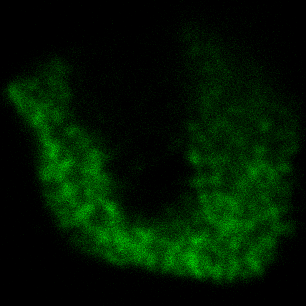

Supplement: Supplementary file 7 — EV Figures Source Data [file 44318_2024_293_MOESM7_ESM.zip › SD Figure EV4/EV4 E/FRAP of GFP-CO spherical condensates (Slow-diffusive) in CO+YC9-∆IDR1&2+YB2 co-expression/GFP-CO_FRAP_Postbleach 10s.tif]

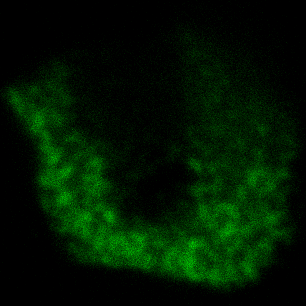

Supplement: Supplementary file 7 — EV Figures Source Data [file 44318_2024_293_MOESM7_ESM.zip › SD Figure EV4/EV4 E/FRAP of GFP-CO spherical condensates (Slow-diffusive) in CO+YC9-∆IDR1&2+YB2 co-expression/GFP-CO_FRAP_Postbleach 20s.tif]

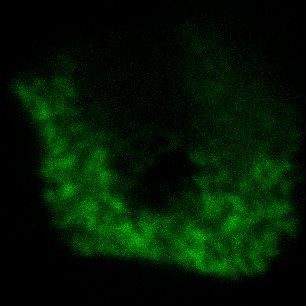

Supplement: Supplementary file 7 — EV Figures Source Data [file 44318_2024_293_MOESM7_ESM.zip › SD Figure EV4/EV4 E/FRAP of GFP-CO spherical condensates (Slow-diffusive) in CO+YC9-∆IDR1&2+YB2 co-expression/GFP-CO_FRAP_Postbleach 30s.tif]

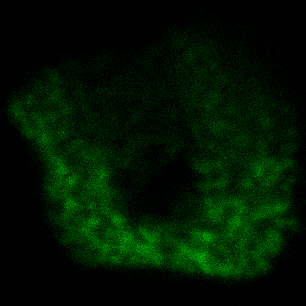

Supplement: Supplementary file 7 — EV Figures Source Data [file 44318_2024_293_MOESM7_ESM.zip › SD Figure EV4/EV4 E/FRAP of GFP-CO spherical condensates (Slow-diffusive) in CO+YC9-∆IDR1&2+YB2 co-expression/GFP-CO_FRAP_Postbleach 60s.tif]

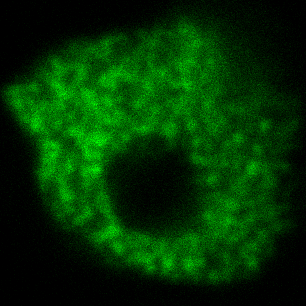

Supplement: Supplementary file 7 — EV Figures Source Data [file 44318_2024_293_MOESM7_ESM.zip › SD Figure EV4/EV4 E/FRAP of GFP-CO spherical condensates (Slow-diffusive) in CO+YC9-∆IDR1&2+YB2 co-expression/GFP-CO_FRAP_Prebleach 0s.tif]

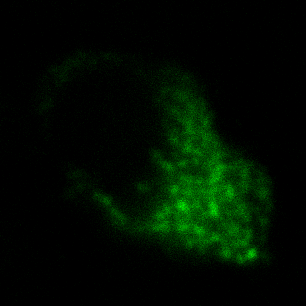

Supplement: Supplementary file 7 — EV Figures Source Data [file 44318_2024_293_MOESM7_ESM.zip › SD Figure EV4/EV4 E/FRAP of GFP-CO spherical condensates (Slow-diffusive) in CO+YC9-∆IDR1+YB2 co-expression/GFP-CO_FRAP_Postbleach 0s.tif]

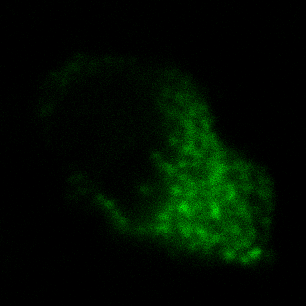

Supplement: Supplementary file 7 — EV Figures Source Data [file 44318_2024_293_MOESM7_ESM.zip › SD Figure EV4/EV4 E/FRAP of GFP-CO spherical condensates (Slow-diffusive) in CO+YC9-∆IDR1+YB2 co-expression/GFP-CO_FRAP_Postbleach 10s.tif]

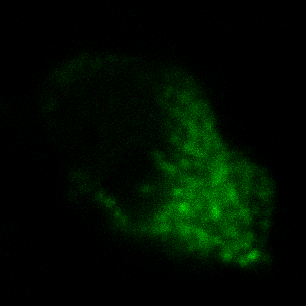

Supplement: Supplementary file 7 — EV Figures Source Data [file 44318_2024_293_MOESM7_ESM.zip › SD Figure EV4/EV4 E/FRAP of GFP-CO spherical condensates (Slow-diffusive) in CO+YC9-∆IDR1+YB2 co-expression/GFP-CO_FRAP_Postbleach 20s.tif]

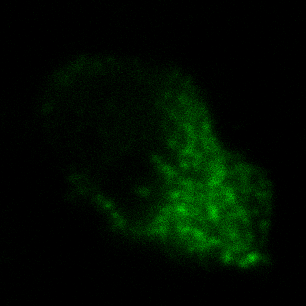

Supplement: Supplementary file 7 — EV Figures Source Data [file 44318_2024_293_MOESM7_ESM.zip › SD Figure EV4/EV4 E/FRAP of GFP-CO spherical condensates (Slow-diffusive) in CO+YC9-∆IDR1+YB2 co-expression/GFP-CO_FRAP_Postbleach 30s.tif]

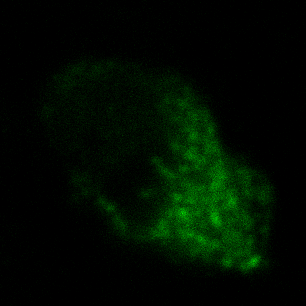

Supplement: Supplementary file 7 — EV Figures Source Data [file 44318_2024_293_MOESM7_ESM.zip › SD Figure EV4/EV4 E/FRAP of GFP-CO spherical condensates (Slow-diffusive) in CO+YC9-∆IDR1+YB2 co-expression/GFP-CO_FRAP_Postbleach 60s.tif]

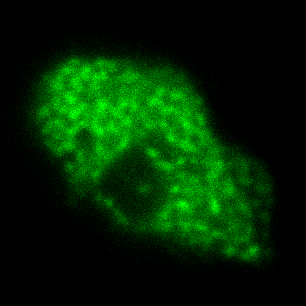

Supplement: Supplementary file 7 — EV Figures Source Data [file 44318_2024_293_MOESM7_ESM.zip › SD Figure EV4/EV4 E/FRAP of GFP-CO spherical condensates (Slow-diffusive) in CO+YC9-∆IDR1+YB2 co-expression/GFP-CO_FRAP_Prebleach 0s.tif]

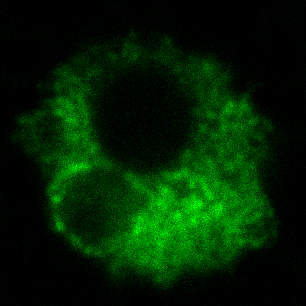

Supplement: Supplementary file 7 — EV Figures Source Data [file 44318_2024_293_MOESM7_ESM.zip › SD Figure EV4/EV4 E/FRAP of GFP-CO spherical condensates (Slow-diffusive) in CO+YC9-∆IDR2+YB2 co-expression/GFP-CO_FRAP_Postbleach 0s.tif]

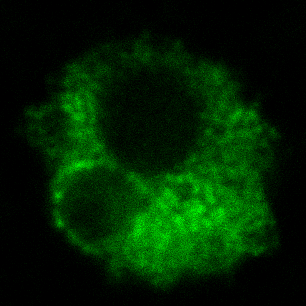

Supplement: Supplementary file 7 — EV Figures Source Data [file 44318_2024_293_MOESM7_ESM.zip › SD Figure EV4/EV4 E/FRAP of GFP-CO spherical condensates (Slow-diffusive) in CO+YC9-∆IDR2+YB2 co-expression/GFP-CO_FRAP_Postbleach 10s.tif]

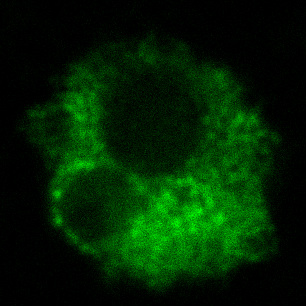

Supplement: Supplementary file 7 — EV Figures Source Data [file 44318_2024_293_MOESM7_ESM.zip › SD Figure EV4/EV4 E/FRAP of GFP-CO spherical condensates (Slow-diffusive) in CO+YC9-∆IDR2+YB2 co-expression/GFP-CO_FRAP_Postbleach 20s.tif]

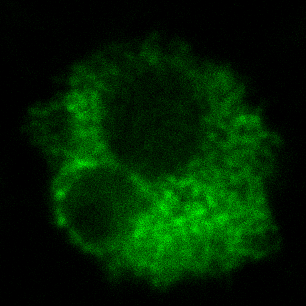

Supplement: Supplementary file 7 — EV Figures Source Data [file 44318_2024_293_MOESM7_ESM.zip › SD Figure EV4/EV4 E/FRAP of GFP-CO spherical condensates (Slow-diffusive) in CO+YC9-∆IDR2+YB2 co-expression/GFP-CO_FRAP_Postbleach 30s.tif]

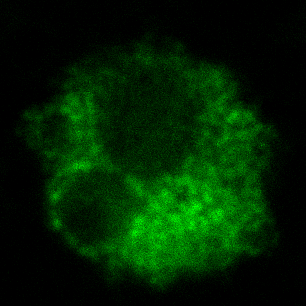

Supplement: Supplementary file 7 — EV Figures Source Data [file 44318_2024_293_MOESM7_ESM.zip › SD Figure EV4/EV4 E/FRAP of GFP-CO spherical condensates (Slow-diffusive) in CO+YC9-∆IDR2+YB2 co-expression/GFP-CO_FRAP_Postbleach 60s.tif]

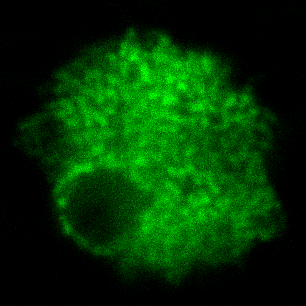

Supplement: Supplementary file 7 — EV Figures Source Data [file 44318_2024_293_MOESM7_ESM.zip › SD Figure EV4/EV4 E/FRAP of GFP-CO spherical condensates (Slow-diffusive) in CO+YC9-∆IDR2+YB2 co-expression/GFP-CO_FRAP_Prebleach 0s.tif]

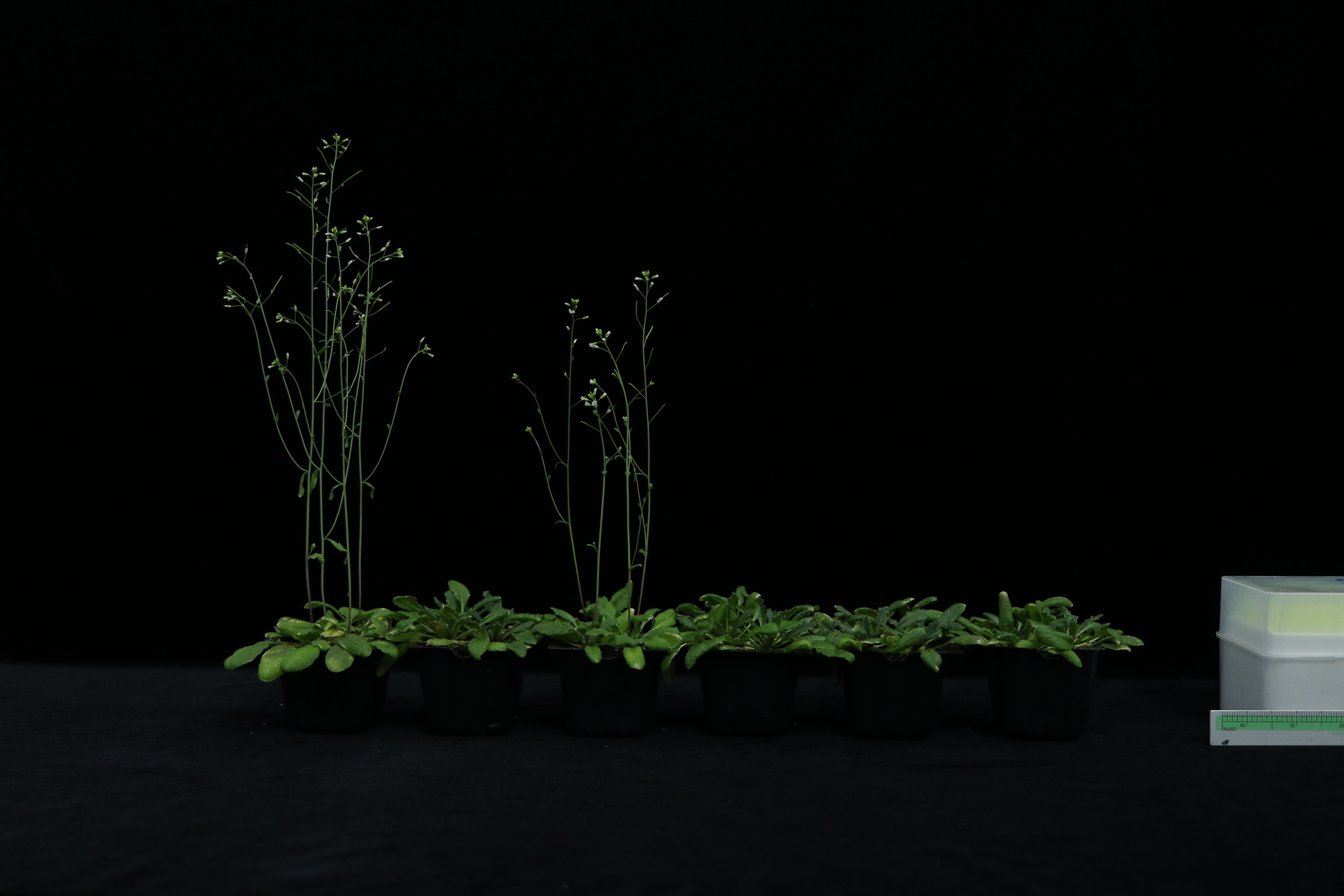

Supplement: Supplementary file 7 — EV Figures Source Data [file 44318_2024_293_MOESM7_ESM.zip › SD Figure EV4/EV4 I/Flowering phenotype of representative plant lines.tif]
