# Supplementary figures and images for: Molecular condensation of the CO/NF-YB/NF-YC/FT complex gates floral transition in Arabidopsis (part 2 of 3)
Source: EMBO J. 2024 Nov 20;44(1):225–50. doi: 10.1038/s44318-024-00293-0 (PMC11696179; doi:10.1038/s44318-024-00293-0)

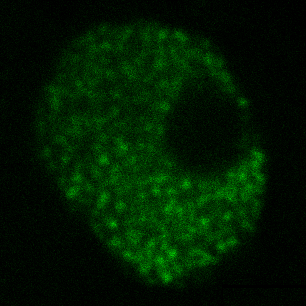

Supplement: Supplementary file 7 — EV Figures Source Data [file 44318_2024_293_MOESM7_ESM.zip › SD Figure EV2/EV2 C/FRAP of GFP-CO spherical condensates (Liquid) in CO+YC9+YB2 co-expression/GFP-CO_FRAP_Postbleach 60s.tif]

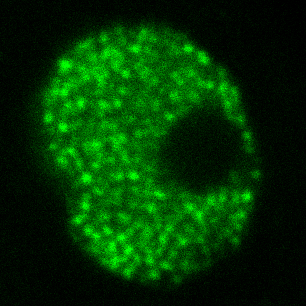

Supplement: Supplementary file 7 — EV Figures Source Data [file 44318_2024_293_MOESM7_ESM.zip › SD Figure EV2/EV2 C/FRAP of GFP-CO spherical condensates (Liquid) in CO+YC9+YB2 co-expression/GFP-CO_FRAP_Prebleach 0s.tif]

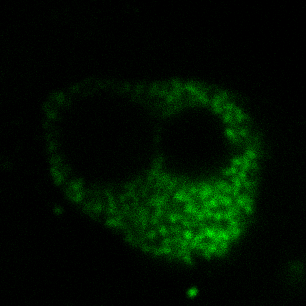

Supplement: Supplementary file 7 — EV Figures Source Data [file 44318_2024_293_MOESM7_ESM.zip › SD Figure EV2/EV2 C/FRAP of GFP-CO spherical condensates (Slow-diffusive) in CO+YC9+YB2 co-expression/GFP-CO_FRAP_Postbleach 0s.tif]

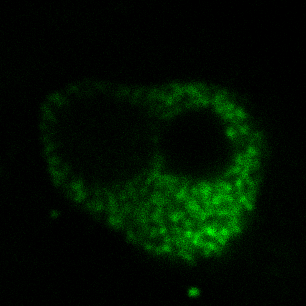

Supplement: Supplementary file 7 — EV Figures Source Data [file 44318_2024_293_MOESM7_ESM.zip › SD Figure EV2/EV2 C/FRAP of GFP-CO spherical condensates (Slow-diffusive) in CO+YC9+YB2 co-expression/GFP-CO_FRAP_Postbleach 10s.tif]

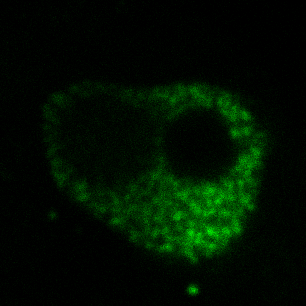

Supplement: Supplementary file 7 — EV Figures Source Data [file 44318_2024_293_MOESM7_ESM.zip › SD Figure EV2/EV2 C/FRAP of GFP-CO spherical condensates (Slow-diffusive) in CO+YC9+YB2 co-expression/GFP-CO_FRAP_Postbleach 20s.tif]

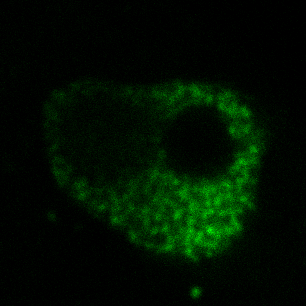

Supplement: Supplementary file 7 — EV Figures Source Data [file 44318_2024_293_MOESM7_ESM.zip › SD Figure EV2/EV2 C/FRAP of GFP-CO spherical condensates (Slow-diffusive) in CO+YC9+YB2 co-expression/GFP-CO_FRAP_Postbleach 30s.tif]

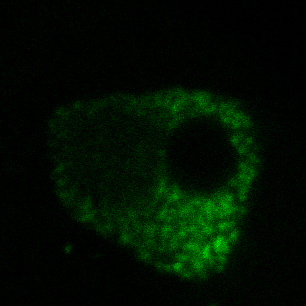

Supplement: Supplementary file 7 — EV Figures Source Data [file 44318_2024_293_MOESM7_ESM.zip › SD Figure EV2/EV2 C/FRAP of GFP-CO spherical condensates (Slow-diffusive) in CO+YC9+YB2 co-expression/GFP-CO_FRAP_Postbleach 60s.tif]

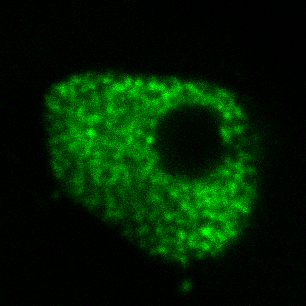

Supplement: Supplementary file 7 — EV Figures Source Data [file 44318_2024_293_MOESM7_ESM.zip › SD Figure EV2/EV2 C/FRAP of GFP-CO spherical condensates (Slow-diffusive) in CO+YC9+YB2 co-expression/GFP-CO_FRAP_Prebleach 0s.tif]

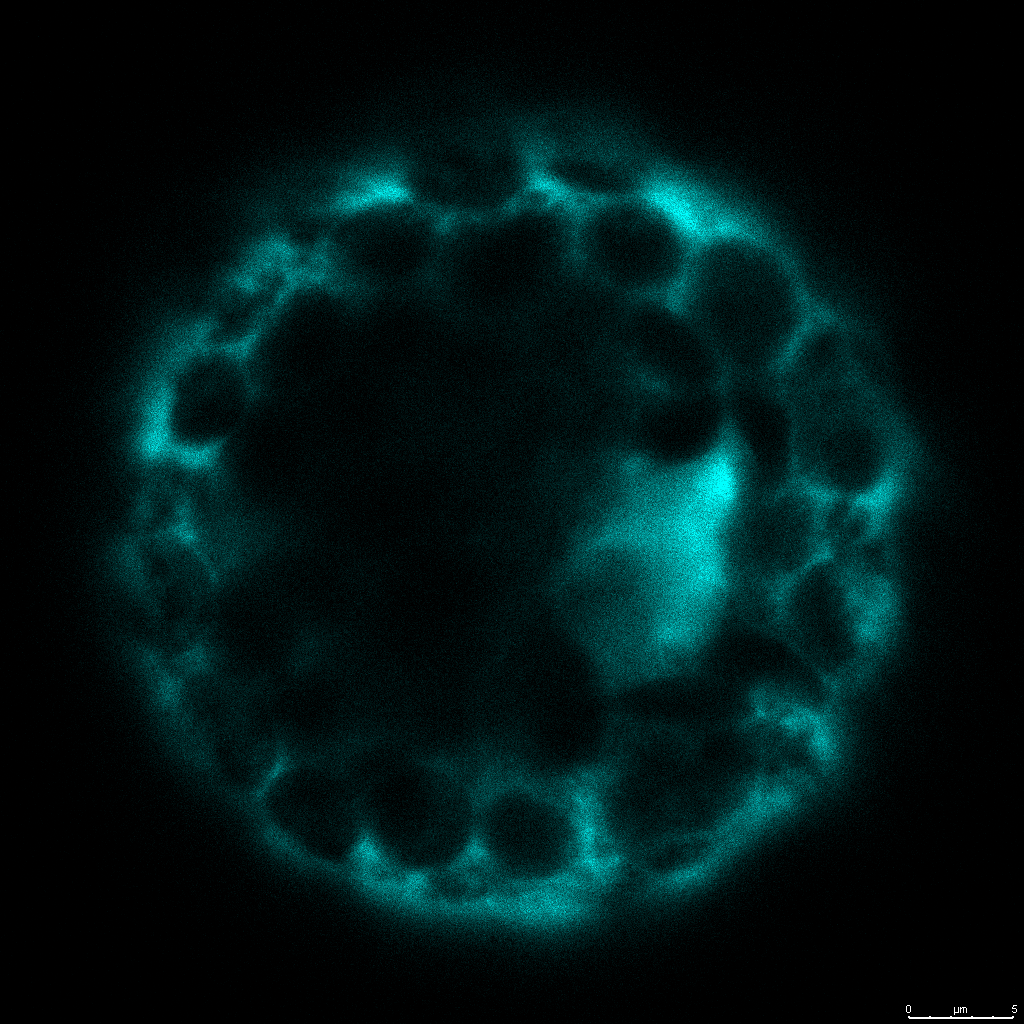

Supplement: Supplementary file 7 — EV Figures Source Data [file 44318_2024_293_MOESM7_ESM.zip › SD Figure EV2/EV2 D/Subcellular localization of YB2-BFP alone.tif]

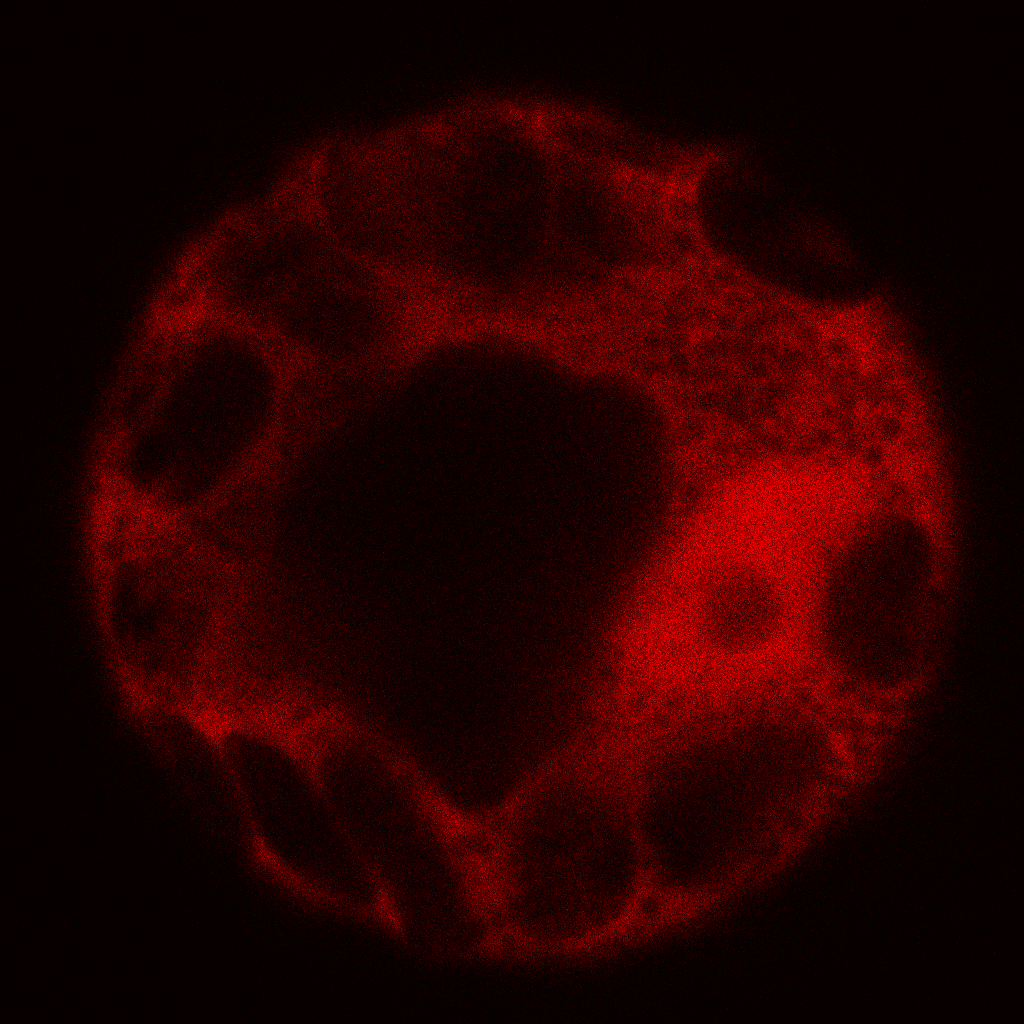

Supplement: Supplementary file 7 — EV Figures Source Data [file 44318_2024_293_MOESM7_ESM.zip › SD Figure EV2/EV2 D/Subcellular localization of YC9-mCherry alone.tif]

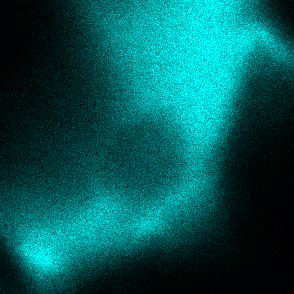

Supplement: Supplementary file 7 — EV Figures Source Data [file 44318_2024_293_MOESM7_ESM.zip › SD Figure EV2/EV2 E/Co-expression of YC9-mCherry and YB2-BFP in BFP channel.tif]

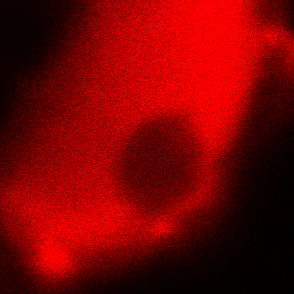

Supplement: Supplementary file 7 — EV Figures Source Data [file 44318_2024_293_MOESM7_ESM.zip › SD Figure EV2/EV2 E/Co-expression of YC9-mCherry and YB2-BFP in mCherry channel.tif]

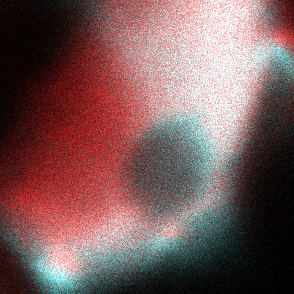

Supplement: Supplementary file 7 — EV Figures Source Data [file 44318_2024_293_MOESM7_ESM.zip › SD Figure EV2/EV2 E/Co-expression of YC9-mCherry and YB2-BFP in Merge.tif]

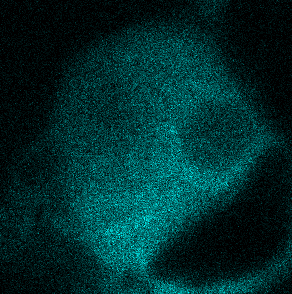

Supplement: Supplementary file 7 — EV Figures Source Data [file 44318_2024_293_MOESM7_ESM.zip › SD Figure EV2/EV2 F/Co-expression of GFP-CO+YB2-BFP_1 in BFP channel.tif]

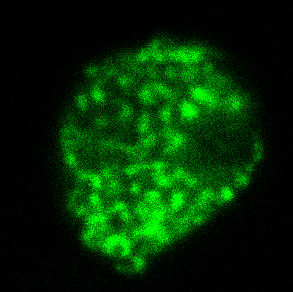

Supplement: Supplementary file 7 — EV Figures Source Data [file 44318_2024_293_MOESM7_ESM.zip › SD Figure EV2/EV2 F/Co-expression of GFP-CO+YB2-BFP_1 in GFP channel.tif]

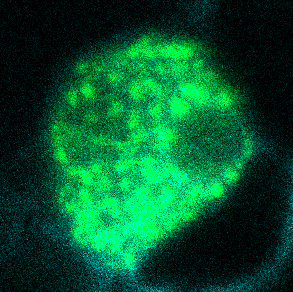

Supplement: Supplementary file 7 — EV Figures Source Data [file 44318_2024_293_MOESM7_ESM.zip › SD Figure EV2/EV2 F/Co-expression of GFP-CO+YB2-BFP_1 in Merge.tif]

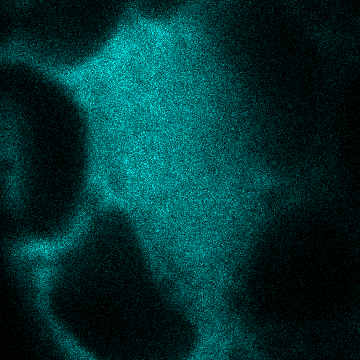

Supplement: Supplementary file 7 — EV Figures Source Data [file 44318_2024_293_MOESM7_ESM.zip › SD Figure EV2/EV2 F/Co-expression of GFP-CO+YB2-BFP_2 in BFP channel.tif]

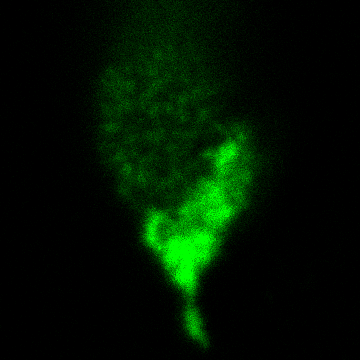

Supplement: Supplementary file 7 — EV Figures Source Data [file 44318_2024_293_MOESM7_ESM.zip › SD Figure EV2/EV2 F/Co-expression of GFP-CO+YB2-BFP_2 in GFP channel.tif]

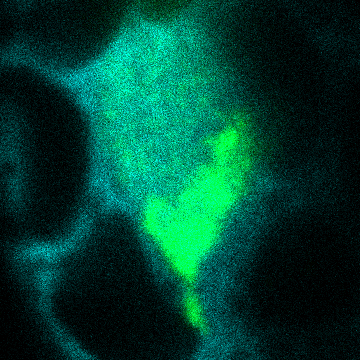

Supplement: Supplementary file 7 — EV Figures Source Data [file 44318_2024_293_MOESM7_ESM.zip › SD Figure EV2/EV2 F/Co-expression of GFP-CO+YB2-BFP_2 in Merge.tif]

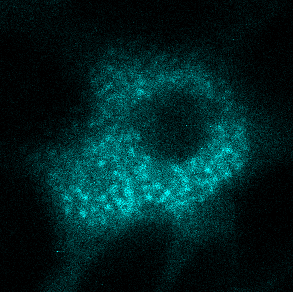

Supplement: Supplementary file 7 — EV Figures Source Data [file 44318_2024_293_MOESM7_ESM.zip › SD Figure EV2/EV2 F/Co-expression of GFP-CO+YC9-mCherry+YB2-BFP in BFP channel.tif]

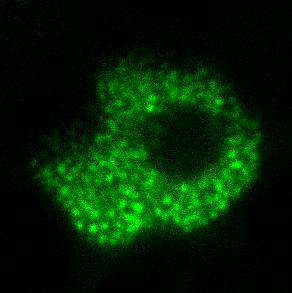

Supplement: Supplementary file 7 — EV Figures Source Data [file 44318_2024_293_MOESM7_ESM.zip › SD Figure EV2/EV2 F/Co-expression of GFP-CO+YC9-mCherry+YB2-BFP in GFP channel.tif]

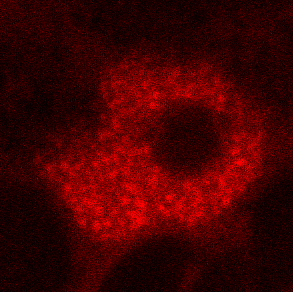

Supplement: Supplementary file 7 — EV Figures Source Data [file 44318_2024_293_MOESM7_ESM.zip › SD Figure EV2/EV2 F/Co-expression of GFP-CO+YC9-mCherry+YB2-BFP in mCherry channel.tif]

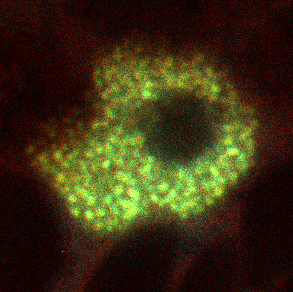

Supplement: Supplementary file 7 — EV Figures Source Data [file 44318_2024_293_MOESM7_ESM.zip › SD Figure EV2/EV2 F/Co-expression of GFP-CO+YC9-mCherry+YB2-BFP in Merge.tif]

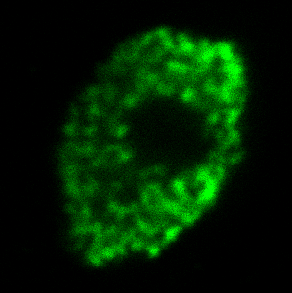

Supplement: Supplementary file 7 — EV Figures Source Data [file 44318_2024_293_MOESM7_ESM.zip › SD Figure EV2/EV2 F/Co-expression of GFP-CO+YC9-mCherry_1 in GFP channel.tif]

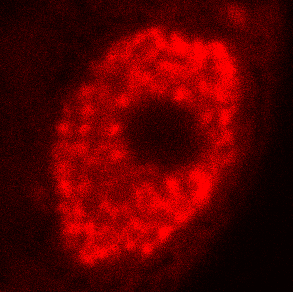

Supplement: Supplementary file 7 — EV Figures Source Data [file 44318_2024_293_MOESM7_ESM.zip › SD Figure EV2/EV2 F/Co-expression of GFP-CO+YC9-mCherry_1 in mCherry channel.tif]

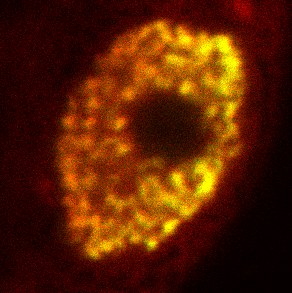

Supplement: Supplementary file 7 — EV Figures Source Data [file 44318_2024_293_MOESM7_ESM.zip › SD Figure EV2/EV2 F/Co-expression of GFP-CO+YC9-mCherry_1 in Merge.tif]

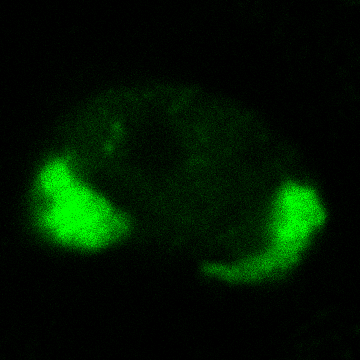

Supplement: Supplementary file 7 — EV Figures Source Data [file 44318_2024_293_MOESM7_ESM.zip › SD Figure EV2/EV2 F/Co-expression of GFP-CO+YC9-mCherry_2 in GFP channel.tif]

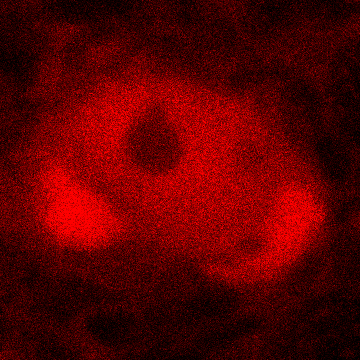

Supplement: Supplementary file 7 — EV Figures Source Data [file 44318_2024_293_MOESM7_ESM.zip › SD Figure EV2/EV2 F/Co-expression of GFP-CO+YC9-mCherry_2 in mCherry channel.tif]

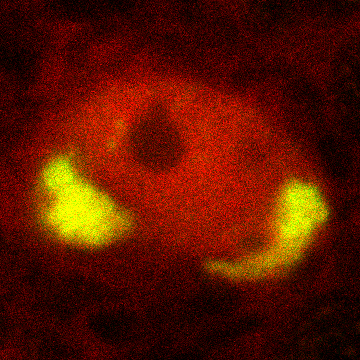

Supplement: Supplementary file 7 — EV Figures Source Data [file 44318_2024_293_MOESM7_ESM.zip › SD Figure EV2/EV2 F/Co-expression of GFP-CO+YC9-mCherry_2 in Merge.tif]

## Slide 1
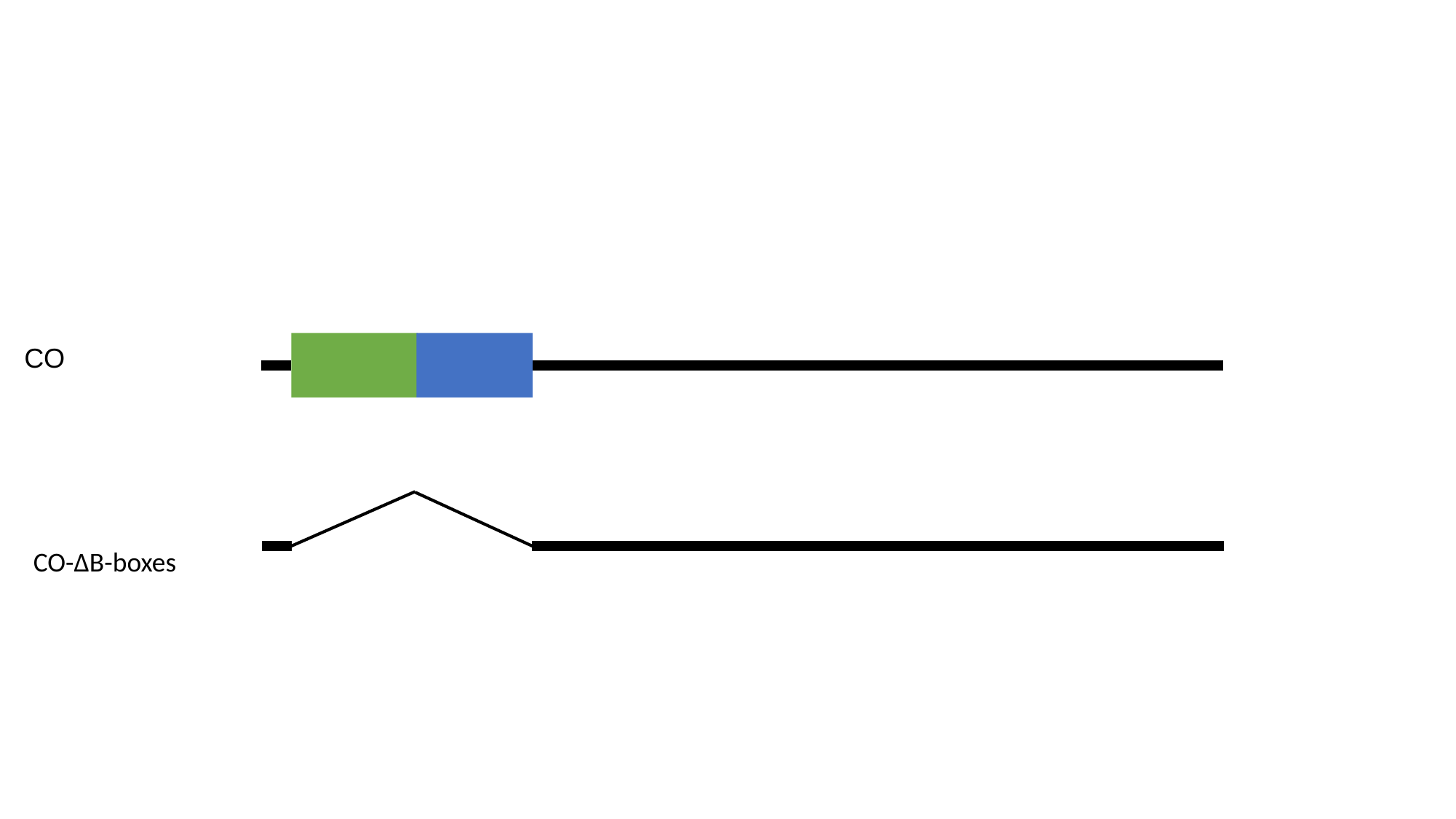

CO
CO-ΔB-boxes

Supplement: Supplementary file 7 — EV Figures Source Data [file 44318_2024_293_MOESM7_ESM.zip › SD Figure EV2/EV2 G/Schematic of CO and CO-∆B-boxes.pptx]

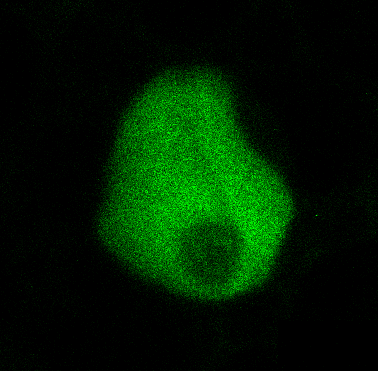

Supplement: Supplementary file 7 — EV Figures Source Data [file 44318_2024_293_MOESM7_ESM.zip › SD Figure EV2/EV2 G/Subcellular localization of GFP-CO-∆B-boxes.tif]

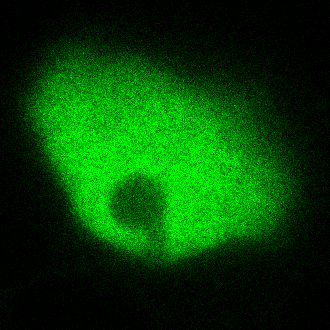

Supplement: Supplementary file 7 — EV Figures Source Data [file 44318_2024_293_MOESM7_ESM.zip › SD Figure EV2/EV2 H/Co-expression of GFP-CO-∆B-box+YC9-mCherry in GFP channel.tif]

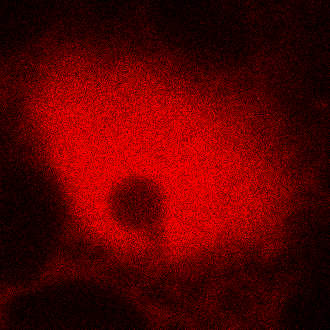

Supplement: Supplementary file 7 — EV Figures Source Data [file 44318_2024_293_MOESM7_ESM.zip › SD Figure EV2/EV2 H/Co-expression of GFP-CO-∆B-box+YC9-mCherry in mCherry channel.tif]

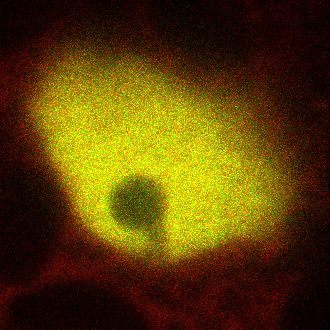

Supplement: Supplementary file 7 — EV Figures Source Data [file 44318_2024_293_MOESM7_ESM.zip › SD Figure EV2/EV2 H/Co-expression of GFP-CO-∆B-box+YC9-mCherry in Merge.tif]

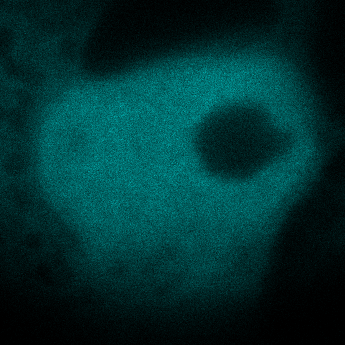

Supplement: Supplementary file 7 — EV Figures Source Data [file 44318_2024_293_MOESM7_ESM.zip › SD Figure EV2/EV2 H/Co-expression of GFP-CO-∆B-box+YC9-mCherry+YB2-BFP in BFP channel.tif]

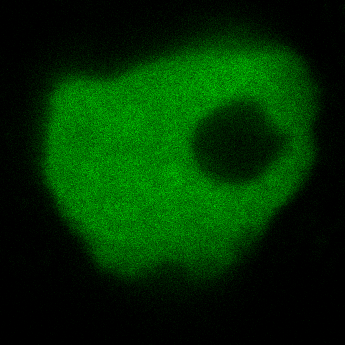

Supplement: Supplementary file 7 — EV Figures Source Data [file 44318_2024_293_MOESM7_ESM.zip › SD Figure EV2/EV2 H/Co-expression of GFP-CO-∆B-box+YC9-mCherry+YB2-BFP in GFP channel.tif]

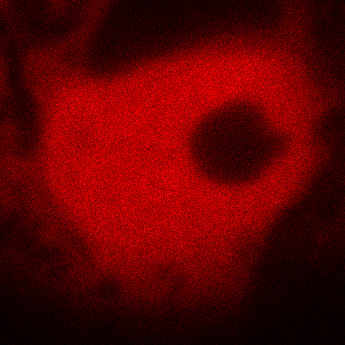

Supplement: Supplementary file 7 — EV Figures Source Data [file 44318_2024_293_MOESM7_ESM.zip › SD Figure EV2/EV2 H/Co-expression of GFP-CO-∆B-box+YC9-mCherry+YB2-BFP in mCherry channel.tif]

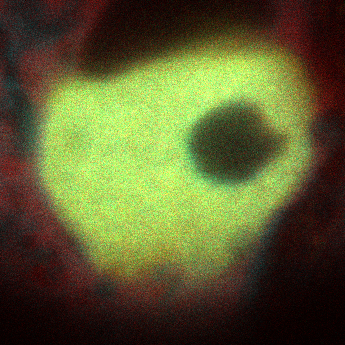

Supplement: Supplementary file 7 — EV Figures Source Data [file 44318_2024_293_MOESM7_ESM.zip › SD Figure EV2/EV2 H/Co-expression of GFP-CO-∆B-box+YC9-mCherry+YB2-BFP in Merge.tif]

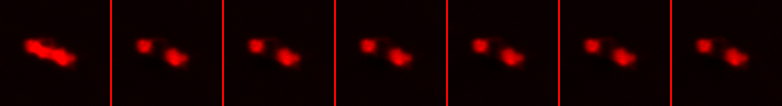

Supplement: Supplementary file 7 — EV Figures Source Data [file 44318_2024_293_MOESM7_ESM.zip › SD Figure EV3/EV3 A/mCherry-CO.tif]

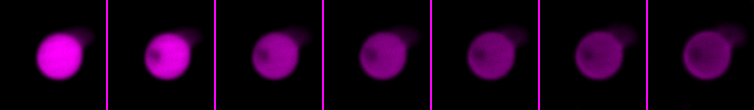

Supplement: Supplementary file 7 — EV Figures Source Data [file 44318_2024_293_MOESM7_ESM.zip › SD Figure EV3/EV3 A/YB2-Alexa 647.jpg]

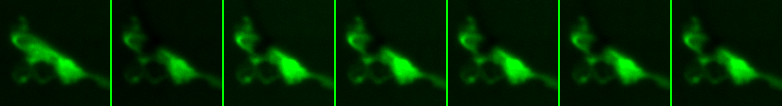

Supplement: Supplementary file 7 — EV Figures Source Data [file 44318_2024_293_MOESM7_ESM.zip › SD Figure EV3/EV3 A/YC9-GFP.tif]

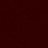

Supplement: Supplementary file 7 — EV Figures Source Data [file 44318_2024_293_MOESM7_ESM.zip › SD Figure EV3/EV3 C&D/0.25uM YB2 in 100mM NaCl-zoom in.tif]

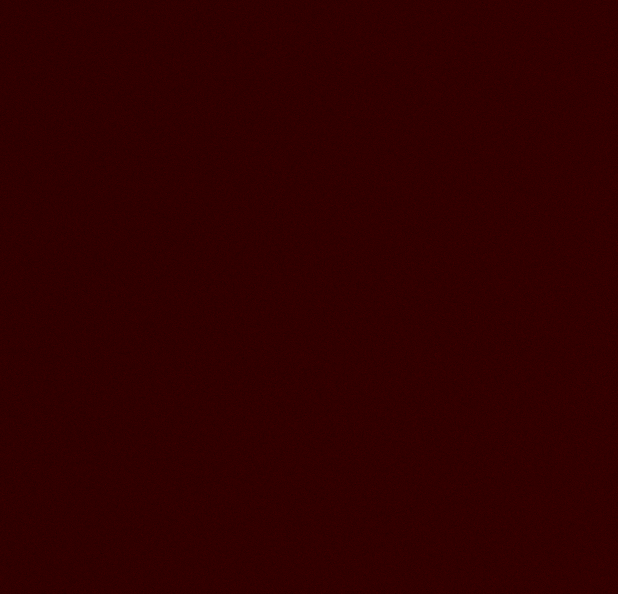

Supplement: Supplementary file 7 — EV Figures Source Data [file 44318_2024_293_MOESM7_ESM.zip › SD Figure EV3/EV3 C&D/0.25uM YB2 in 100mM NaCl.tif]

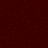

Supplement: Supplementary file 7 — EV Figures Source Data [file 44318_2024_293_MOESM7_ESM.zip › SD Figure EV3/EV3 C&D/0.25uM YB2 in 150mM NaCl-zoom in.tif]

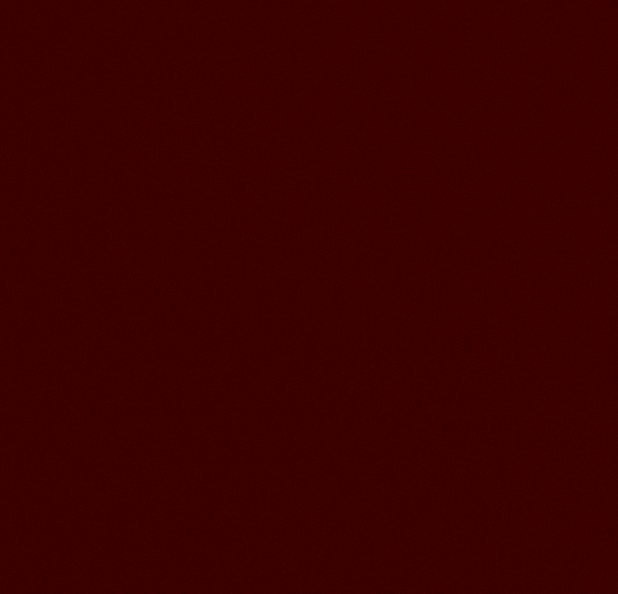

Supplement: Supplementary file 7 — EV Figures Source Data [file 44318_2024_293_MOESM7_ESM.zip › SD Figure EV3/EV3 C&D/0.25uM YB2 in 150mM NaCl.tif]

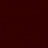

Supplement: Supplementary file 7 — EV Figures Source Data [file 44318_2024_293_MOESM7_ESM.zip › SD Figure EV3/EV3 C&D/0.25uM YB2 in 300mM NaCl-zoom in.tif]

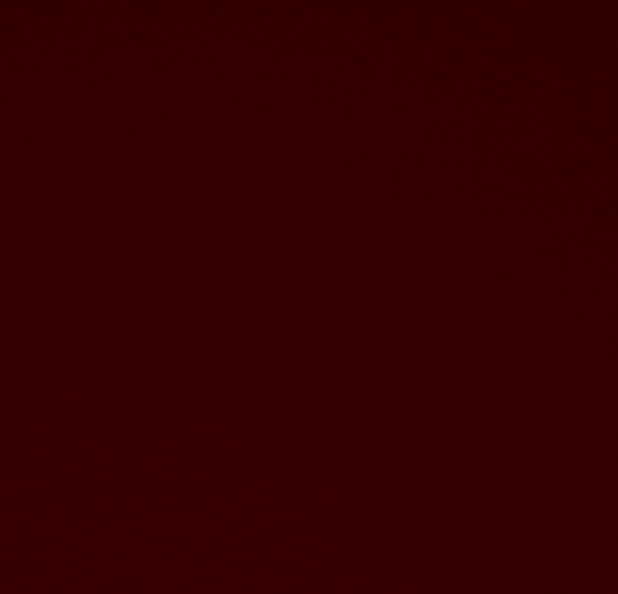

Supplement: Supplementary file 7 — EV Figures Source Data [file 44318_2024_293_MOESM7_ESM.zip › SD Figure EV3/EV3 C&D/0.25uM YB2 in 300mM NaCl.tif]

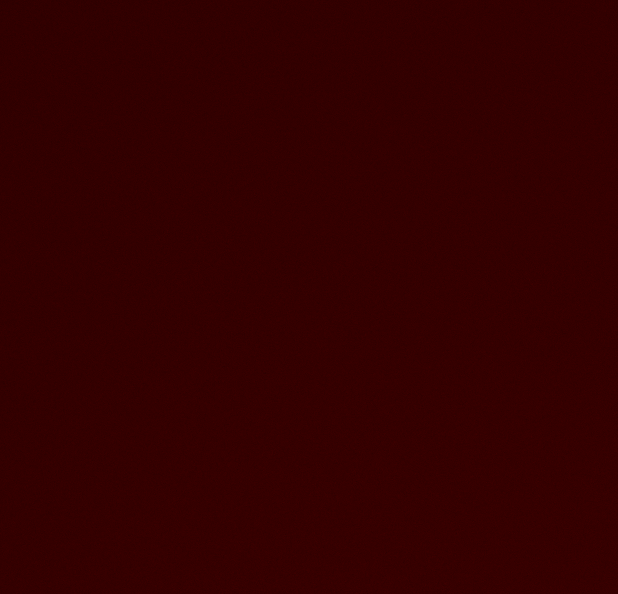

Supplement: Supplementary file 7 — EV Figures Source Data [file 44318_2024_293_MOESM7_ESM.zip › SD Figure EV3/EV3 C&D/0.25uM YB2 in 450mM NaCl.tif]

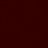

Supplement: Supplementary file 7 — EV Figures Source Data [file 44318_2024_293_MOESM7_ESM.zip › SD Figure EV3/EV3 C&D/0.25uM YB2 in 450mM NaCl_zoom in.tif]

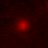

Supplement: Supplementary file 7 — EV Figures Source Data [file 44318_2024_293_MOESM7_ESM.zip › SD Figure EV3/EV3 C&D/0.5uM YB2 in 100mM Nacl-zoom in.tif]

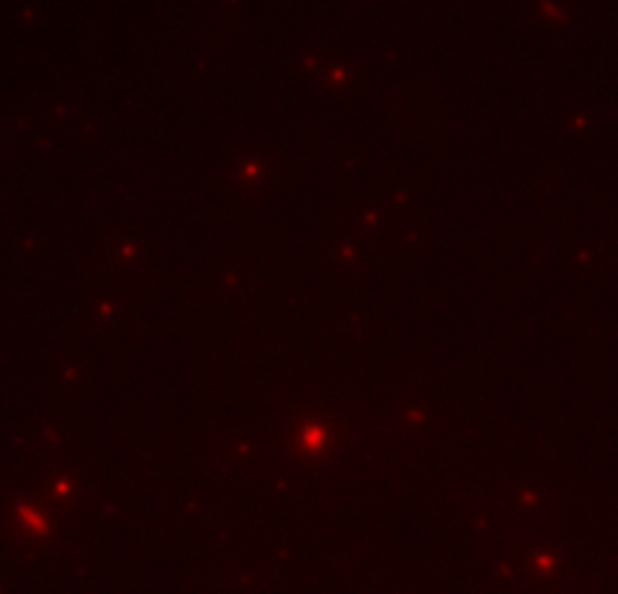

Supplement: Supplementary file 7 — EV Figures Source Data [file 44318_2024_293_MOESM7_ESM.zip › SD Figure EV3/EV3 C&D/0.5uM YB2 in 100mM Nacl.tif]

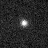

Supplement: Supplementary file 7 — EV Figures Source Data [file 44318_2024_293_MOESM7_ESM.zip › SD Figure EV3/EV3 C&D/0.5uM YB2 in 150mM Nacl-zoom in.tif]

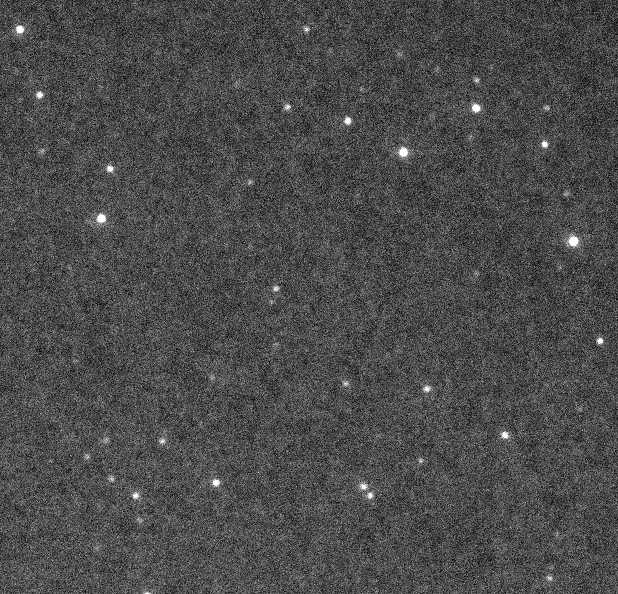

Supplement: Supplementary file 7 — EV Figures Source Data [file 44318_2024_293_MOESM7_ESM.zip › SD Figure EV3/EV3 C&D/0.5uM YB2 in 150mM Nacl.tif]

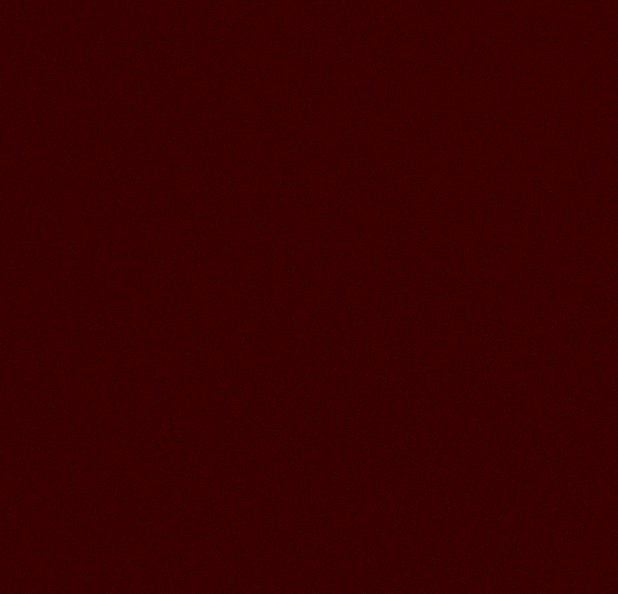

Supplement: Supplementary file 7 — EV Figures Source Data [file 44318_2024_293_MOESM7_ESM.zip › SD Figure EV3/EV3 C&D/0.5uM YB2 in 300mM NaCl.tif]

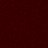

Supplement: Supplementary file 7 — EV Figures Source Data [file 44318_2024_293_MOESM7_ESM.zip › SD Figure EV3/EV3 C&D/0.5uM YB2 in 300mM NaCl_zoom in.tif]

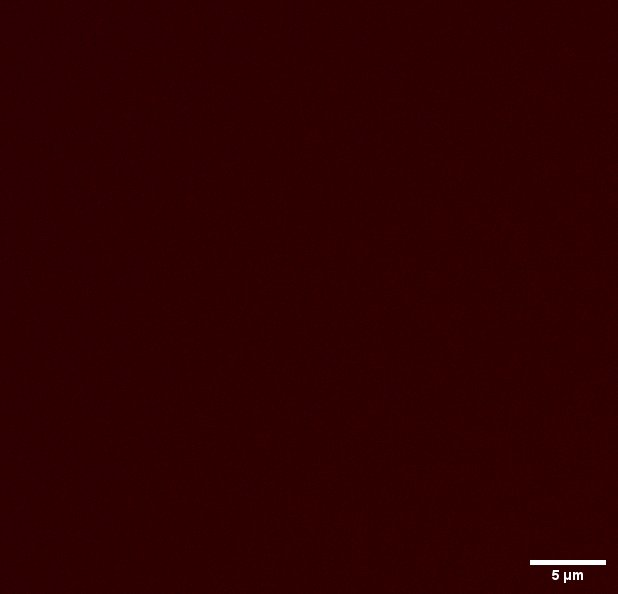

Supplement: Supplementary file 7 — EV Figures Source Data [file 44318_2024_293_MOESM7_ESM.zip › SD Figure EV3/EV3 C&D/0.5uM YB2 in 450mM NaCl.tif]

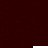

Supplement: Supplementary file 7 — EV Figures Source Data [file 44318_2024_293_MOESM7_ESM.zip › SD Figure EV3/EV3 C&D/0.5uM YB2 in 450mM NaCl_zoom in.tif]

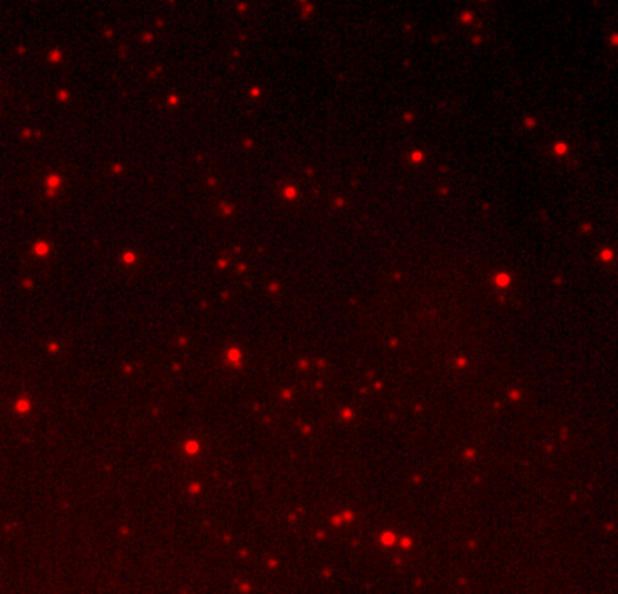

Supplement: Supplementary file 7 — EV Figures Source Data [file 44318_2024_293_MOESM7_ESM.zip › SD Figure EV3/EV3 C&D/1uM YB2 in 100mM NaCl.tif]

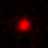

Supplement: Supplementary file 7 — EV Figures Source Data [file 44318_2024_293_MOESM7_ESM.zip › SD Figure EV3/EV3 C&D/1uM YB2 in 100mM NaCl_zoom in.tif]

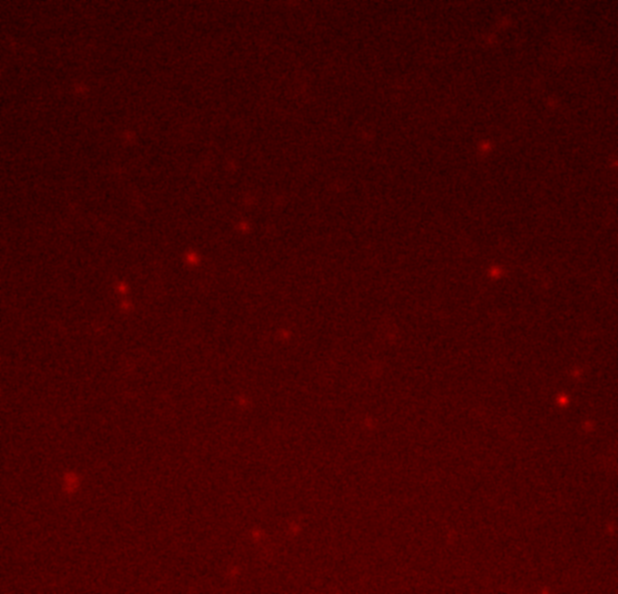

Supplement: Supplementary file 7 — EV Figures Source Data [file 44318_2024_293_MOESM7_ESM.zip › SD Figure EV3/EV3 C&D/1uM YB2 in 150mM NaCl.tif]

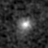

Supplement: Supplementary file 7 — EV Figures Source Data [file 44318_2024_293_MOESM7_ESM.zip › SD Figure EV3/EV3 C&D/1uM YB2 in 150mM NaCl_zoom in.tif]

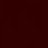

Supplement: Supplementary file 7 — EV Figures Source Data [file 44318_2024_293_MOESM7_ESM.zip › SD Figure EV3/EV3 C&D/1uM YB2 in 300mM NaCl-zoom in.tif]

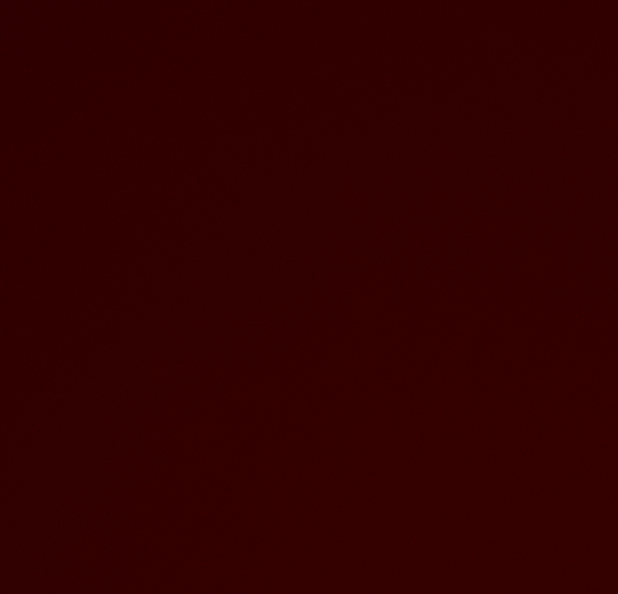

Supplement: Supplementary file 7 — EV Figures Source Data [file 44318_2024_293_MOESM7_ESM.zip › SD Figure EV3/EV3 C&D/1uM YB2 in 300mM NaCl.tif]

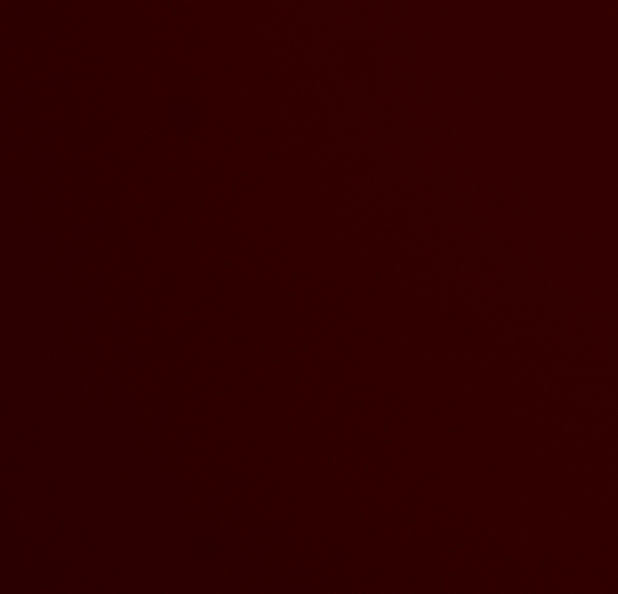

Supplement: Supplementary file 7 — EV Figures Source Data [file 44318_2024_293_MOESM7_ESM.zip › SD Figure EV3/EV3 C&D/1uM YB2 in 450mM NaCl.tif]

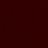

Supplement: Supplementary file 7 — EV Figures Source Data [file 44318_2024_293_MOESM7_ESM.zip › SD Figure EV3/EV3 C&D/1uM YB2 in 450mM NaCl_zoom in.tif]

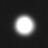

Supplement: Supplementary file 7 — EV Figures Source Data [file 44318_2024_293_MOESM7_ESM.zip › SD Figure EV3/EV3 C&D/20uM YB2 in 100mM NaCl-zoom in.tif]

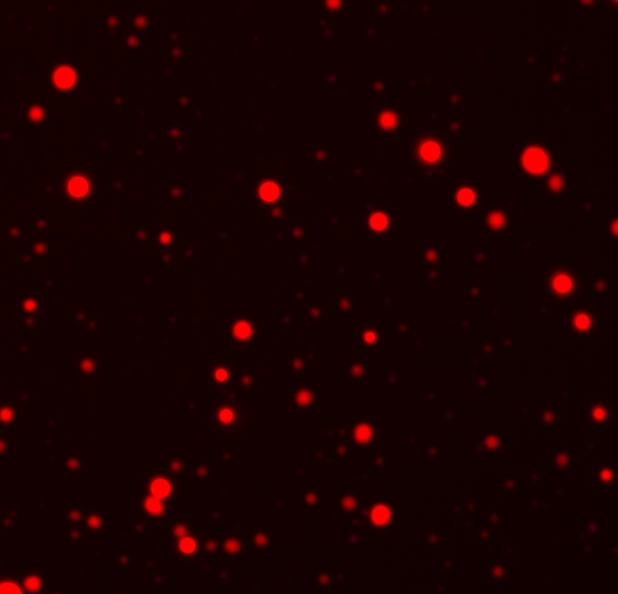

Supplement: Supplementary file 7 — EV Figures Source Data [file 44318_2024_293_MOESM7_ESM.zip › SD Figure EV3/EV3 C&D/20uM YB2 in 100mM NaCl.tif]

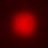

Supplement: Supplementary file 7 — EV Figures Source Data [file 44318_2024_293_MOESM7_ESM.zip › SD Figure EV3/EV3 C&D/20uM YB2 in 150mM NaCl-zoom in.tif]

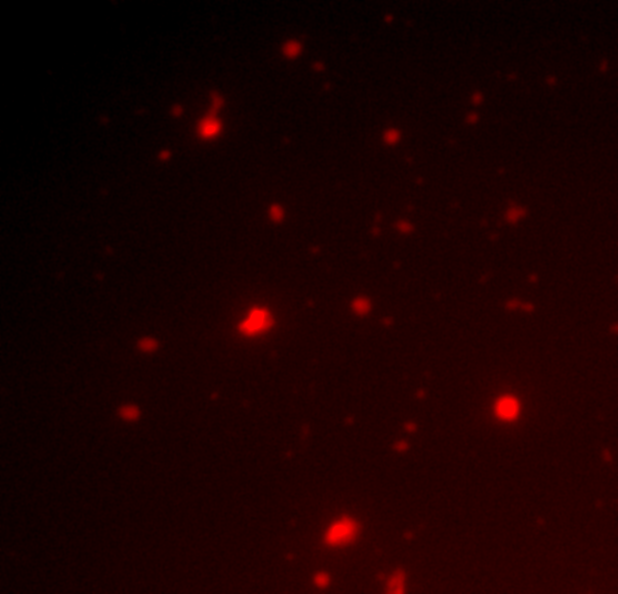

Supplement: Supplementary file 7 — EV Figures Source Data [file 44318_2024_293_MOESM7_ESM.zip › SD Figure EV3/EV3 C&D/20uM YB2 in 150mM NaCl.tif]

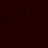

Supplement: Supplementary file 7 — EV Figures Source Data [file 44318_2024_293_MOESM7_ESM.zip › SD Figure EV3/EV3 C&D/20uM YB2 in 300mM NaCl-zoom in.tif]

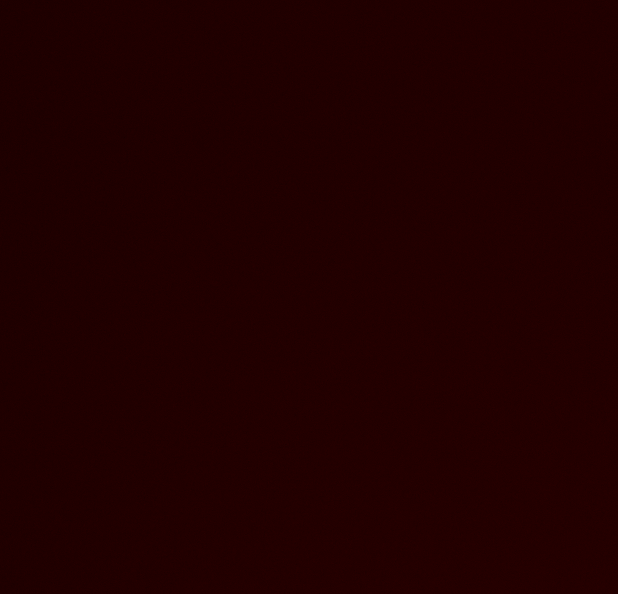

Supplement: Supplementary file 7 — EV Figures Source Data [file 44318_2024_293_MOESM7_ESM.zip › SD Figure EV3/EV3 C&D/20uM YB2 in 300mM NaCl.tif]

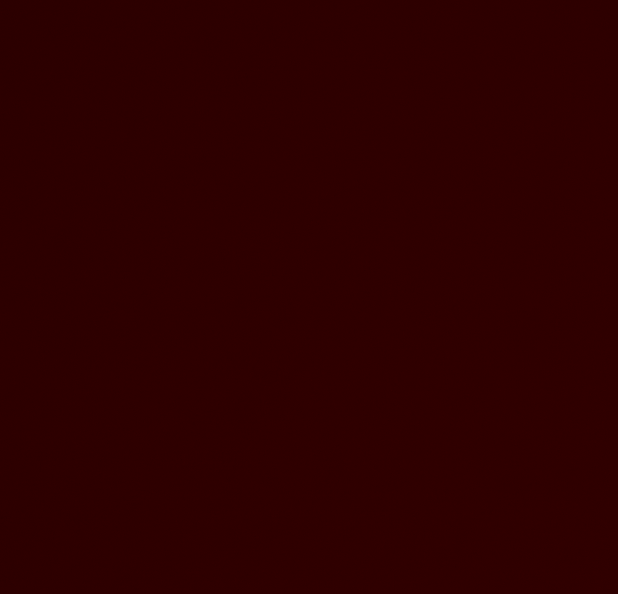

Supplement: Supplementary file 7 — EV Figures Source Data [file 44318_2024_293_MOESM7_ESM.zip › SD Figure EV3/EV3 C&D/20uM YB2 in 450mM NaCl.tif]

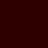

Supplement: Supplementary file 7 — EV Figures Source Data [file 44318_2024_293_MOESM7_ESM.zip › SD Figure EV3/EV3 C&D/20uM YB2 in 450mM NaCl_zoom in.tif]

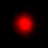

Supplement: Supplementary file 7 — EV Figures Source Data [file 44318_2024_293_MOESM7_ESM.zip › SD Figure EV3/EV3 C&D/5uM YB2 in 100mM NaCl-zoom in.tif]

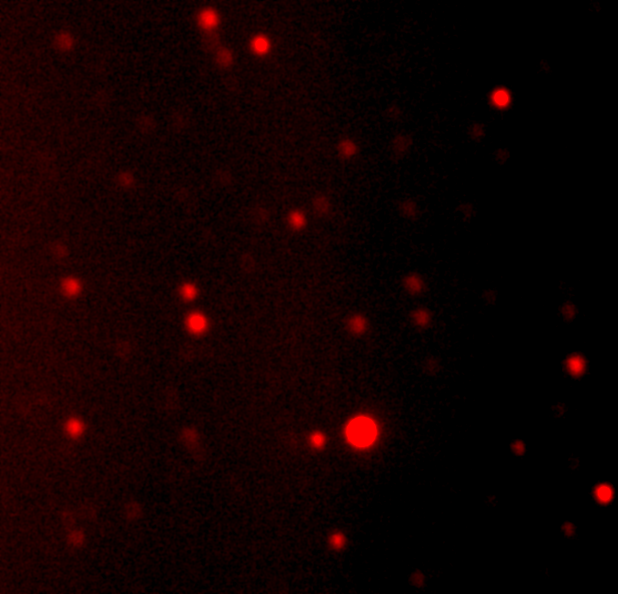

Supplement: Supplementary file 7 — EV Figures Source Data [file 44318_2024_293_MOESM7_ESM.zip › SD Figure EV3/EV3 C&D/5uM YB2 in 100mM NaCl.tif]

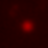

Supplement: Supplementary file 7 — EV Figures Source Data [file 44318_2024_293_MOESM7_ESM.zip › SD Figure EV3/EV3 C&D/5uM YB2 in 150mM NaCl-zoom in.tif]

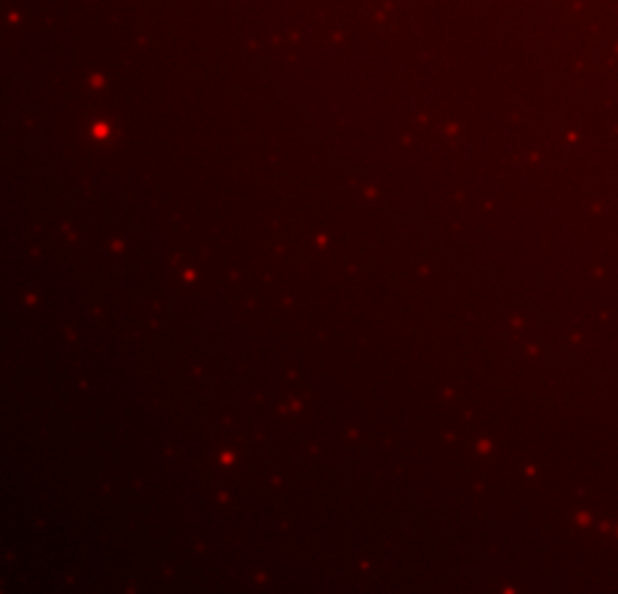

Supplement: Supplementary file 7 — EV Figures Source Data [file 44318_2024_293_MOESM7_ESM.zip › SD Figure EV3/EV3 C&D/5uM YB2 in 150mM NaCl.tif]

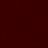

Supplement: Supplementary file 7 — EV Figures Source Data [file 44318_2024_293_MOESM7_ESM.zip › SD Figure EV3/EV3 C&D/5uM YB2 in 300mM NaCl-zoom in.tif]

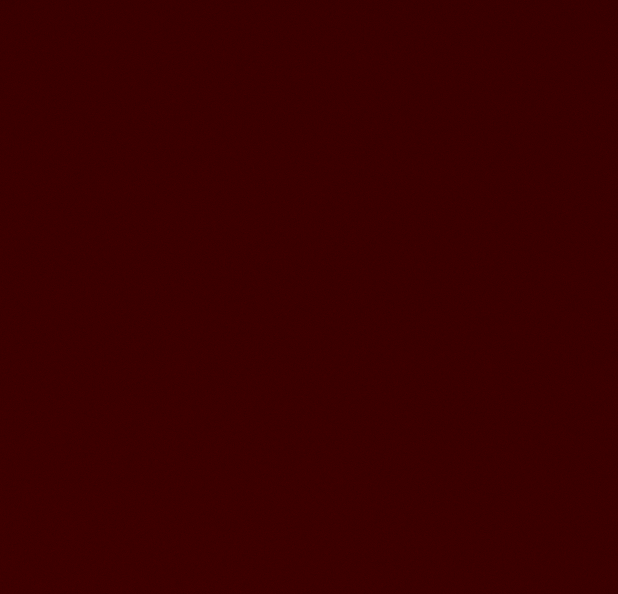

Supplement: Supplementary file 7 — EV Figures Source Data [file 44318_2024_293_MOESM7_ESM.zip › SD Figure EV3/EV3 C&D/5uM YB2 in 300mM NaCl.tif]

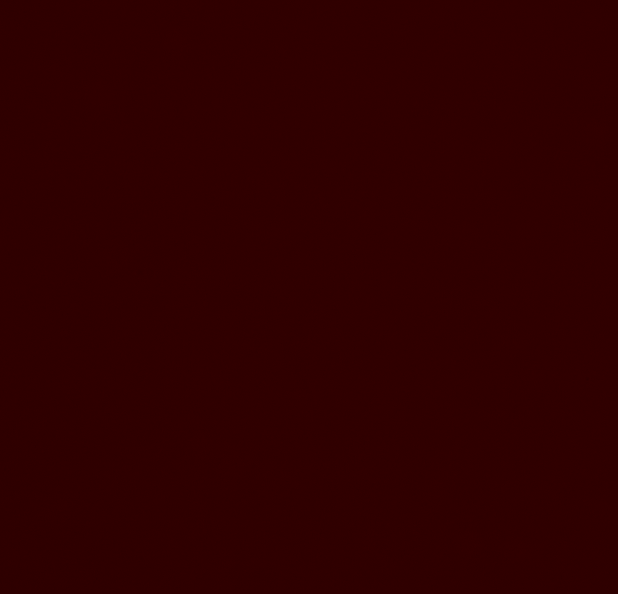

Supplement: Supplementary file 7 — EV Figures Source Data [file 44318_2024_293_MOESM7_ESM.zip › SD Figure EV3/EV3 C&D/5uM YB2 in 450mM NaCl.tif]

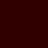

Supplement: Supplementary file 7 — EV Figures Source Data [file 44318_2024_293_MOESM7_ESM.zip › SD Figure EV3/EV3 C&D/5uM YB2 in 450mM NaCl_zoom in.tif]

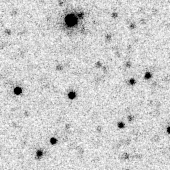

Supplement: Supplementary file 7 — EV Figures Source Data [file 44318_2024_293_MOESM7_ESM.zip › SD Figure EV3/EV3 E/mcherry-CO+YB2-Alexa647_mcherry-CO.tif]

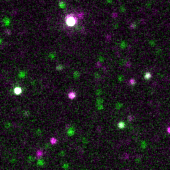

Supplement: Supplementary file 7 — EV Figures Source Data [file 44318_2024_293_MOESM7_ESM.zip › SD Figure EV3/EV3 E/mcherry-CO+YB2-Alexa647_merged.tif]

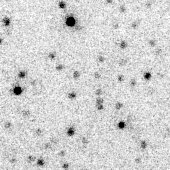

Supplement: Supplementary file 7 — EV Figures Source Data [file 44318_2024_293_MOESM7_ESM.zip › SD Figure EV3/EV3 E/mcherry-CO+YB2-Alexa647_YB2-Alexa647.tif]

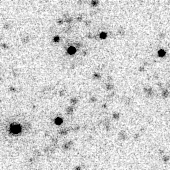

Supplement: Supplementary file 7 — EV Figures Source Data [file 44318_2024_293_MOESM7_ESM.zip › SD Figure EV3/EV3 E/mcherry-CO+YC9-GFP_mcherry-CO.tif]

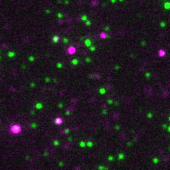

Supplement: Supplementary file 7 — EV Figures Source Data [file 44318_2024_293_MOESM7_ESM.zip › SD Figure EV3/EV3 E/mcherry-CO+YC9-GFP_merged.tif]

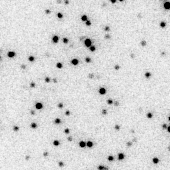

Supplement: Supplementary file 7 — EV Figures Source Data [file 44318_2024_293_MOESM7_ESM.zip › SD Figure EV3/EV3 E/mcherry-CO+YC9-GFP_YC9-GFP.tif]

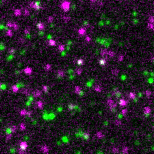

Supplement: Supplementary file 7 — EV Figures Source Data [file 44318_2024_293_MOESM7_ESM.zip › SD Figure EV3/EV3 E/YB2-Alexa647+YC9-GFP_merged.tif]

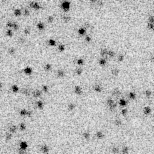

Supplement: Supplementary file 7 — EV Figures Source Data [file 44318_2024_293_MOESM7_ESM.zip › SD Figure EV3/EV3 E/YB2-Alexa647+YC9-GFP_YB2-Alexa647.tif]

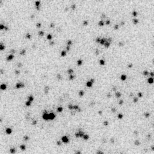

Supplement: Supplementary file 7 — EV Figures Source Data [file 44318_2024_293_MOESM7_ESM.zip › SD Figure EV3/EV3 E/YB2-Alexa647+YC9-GFP_YC9-GFP.tif]

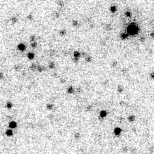

Supplement: Supplementary file 7 — EV Figures Source Data [file 44318_2024_293_MOESM7_ESM.zip › SD Figure EV3/EV3 F/10nM mcherry-CO alone.tif]

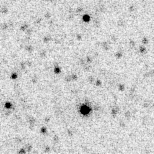

Supplement: Supplementary file 7 — EV Figures Source Data [file 44318_2024_293_MOESM7_ESM.zip › SD Figure EV3/EV3 F/10nM mcherry-CO+10 nM YB2.tif]

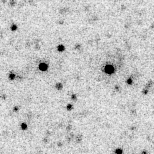

Supplement: Supplementary file 7 — EV Figures Source Data [file 44318_2024_293_MOESM7_ESM.zip › SD Figure EV3/EV3 F/10nM mcherry-CO+100 nM YB2.tif]

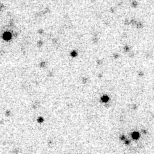

Supplement: Supplementary file 7 — EV Figures Source Data [file 44318_2024_293_MOESM7_ESM.zip › SD Figure EV3/EV3 F/10nM mcherry-CO+100nM YC9-GFP.tif]

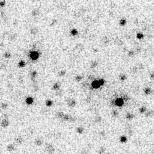

Supplement: Supplementary file 7 — EV Figures Source Data [file 44318_2024_293_MOESM7_ESM.zip › SD Figure EV3/EV3 F/10nM mcherry-CO+10nM YC9-GFP.tif]

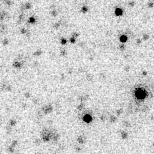

Supplement: Supplementary file 7 — EV Figures Source Data [file 44318_2024_293_MOESM7_ESM.zip › SD Figure EV3/EV3 F/10nM mcherry-CO+600 nM YB2.tif]

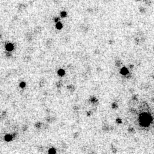

Supplement: Supplementary file 7 — EV Figures Source Data [file 44318_2024_293_MOESM7_ESM.zip › SD Figure EV3/EV3 F/10nM mcherry-CO+600nM YC9-GFP.tif]

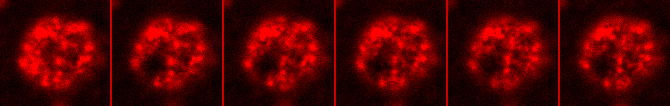

Supplement: Supplementary file 7 — EV Figures Source Data [file 44318_2024_293_MOESM7_ESM.zip › SD Figure EV3/EV3 H/mCherry-CO+YB2 (mCherry-CO images).tif]

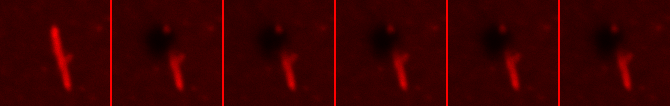

Supplement: Supplementary file 7 — EV Figures Source Data [file 44318_2024_293_MOESM7_ESM.zip › SD Figure EV3/EV3 H/mCherry-CO+YC9-GFP (mCherry-CO images).tif]

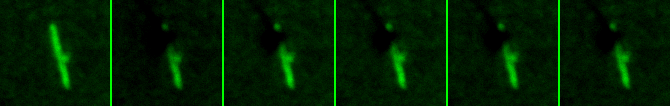

Supplement: Supplementary file 7 — EV Figures Source Data [file 44318_2024_293_MOESM7_ESM.zip › SD Figure EV3/EV3 H/mCherry-CO+YC9-GFP (YC9-GFP images).tif]
