# Supplementary material for: Molecular condensation of the CO/NF-YB/NF-YC/FT complex gates floral transition in Arabidopsis
Source: EMBO J. 2024 Nov 20;44(1):225–50. doi: 10.1038/s44318-024-00293-0 (PMC11696179; doi:10.1038/s44318-024-00293-0)
Supplement: Supplementary file 8 — Expanded View Figures [file 44318_2024_293_MOESM8_ESM.pdf]

Expanded View Figures

Figure EV1. CO proteins accumulate and assemble to condensates in response to light.

(**A**) Time course of light-dependent CO condensate formation in 5-day-old transgenic *Arabidopsis* seedlings (*35S:mCherry-CO*, *35S:mCherry-CO ycT*, and *35S:mCherry-CO 35S:NF-YC9-GFP ycT*) with 15 h darkness treatment. Scale bars, 5 µm. (**B**) Quantification of mCherry-CO cluster index in (**A**). $n = 0, 0, 0, 42, 37, 43, 59, 48, 45$ ROIs (1 ROI in each nucleus) from left to right chart. Error bars, means ± SD. Significant differences among different groups were determined by one-way ANOVA test. (**C**) NF-YC9-GFP displayed a diffuse fluorescence signal pattern in 5-day-old *35S:NF-YC9-GFP ycT* root epidermal cell. Noted there is no CO expression here as endogenous CO is not expressed in root epidermal cells. Scale bars, 2 µm. (**D**) A framework showing the procedures of computer simulation to understand oligomerized CO-NF-Y complex binding on *FT* promotor fragments. Here, the structures of monomeric, trimeric, tetrameric and pentameric CO were first predicted by Alpha-Fold2. Subsequently, the resolved structure of CO-CCT-NF-Y complex (PDB: 7CVO) was applied to replace the CCT domains in the multimeric CO structure. The resulting multimeric CO-NF-Y complexes were then modeled together with the *FT* promoter DNA fragments containing the four binding motif: P1, P2, CORE1, CORE2, colored for highlight, in the simulation boxes with same size, in which the proteins and DNA were shown after the simulation reaching equilibrium. The number of CO-NF-Y molecule and DNA fragment were fixed at 60 and 15, respectively, though the number of CO-NF-Y oligomers were not constant. (**E**) The distribution of the number of bound CO-NF-Y on each CO binding motif, as shown in d, in the simulation box modeling monomeric CO-NF-Y complex and DNA. Source data are available online for this figure.

 © The Author(s)

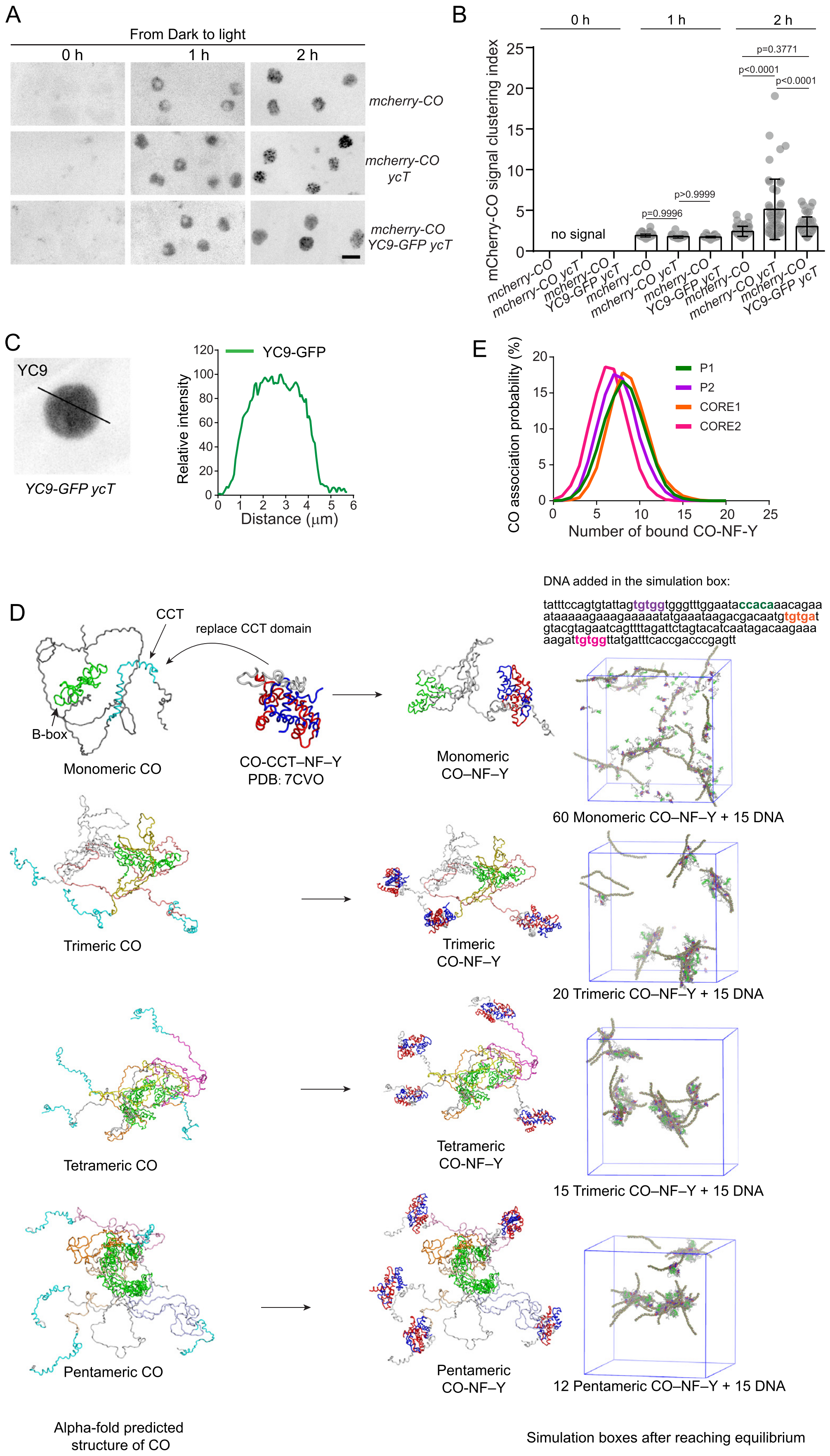
A
From Dark to light
0 h
1 h
2 h
mcherry-CO
mcherry-CO ycT
mcherry-CO YC9-GFP ycT
B
0 h
1 h
2 h
mCherry-CO signal clustering index
no signal
p=0.9996
p>0.9999
p=0.3771
p<0.0001
p<0.0001
mcherry-CO
mcherry-CO ycT
mcherry-CO YC9-GFP ycT
C
YC9
YC9-GFP ycT
YC9-GFP
Relative intensity
Distance (μm)
E
CO association probability (%)
P1
P2
CORE1
CORE2
Number of bound CO-NF-Y
D
CCT
replace CCT domain
B-box
Monomeric CO
CO-CCT–NF–Y
PDB: 7CVO
Monomeric CO–NF–Y
DNA added in the simulation box:
tatttccagtgtattagtgtggtgggtttggaatacccacaaacagaa
ataaaaagaaagaaaaatatgaaataagacgacaatgtgtgat
gtacgtagaatcagttttagattctagtacatcaatagacaagaaa
aagattgtggttatgatttcaccgacccgagtt
60 Monomeric CO–NF–Y + 15 DNA
Trimeric CO
Trimeric CO-NF–Y
20 Trimeric CO–NF–Y + 15 DNA
Tetrameric CO
Tetrameric CO-NF–Y
15 Trimeric CO–NF–Y + 15 DNA
Pentameric CO
Pentameric CO-NF–Y
12 Pentameric CO–NF–Y + 15 DNA
Alpha-fold predicted structure of CO
Simulation boxes after reaching equilibrium

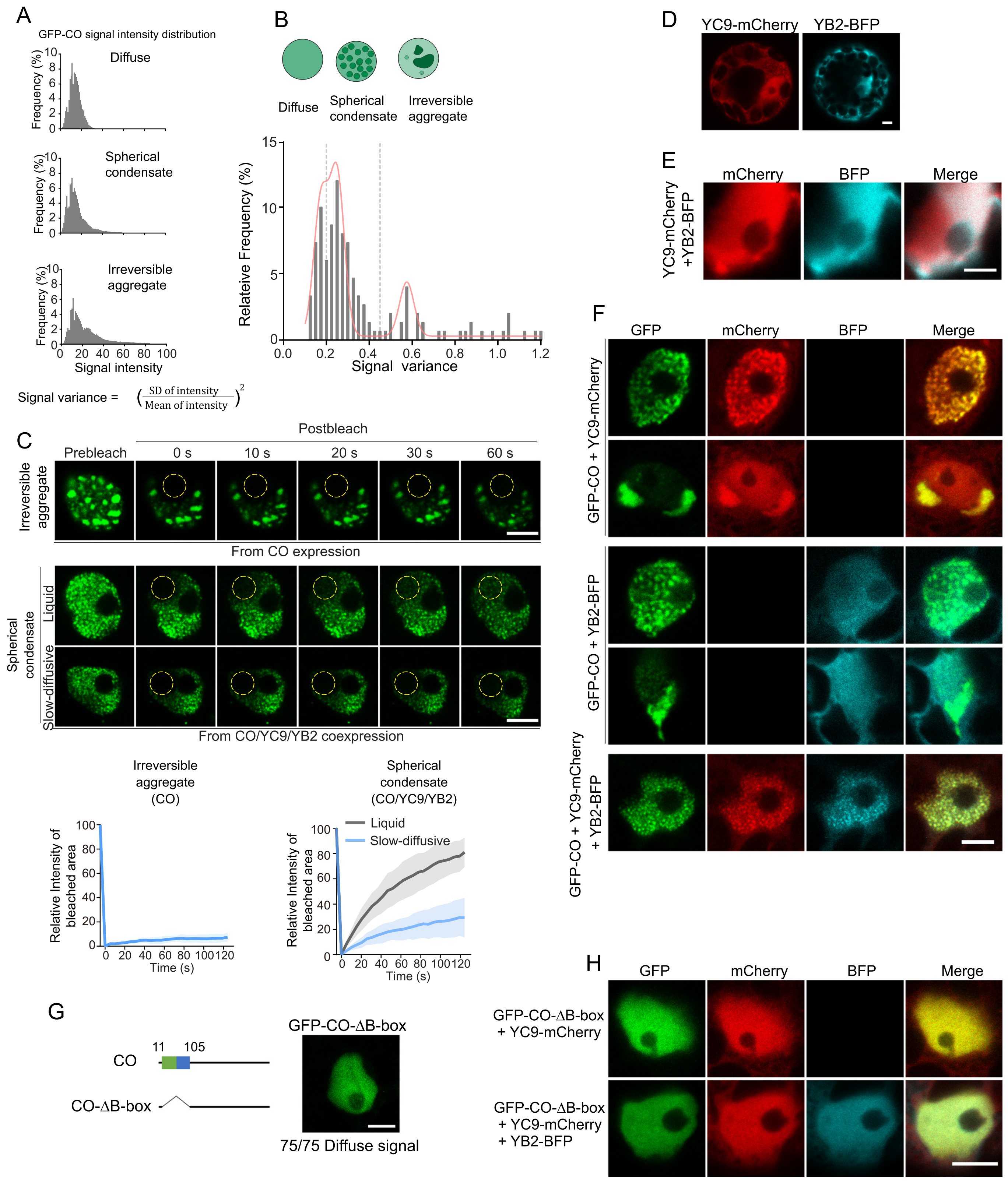
A
GFP-CO signal intensity distribution
Diffuse
Spherical condensate
Irreversible aggregate
Frequency (%)
Signal intensity
Signal variance = (SD of intensity / Mean of intensity)^2
B
Diffuse
Spherical condensate
Irreversible aggregate
Relateive Frequency (%)
Signal variance
C
Postbleach
Prebleach
0 s
10 s
20 s
30 s
60 s
Irreversible aggregate
From CO expression
Spherical condensate
Liquid
Slow-diffusive
From CO/YC9/YB2 coexpression
Irreversible aggregate (CO)
Spherical condensate (CO/YC9/YB2)
Liquid
Slow-diffusive
Relative Intensity of bleached area
Time (s)
D
YC9-mCherry
YB2-BFP
E
YC9-mCherry +YB2-BFP
mCherry
BFP
Merge
F
GFP
mCherry
BFP
Merge
GFP-CO + YC9-mCherry
GFP-CO + YB2-BFP
GFP-CO + YC9-mCherry + YB2-BFP
G
CO
11
105
CO-ΔB-box
GFP-CO-ΔB-box
75/75 Diffuse signal
H
GFP
mCherry
BFP
Merge
GFP-CO-ΔB-box + YC9-mCherry
GFP-CO-ΔB-box + YC9-mCherry + YB2-BFP

 © The Author(s)

Figure EV2. Characterization of CO assembly.

(**A**) The character of fluorescence signal intensity distribution of diffuse, spherical condensation, and irreversible aggregate of CO assemblies. The equation below shows the calculation of the signal variance in each individual nucleus. (**B**) Plotting of CO signal variance distribution after collecting the overall CO signal variance data from different combination of CO, NF-YC9, NF-YB2 co-expression. The variances of fluorescence signal intensities were peaked and thus were classified to three populations: diffuse CO (signal variance < 0.21), spherical CO condensation (signal variance 0.21–0.45), and irreversible CO aggregate signals (signal variance > 0.45). $n = 150$. (**C**) FRAP assay showing unrecoverable CO signal in the irreversible aggregate of CO assembly as in (**B**) and the liquid/slow-diffusive spherical condensates of CO on CO/YC9/YB2 coexpression, respectively. Time indicates the duration after the photobleaching pulse. Dash circles indicate the bleached areas. Recovery curves from the intensity quantification of the bleached area was plotted. $n = 5$, 11 of independent observations from left to right graph. Solid lines and shaded areas represent means ± SD. Scale bars, 5 μm. (**D**) Subcellular localization of NF-YC9-mCherry and NF-YB2-BFP in *Arabidopsis* protoplasts, respectively. Scale bars, 5 μm. (**E**) Co-expression of NF-YC9-mCherry and NF-YB2-BFP in nuclei of *Arabidopsis* protoplasts. Scale bars, 5 μm. (**F**) Co-expression of GFP-CO and NF-YC9-mCherry, GFP-CO and NF-YB2-BFP, and GFP-CO, NF-YC9-mCherry and NF-YB2-BFP in nuclei of *Arabidopsis* protoplasts. Scale bars, 5 μm. (**G**) Schematic of *CO* and *CO-ΔB-boxes* (deletion of two B-boxes, which are indicated by green and blue boxes) constructs. Subcellular localization of GFP-CO-ΔB-boxes in *Arabidopsis* protoplasts. Scale bars, 5 μm. (**H**) Co-expression of GFP-CO-ΔB-boxes and NF-YC9-mCherry, and GFP-CO-ΔB-boxes, NF-YC9-mCherry and NF-YB2-BFP in nuclei of *Arabidopsis* protoplasts. Scale bars, 5 μm. Source data are available online for this figure.

© The Author(s)

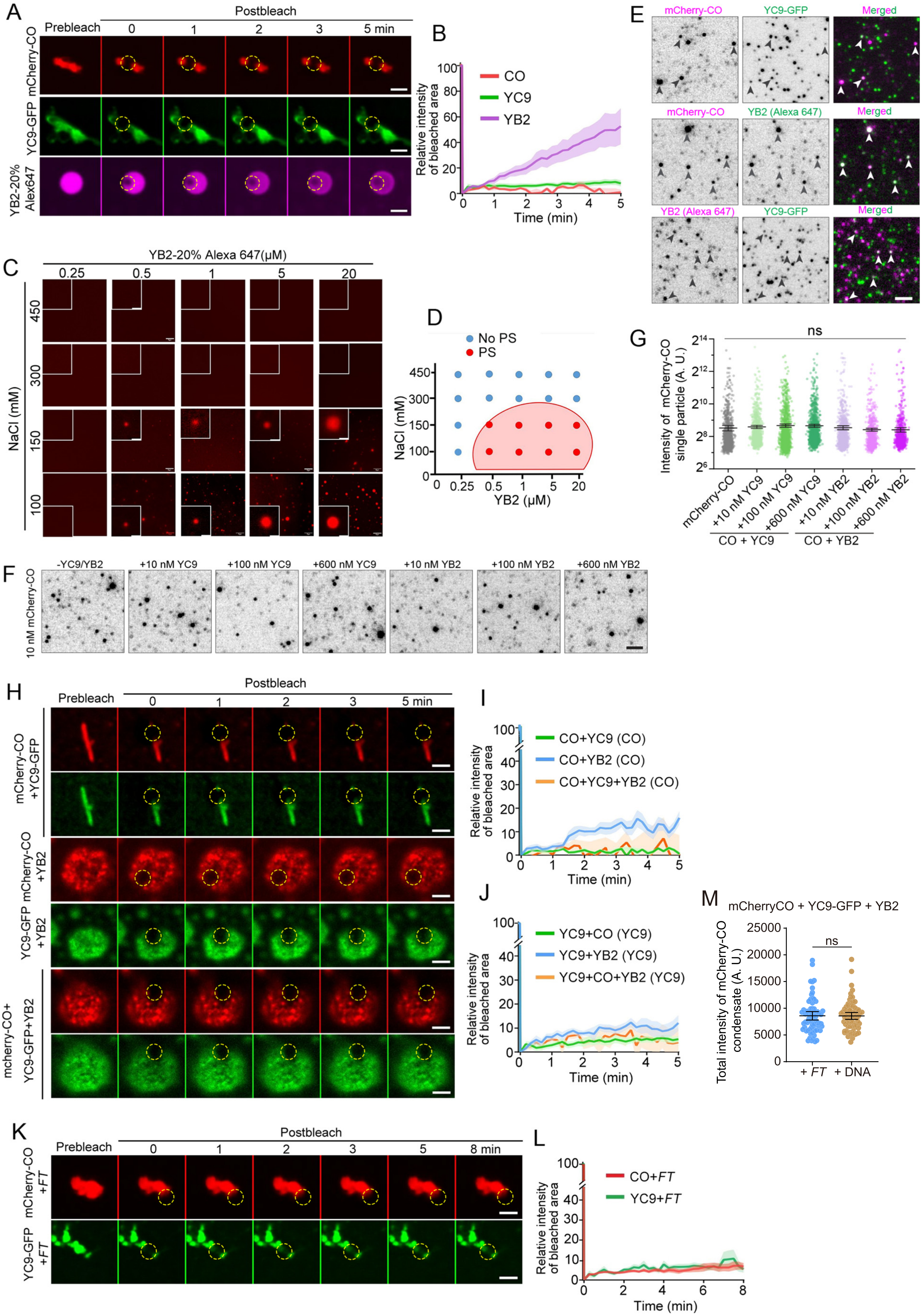
A
Prebleach
Postbleach
0 1 2 3 5 min
mCherry-CO
YC9-GFP
YB2-20% Alex647
B
Relative intensity of bleached area
CO
YC9
YB2
Time (min)
C
YB2-20% Alexa 647(μM)
0.25 0.5 1 5 20
NaCl (mM)
450 300 150 100
D
No PS
PS
NaCl (mM)
YB2 (μM)
E
mCherry-CO
YC9-GFP
Merged
YB2 (Alexa 647)
F
-YC9/YB2
+10 nM YC9
+100 nM YC9
+600 nM YC9
+10 nM YB2
+100 nM YB2
+600 nM YB2
10 nM mCherry-CO
G
ns
Intensity of mCherry-CO single particle (A. U.)
CO + YC9
CO + YB2
H
mCherry-CO +YC9-GFP
mCherry-CO +YB2
YC9-GFP +YB2
mcherry-CO+ YC9-GFP+YB2
I
CO+YC9 (CO)
CO+YB2 (CO)
CO+YC9+YB2 (CO)
J
YC9+CO (YC9)
YC9+YB2 (YC9)
YC9+CO+YB2 (YC9)
M
mCherryCO + YC9-GFP + YB2
Total intensity of mCherry-CO condensate (A. U.)
+ FT
+ DNA
K
mCherry-CO +FT
YC9-GFP +FT
L
CO+FT
YC9+FT
Time (min)

 © The Author(s)

Figure EV3. The condensation properties of mCherry-CO, NF-YC9-GFP, and NF-YB2 assemblies in vitro at nano- or micro-molar scale.

(**A**) FRAP of 0.25 μM mCherry-CO, 0.25 μM NF-YC9-GFP, and 15 μM NF-YB2 with Alex647. All the proteins, including the following experiments, were diluted in 20 mM HEPES, 150 mM NaCl, pH 7.4 buffer. Scale bar, 2 μm. (**B**) FRAP recovery plot of 0.25 μM of mCherry-CO ($n = 5$), 0.25 μM of NF-YC9-GFP ($n = 6$), and 15 μM of NF-YB2 with Alex647 ($n = 14$), respectively. (**C**, **D**) Phase diagram of NF-YB2. The size of NF-YB2 droplets is dependent on the concentration of protein (0.25 μM to 20 μM) and salt (100 mM to 450 mM). Scale bar, 5 μm. (**E**) Single particle image of recombinant mCherry-CO, NF-YC9-GFP, and NF-YB2 (labeled by Alexa fluor 647), indicating the intermolecular interaction of any two of those three components. 10 nM indicated two proteins were mixed in the buffer of 20 mM HEPES, 150 mM NaCl, pH 7.4 for 5 min before being added on cover glass and then checked under TIRFM. Merged image shows the colocalization of indicated two components. Arrow heads indicate the colocalized foci and the related protein single particles in each channel. Scale bar, 2 μm. (**F**) Single particle images of 10 nM recombinant mCherry-CO mixed with a series of concentrations of YC9-GFP or NF-YB2. The proteins were mixed in the buffer of 20 mM HEPES, 150 mM NaCl, pH 7.4 for 5 min before being added on cover glass and then checked under TIRFM. Scale bar, 2 μm. (**G**) Quantification of the mCherry-CO single particle total intensity in (**E**). $n > 700$ single particles in each chart. The middle lines indicate the mean values. Error bars represent 95% confidence intervals. No signal difference was detected over each group by one-way ANOVA test. (**H**) FRAP of mixed droplets of 0.25 μM of mCherry-CO, 0.25 μM of NF-YC9-GFP, and 15 μM of NF-YB2 in pairs or together. Scale bar, 2 μm. (**I**) FRAP recovery plot of mCherry-CO in the mixed droplets of 0.25 μM mCherry-CO/0.25 μM NF-YC9-GFP ($n = 6$), 0.25 μM mCherry-CO/15 μM NF-YB2 ($n = 11$), and 0.25 μM mCherry-CO/0.25 μM NF-YC9-GFP/15 μM NF-YB2 ($n = 14$), respectively. (**J**) FRAP recovery plot of NF-YC9-GFP in the mixed droplets of 0.25 μM mCherry-CO/0.25 μM NF-YC9-GFP ($n = 6$), 0.25 μM NF-YC9-GFP/15 μM NF-YB2 ($n = 10$), and 0.25 μM mCherry-CO/0.25 μM NF-YC9-GFP/15 μM NF-YB2 ($n = 14$), respectively. (**K**) FRAP of mixed droplets of 0.25 μM mCherry-CO/5 μg/mL *FT* and 0.25 μM NF-YC9-GFP/5 μg/mL *FT*, respectively. Scale bar, 2 μm. (**L**) FRAP recovery plot of mCherry-CO in the mixed droplets of 0.25 μM mCherry-CO/5 μg/mL *FT* ($n = 19$), and NF-YC9-GFP in the mixed droplets of 0.25 μM NF-YC9-GFP/5 μg/mL *FT* ($n = 16$), respectively. (**M**) Quantification of total intensity of mCherry-CO condensates in Fig. 3E. $n = 67$, 73 from left to right. The middle lines indicate the mean values. Error bars represent 95% confidence intervals. ns indicate no significant differences (Student's t test). Noted for all the FRAP assays in (**A**), (**H**), and (**K**), the time indicates the duration after the photobleaching pulse and the dash circles indicate the bleached areas. The solid lines and shaded areas in (**B**), (**I**), (**J**), and (**L**) represent means ± SD. Source data are available online for this figure.

© The Author(s)

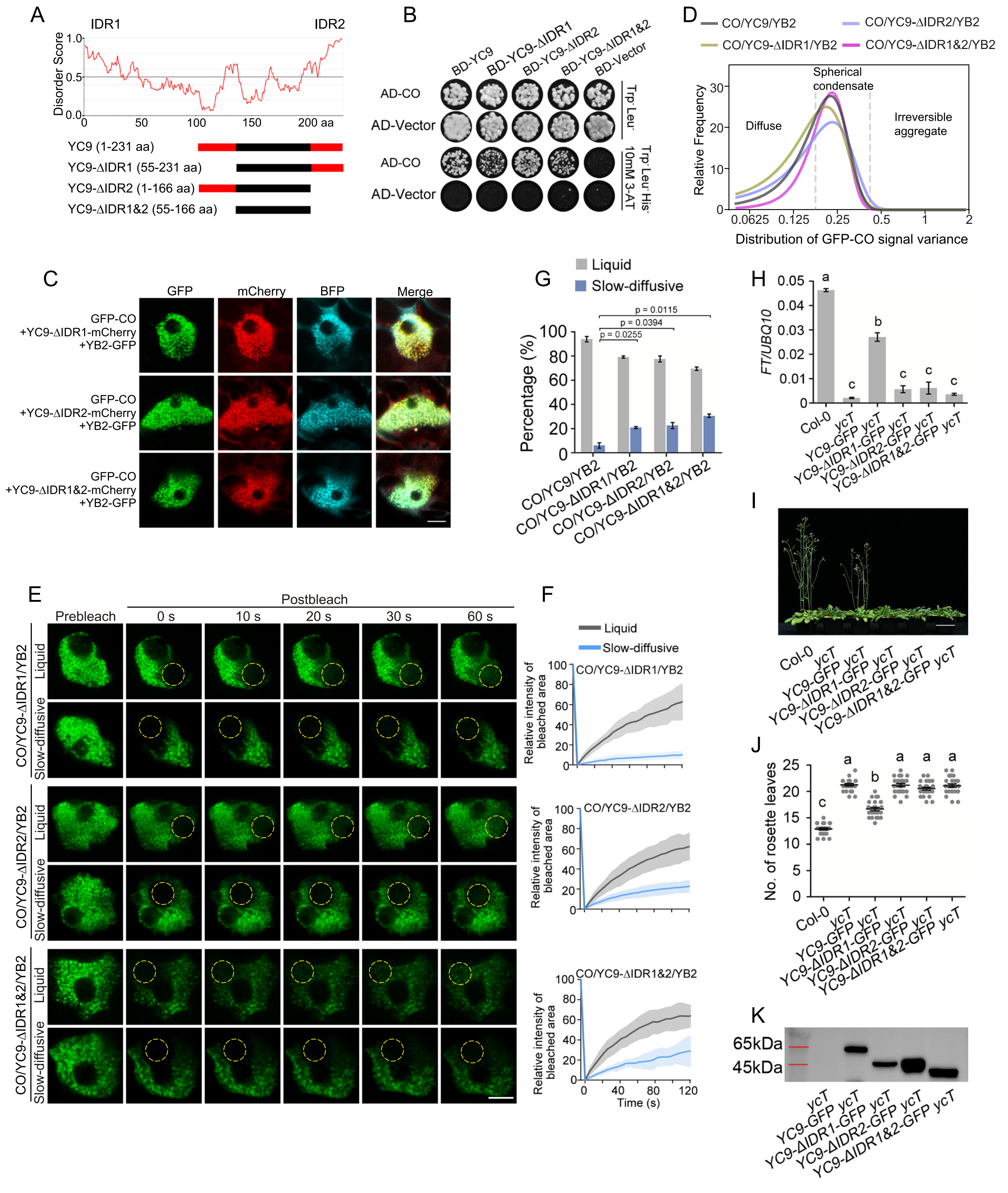
A
IDR1
IDR2
Disorder Score
YC9 (1-231 aa)
YC9-ΔIDR1 (55-231 aa)
YC9-ΔIDR2 (1-166 aa)
YC9-ΔIDR1&2 (55-166 aa)
B
BD-YC9
BD-YC9-ΔIDR1
BD-YC9-ΔIDR2
BD-YC9-ΔIDR1&2
BD-Vector
AD-CO
AD-Vector
10mM 3-AT
D
CO/YC9/YB2
CO/YC9-ΔIDR1/YB2
CO/YC9-ΔIDR2/YB2
CO/YC9-ΔIDR1&2/YB2
Spherical condensate
Diffuse
Irreversible aggregate
Relative Frequency
Distribution of GFP-CO signal variance
C
GFP
mCherry
BFP
Merge
GFP-CO +YC9-ΔIDR1-mCherry +YB2-GFP
GFP-CO +YC9-ΔIDR2-mCherry +YB2-GFP
GFP-CO +YC9-ΔIDR1&2-mCherry +YB2-GFP
G
Liquid
Slow-diffusive
p = 0.0115
p = 0.0394
p = 0.0255
Percentage (%)
CO/YC9/YB2
CO/YC9-ΔIDR1/YB2
CO/YC9-ΔIDR2/YB2
CO/YC9-ΔIDR1&2/YB2
H
FT/UBQ10
Col-0
ycT
YC9-GFP ycT
YC9-ΔIDR1-GFP ycT
YC9-ΔIDR2-GFP ycT
YC9-ΔIDR1&2-GFP ycT
I
E
Prebleach
Postbleach
0 s
10 s
20 s
30 s
60 s
CO/YC9-ΔIDR1/YB2
CO/YC9-ΔIDR2/YB2
CO/YC9-ΔIDR1&2/YB2
Liquid
Slow-diffusive
F
Relative intensity of bleached area
Time (s)
J
No. of rosette leaves
K
65kDa
45kDa

 © The Author(s)

Figure EV4. IDRs within NF-YC9 are required for CO phase separation and its function in flowering control.

(**A**) Sequence analysis of NF-YC9, containing two IDR domains. Schematic of *NF-YC9* and *NF-YC9-ΔIDR mutants* (deletion of IDR1, IDR2, or both) constructs. (**B**) Yeast two-hybrid assays show the interactions between CO and NF-YC9, and CO and NF-YC9-ΔIDR mutants. Transformed yeast cells were grown on SD/Trp$^-$/Leu$^-$ and SD/Trp$^-$/Leu$^-$/His$^-$ (containing 10 mM 3-AT) medium. AD/BD-vector, vector-only controls; AD, activation domain; BD, DNA-binding domain. (**C**) Subcellular localization of GFP-CO, NF-YB2-BFP, and NF-YC9-ΔIDRs-mCherry in *Arabidopsis* protoplasts. Scale bars, 5 µm. (**D**) The distribution of GFP-CO signal variance under indicated combinations of transient expression, subjecting to Gaussian fitting. Dashes lines indicate the cut off on signal variance values to classify the CO assembly to diffuse, spherical condensation and irreversible aggregate. $n = 75$. (**E**) FRAP assays for the spherical GFP-CO condensates in the nucleus with CO/NF-YC9-ΔIDRs/NF-YB2 co-expression. Time indicates the duration after the photobleaching pulse. Dash circles indicate the bleached areas. Scale bar, 5 µm. (**F**) FRAP recovery curves from the intensity quantification of the bleached area in (**E**). Data from ≥7 and 4 of independent observations were plotted for liquid and slow-diffusive condensates, respectively. Solid lines and shaded areas represent means ± SD. (**G**) Quantification of the distribution ratios of CO spherical condensates showing either liquid or slow-diffusive material property in transient expression under combinations of GFP-CO, NF-YB2-BFP, and NF-YC9 (NF-YC9-ΔIDRs-mCherry) in *Arabidopsis* protoplasts. Error bars, means ± SD, $n = 50$ nuclei. Asterisks indicate significant differences (Student's t test, $*P < 0.05$). (**H**) qRT-PCR analysis of *FT* expression in 5-day-old seedlings of Col-0, *ycT*, *35S:NF-YC9-GFP ycT*, *35S:NF-YC9-ΔIDR1-GFP ycT*, *35S:NF-YC9-ΔIDR2-GFP ycT*, and *35S:NF-YC9-ΔIDR1&2-GFP ycT*. Gene expression levels were normalized to *UBQ10*, acting as an internal control. Error bars, means ± SD, $n = 3$. Different lowercase letters above the columns indicate the significant difference among different groups (one-way ANOVA, $P < 0.0001$). (**I**) Flowering phenotype of Col-0, *ycT*, *35S:NF-YC9-GFP ycT*, *35S:NF-YC9-ΔIDR1-GFP ycT*, *35S:NF-YC9-ΔIDR2-GFP ycT*, and *35S:NF-YC9-ΔIDR1&2-GFP ycT*. Scale bar, 5 cm. (**J**) Comparison of rosette leaf number for the representative transgenic plants in (**I**). $n = 25$ seedlings. Different lowercase letters above the columns indicate the significant difference among different groups (one-way ANOVA, $P < 0.0001$). (**K**) Western blot analysis of NF-YC9-GFP, NF-YC9-ΔIDR1-GFP, NF-YC9-ΔIDR2-GFP, and NF-YC9-ΔIDR1&2-GFP expression in the representative transgenic plants. Source data are available online for this figure.

© The Author(s)

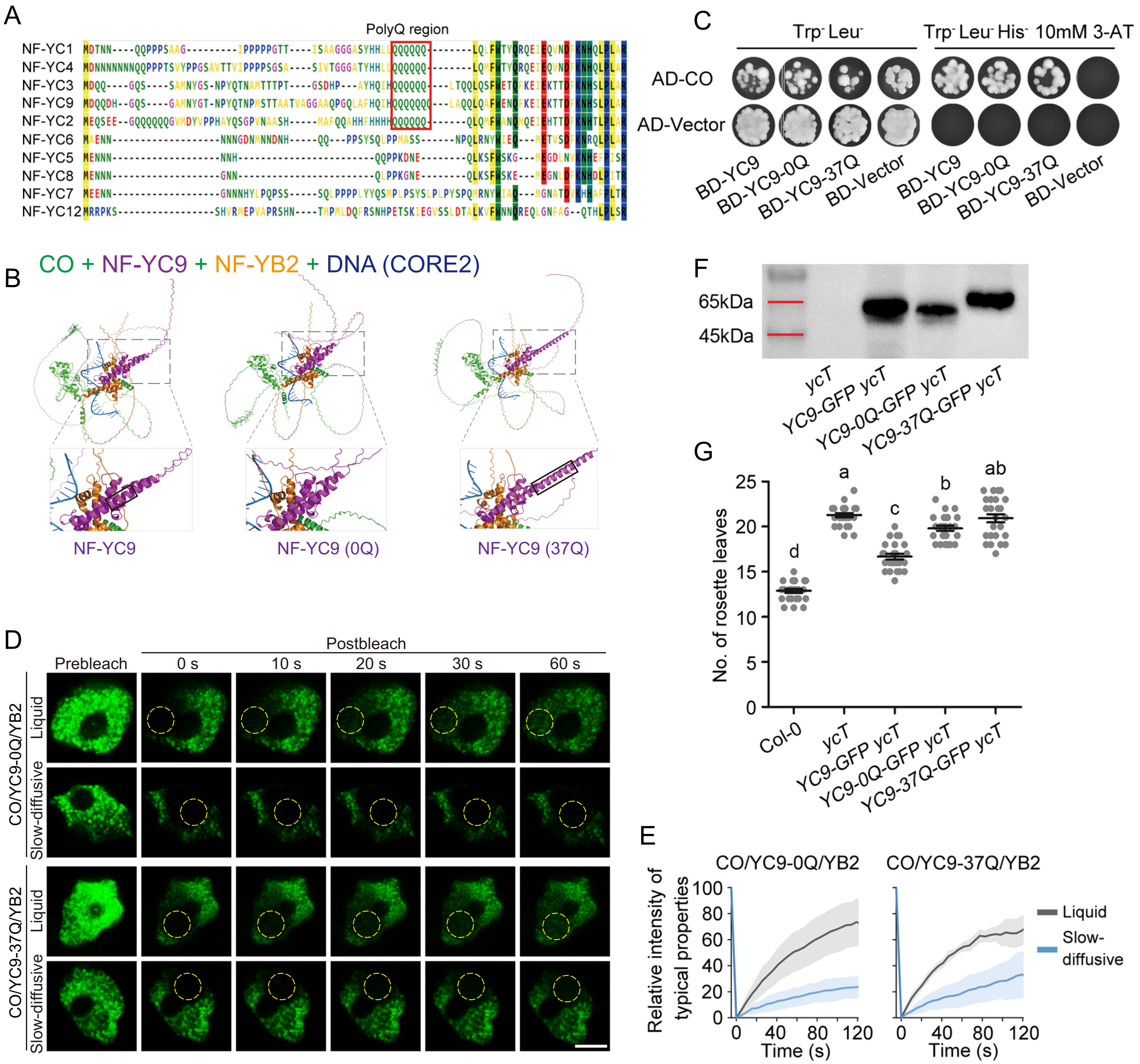

Figure EV5. The polyQ repeat embedded within NF-YC9 IDR is required for mediating CO function in controlling flowering.

(**A**) Sequence analysis of NF-YC family members in *Arabidopsis thaliana* revealed a conserved polyQ repeat among NF-YC1/4/3/9/2, which are related to flowering. (**B**) Structures of CO/NF-YC9/NF-YB2/DNA complex with original NF-YC9, NF-YC9 (0Q) or NF-YC9 (37Q) as predicted by AlphaFold3. The DNA fragment input contains the CORE2 site for CO/NF-Y binding. Zoomed regions show the polyQ region embedded α-helices, the polyQ repeats were highlighted with the black box. (**C**) Yeast two-hybrid assays show the interactions between CO and NF-YC9, and CO and NF-YC9-polyQ mutants. Transformed yeast cells were grown on SD/Trp-/Leu- and SD/Trp-/Leu-/His- (containing 10 mM 3-AT) medium. AD/BD-vector, vector-only controls; AD, activation domain; BD, DNA-binding domain. (**D**) FRAP assays for the spherical GFP-CO condensates in the nucleus with CO/YC9-0Q/YB2 or CO/YC9-37Q/YB2 co-expression. Time indicates the duration after the photobleaching pulse. Dash circles indicate the bleached areas. Scale bar, 5 μm. (**E**) FRAP recovery curves from the intensity quantification of the bleached area in (**D**). Data from ≥7 and 4 of independent observations were plotted for liquid and slow-diffusive condensates, respectively. Solid lines and shaded areas represent means ± SD. (**F**) Western blot analysis of NF-YC9-GFP, NF-YC9-0Q-GFP, and NF-YC9-37Q-GFP expression in the representative transgenic plants. (**G**) Comparison of rosette leaf number of Col-0, *ycT*, *35S:NF-YC9-GFP ycT*, *35S:NF-YC9-0Q-GFP ycT*, and *35S:NF-YC9-37Q-GFP ycT*. Error bars, means ± SD. $n = 25$ seedlings. Different lowercase letters above the columns indicate the significant difference among different groups (one-way ANOVA, $P < 0.0001$). Source data are available online for this figure.

 © The Author(s)
